# Detection of Plant miRNAs Abundance in Human Breast Milk

**DOI:** 10.3390/ijms19010037

**Published:** 2017-12-23

**Authors:** Anna Lukasik, Iwona Brzozowska, Urszula Zielenkiewicz, Piotr Zielenkiewicz

**Affiliations:** 1 Institute of Biochemistry and Biophysics, Polish Academy of Sciences, 02-106 Warsaw, Poland; alukasik@ibb.waw.pl (A.L.); iwonab@ibb.waw.pl (I.B.); ulazet@ibb.waw.pl (U.Z.); 2 Department of Plant Molecular Biology, Institute of Experimental Plant Biology and Biotechnology, University of Warsaw, 02-096 Warsaw, Poland

**Keywords:** plant miRNA, human breast milk, exosomes, cross-kingdom, gene expression regulation

## Abstract

Breast milk is a natural food and important component of infant nutrition. Apart from the alimentary substances, breast milk contains many important bioactive compounds, including endogenous microRNA molecules (miRNAs). These regulatory molecules were identified in various mammalian biological fluids and were shown to be mostly packed in exosomes. Recently, it was revealed that plant food-derived miRNAs are stably present in human blood and regulate the expression of specific human genes. Since then, the scientific community has focused its efforts on contradicting or confirming this discovery. With the same intention, qRT-PCR experiments were performed to evaluate the presence of five plant food-derived miRNAs (miR166a, miR156a, miR157a, miR172a and miR168a) in breast milk (whole milk and exosomes) from healthy volunteers. In whole milk samples, all examined miRNAs were identified, while only two of these miRNAs were confirmed to be present in exosomes. The plant miRNA concentration in the samples ranged from 4 to 700 fM. Complementary bioinformatics analysis suggests that the evaluated plant miRNAs may potentially influence several crucial biological pathways in the infant organism.

## 1. Introduction

Breast milk is perceived as required for optimal growth and development of the infant [1,2]. In addition to supplying the infant with vitamins and nutrients, numerous detailed studies have shown that breast milk contains a wide range of other bioactive compounds that stimulate body growth, brain development, shape the gut microbiota, participate in nutrient absorption, and play a vital role in the appropriate adaptive and innate immune responses [1,3,4,5]. According to other reports, breast milk may also decrease infantile mortality, as well as decrease the risk of obesity and type 2 diabetes [6,7,8,9]. The above-mentioned bioactive compounds are represented by minerals, hormones, immune cells (e.g., leukocytes and macrophages), specific proteins (e.g., immunoglobulins, cytokines, defensins, and lactoferrin), as well as microRNA molecules (miRNAs) [3,5,10,11,12].

miRNAs are a class of small (19–24 nt), single-stranded particles that mediate post-transcriptional gene expression through target mRNA translation inhibition or degradation [13,14,15]. By these means, miRNAs control crucial biological processes, such as metabolism, apoptosis, developmental timing, cell proliferation, immune responses, hormone signaling, differentiation, and many others [16,17,18,19]. In addition to breast milk [11,20], miRNAs have been discovered in several other body fluids, such as plasma, serum, saliva, urine, and tears [21,22,23,24,25]. Furthermore, it was shown that the profiles of miRNAs, especially in the serum, plasma, and urine, are tightly correlated with various diseases and pathological states. This made these molecules suitable as new biomarkers for diagnosis, prognosis, and monitoring patient treatment response [21,26,27,28].

Comparison between breast milk and blood miRNA profiles has suggested that milk miRNAs may originate from the mammary epithelium and maternal circulation [29,30]. According to different studies, milk miRNA profiles vary considerably and expectedly between individuals, probably reflecting the mother’s background, e.g., race, age, health, and lifestyle [31,32]. Differences in milk miRNA profiles were also observed between the different breast milk fractions (e.g., fat, cells, and skim milk) during the lactation period, and even at different times of the day, which may be influenced by the specific infant’s needs at different time points or developmental stages [20,30,32,33,34]. Moreover, recent study has shown that the profile of milk miRNAs is also determined by the date of infant delivery [35]. Many researchers reported that membranous vesicles, named exosomes, that are present in breast milk, are enriched in miRNAs [11,31,36,37,38]. Exosomes are shown to protect these molecules from harsh conditions in the human gastrointestinal (GI) tract. Thus, several groups of scientists have assumed that miRNAs may be transferred into these carriers to the bodies of infants via the GI tract, and are likely to contribute to infant development, as well as protection against infections [11,31,37]. Additionally, it was indicated that exosomal miRNAs play important roles in an infant’s metabolic “programming” and growth [39,40,41]. The presence of bovine-derived miRNA molecules in various milk products was also confirmed by a few groups [42,43,44]. Similar to the human molecules, the miRNAs from raw bovine milk were shown to be packed in exosomes, which protect them from degradation [38,45,46,47]. Nevertheless, the homogenization, pasteurization, fermentation and other processing of milk alter the bovine miRNA concentration [48,49,50]. Baier et al. propose that certain bovine milk-derived miRNAs are bioavailable, and are present in adult human blood after consumption [51]. Moreover, one of these miRNAs, miR-29, was shown to regulate specific human gene expression [51]. 

Intriguingly, the possibility of cross-kingdom gene expression regulation by miRNAs was shown in the work of Zhang et al. in 2012 [52]. The authors reported that plant food-derived miRNAs are present and stable in human blood. In addition, they provide evidence that one of the identified molecules, namely rice MIR168a, can target the mRNA of the low-density lipoprotein receptor adaptor protein 1 (LDLRAP1), and thus reduce its expression level in the blood and liver of rice-fed mice [52]. This, in turn, results in an increase of low-density lipoproteins (LDL) in animal plasma. Zhang et al. also showed that most of the MIR168a present in the serum is packed in microvesicles (MV) [52]. Following the described research, a great number of studies were published and contribute to the argument on whether plant miRNAs can transfer to human circulation and, most of all, whether they are able to effectively regulate gene expression in a cross-kingdom manner. The majority of these up-to-date reports (from both fronts) were reviewed and discussed in the recently published article by Lukasik et al. [53] and Perge et al. [54]. Our group also took part in this debate that continues to the present day. In one of the studies carried out by our group, *in silico* examination was performed to determine if potentially food-derived miRNA molecules that enter the circulatory system can be found in exosomes isolated from mammalian breast milk [55]. Using various computational approaches, an accurate bioinformatics analysis of publicly available libraries generated from the high-throughput sequencing of human and porcine breast milk miRNAs was performed. As a result, 35 and 17 plant miRNAs species were identified in human and porcine exosome samples, respectively [55]. Molecules with the highest abundance level included miR166a, miR951, miR156a, miR168a, and miR472. In the past year, Bagci et al. have questioned these findings, concluding that the discovered plant miRNAs in mammalian milk samples were artifacts [56].

With inspiration from all the above-mentioned findings, an independent in vitro qRT-PCR experiment supported with appropriate controls was performed and showed that the five evaluated plant food-derived miRNAs were present in the human breast milk (whole milk and exosomes) of healthy volunteers. To supplement these results, a bioinformatics analysis was carried out, which proposed that the examined plant miRNAs, namely miR156a, miR167a, miR168a, miR172a, and miR166a, may potentially influence several essential biological pathways in infants. The presented findings are consistent with previous reports by our group and reveal another intriguing potential of plant miRNAs.

## 2. Results

### 2.1. RNA Quality and Concentration

The total RNA from whole human breast milk and exosome fractions was isolated using a commercial kit (column-based method), which prevents the loss of small RNAs [57]. After procedure modification (repetition of phenol-chloroform extraction step), good quality RNA was obtained for all except two (R3 and R5 exosome fraction) of the samples (Table 1). Total RNA extracted from the whole milk samples gave higher concentrations (from 94 to 1018 ng/μL) in comparison to the RNA purified from the exosome fraction (from 21 to 228 ng/μL). The miRNAs were identified on the electropherograms in all examined samples, with varying profiles among them. In the case of the RNA samples extracted from whole milk, the miRNA concentration ranged from 13 to 51 ng/μL, and the estimated percentage ratio of the miRNA to small RNA was approximately 40%. In turn, the miRNA concentration in the RNA samples isolated from the exosomes was between 9 and 37 ng/μL, and the estimated percentage ratio of miRNA to small RNA varied between samples, ranging 19–45%.

### 2.2. Plant miRNAs Are Detectable in Human Breast Milk via qRT-PCR

**Figure 1:**
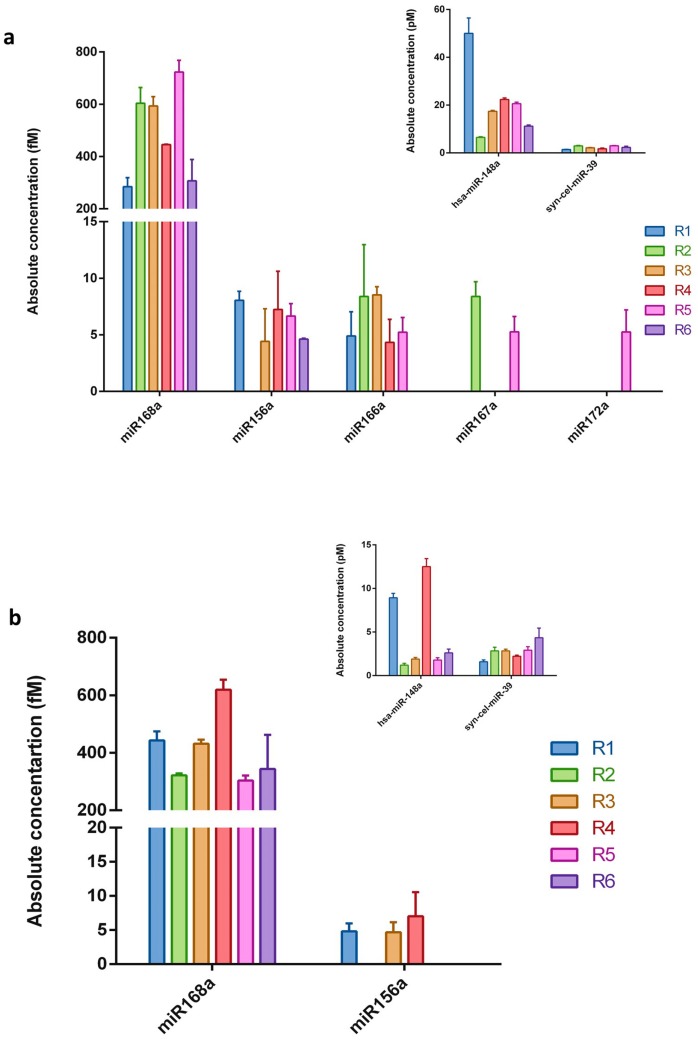
The absolute concentration of the examined plant miRNAs in human breast milk samples. The five selected plant miRNAs were measured by qRT-PCR in human (**a**) whole breast milk and (**b**) exosomes isolated from breast milk samples. Calculations were based on the standard curve generated using the synthetic miR172a molecule. Endogenous hsa-miR-148a and synthetic cel-miR-39 served as controls (inserts). *N* = 6, error bars ± SD.

To experimentally verify whether plant miRNAs are present in human breast milk, five plant miRNAs were selected, namely miR156a, miR172a, miR166a, miR167a, and miR168a. Synthetic cel-miR-39 (intentionally added during isolation procedure) was used for the qRT-PCR technical control and monitoring of the miRNA purification efficiency (Table 1). The 12 RNA samples (extracted from whole milk and the corresponding exosomes fraction) coming from six healthy women were subjected to qRT-PCR analysis using TaqMan^®^ technology that is designed for and commonly used in miRNA studies [34,44,58,59]. The absolute concentration of each miRNA was calculated according to the standard curve generated using a synthetic plant miR172a at known concentrations (Figure S3a). As shown in Figure 1, all the selected plant miRNAs were detected in whole milk samples. However, not all the examined miRNAs were present in each evaluated sample. Whilst, miR168a, miR156a, and miR166a were the most frequently found molecules, miR167a was detected in two samples, and miR172a was only detected in one whole milk sample. Evaluation of the plant miRNA abundance in the RNA samples extracted from exosome fraction revealed only two out of the five plant miRNAs chosen for the study. In addition, miR168a was identified in all six samples and miR156a in three samples. As expected, most of the evaluated plant miRNAs were detected in quite low concentrations, namely in the range of 4.3 to 8.5 fM. In every sample the control, synthetic cel-miR-39, was detected in similar amounts (ranging between 1.4 and 4.3 pM; Figure 1-inserts) following similar added quantities. Human hsa-miR-148a, one of the most highly expressed miRNAs in breast milk exosomes [11,60], was additionally included as an endogenous control for the entire experimental procedure. As can be seen in Figure 1 (inserts), hsa-miR-148a was detected in all samples, in a concentration range of 6.5–50 pM in whole milk samples and 1.2–12.5 pM in exosome fractions. The presented result shows that adequate methodology has been employed throughout the whole experimental procedure. 

The concentrations of miR168a, which were detected in all the examined whole milk and exosome samples, were approximately 100 times higher (300–700 fM) than those of the rest of the examined plant miRNAs. It should be mentioned that negative, no-template controls for reverse transcription and real-time PCR were always performed for all the examined miRNAs (in each experiment), and no Cq value for any of the samples was obtained. Further controls for miR168a were additionally performed. To exclude non-specific signals from the isolation procedure (e.g., contamination connected with reagents used), total RNA isolation was performed from 2 mL of nuclease-free deionized water, instead of milk. The obtained “RNA sample” was used as a template for qRT-PCR. Moreover, real-time PCR reaction with primers specific for miR168a and non-specific complementary DNA (cDNA) matrices (obtained in reverse transcription using total RNA milk sample and stem-loop primers specific for different miRNAs, such as miR172a or syn-cel-miR-39) were also performed. In all the above-described controls, miR168a was not detected.

### 2.3. Relative qRT-PCR

**Figure 2:**
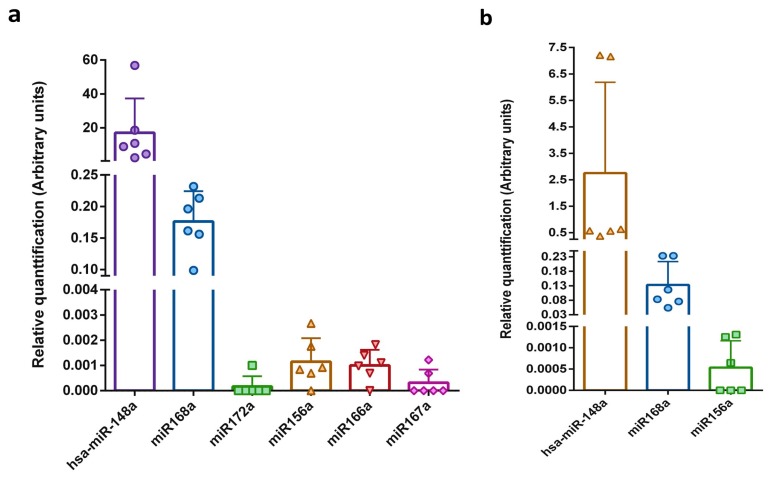
Relative plant miRNAs levels in human breast milk samples. The five selected miRNAs were measured by qRT-PCR in human (**a**) whole breast milk and (**b**) exosomes isolated from milk samples. Calculations were based on normalization to the spike-in synthetic cel-miR-39. The *y*-axes show arbitrary units representing the relative miRNA expression levels. *N* = 6, error bars ± SD.

Exogenous spike-in RNA, such as synthetic *Caenorhabditis elegans* miRNA (cel-miR), can be used not only for monitoring the efficiency of miRNA purification and reverse transcription, but also as a normalization control [29,31,42,58]. A stable reference control is obtained by adding the same amount of spike-in miRNA after the lysis step to the same volume of each examined sample. To date, there is no well-established endogenous control for milk sample normalization, which is mostly due to high expression differences between different lactation stages and fluctuations in the miRNA profile during the day [61]. Hence, to compare the expression levels of miRNAs among the different milk samples, the Cq value of the synthetic cel-miR-39 was used. Data obtained for the whole and exosome fractions of milk are presented separately in Figure 2. The relative level of the detected plant miRNAs was similarly low in all the tested milk samples. As expected, the level of endogenous human hsa-miR-148a was five orders of magnitude greater than the level of plant miRNAs. In accordance with the values presented in the absolute miRNA content analyses, the relative level of miR168a was greater than that of the other plant miRNAs (2 orders of magnitude). 

**Figure 3:**
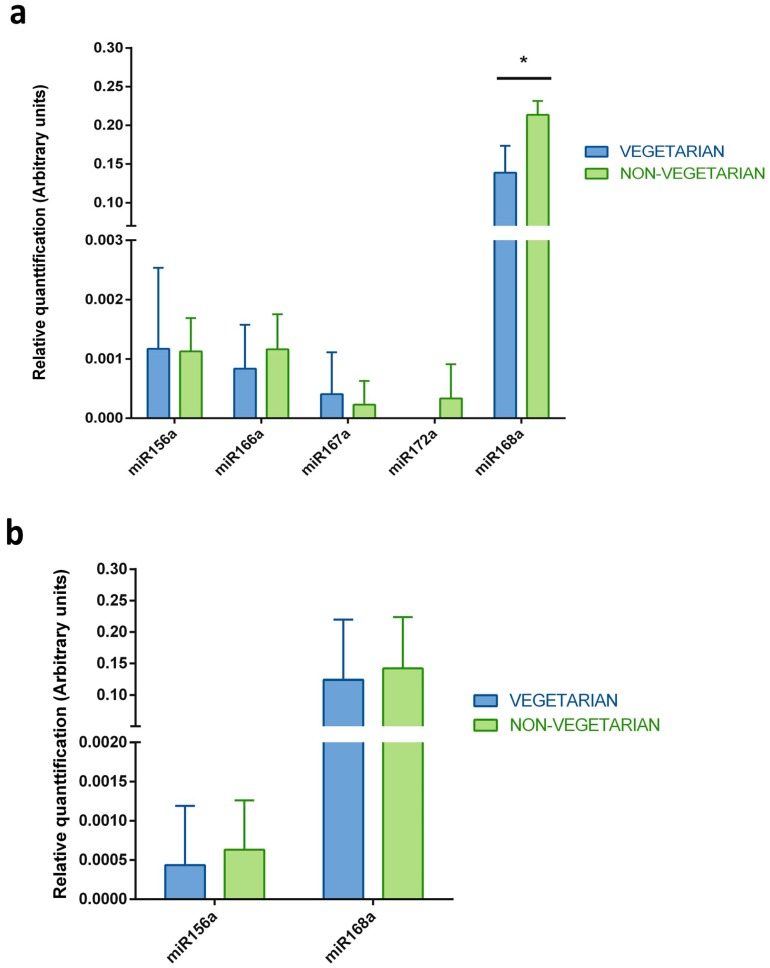
Comparison of the plant miRNAs levels measured in breast milk samples from the vegetarian and non-vegetarian volunteers. The five selected miRNAs were measured by qRT-PCR in (**a**) whole breast milk and (**b**) exosomes isolated from breast milk samples collected from vegetarian and non-vegetarian volunteers. The *y*-axes show arbitrary units representing the relative quantification of the plant miRNAs levels. *N* = 6, unpaired *T*-test, error bars ± SD, * *p*-value < 0.05.

Taking into consideration the fact that breast milk samples were collected from mothers that have varied dietary preferences (vegetarian or non-vegetarian), the evaluation of the differences in the identified plant miRNAs levels between these two groups was performed (Figure 3). There was no statistically significant difference between the plant miRNAs levels in the exosome samples or the whole milk samples obtained from vegetarian and non-vegetarian mothers. In both groups, the plant miRNAs were detected in a similar concentration range. The only two exceptions were observed in the whole milk samples, where miR172a was identified in one sample (R5) from a non-vegetarian mother, and the level of miR168a was statistically significantly lower in vegetarian volunteers than non-vegetarian ones.

### 2.4. In silico Evaluation of the Identified Plant miRNAs Influence on the Infant

The selection and annotation of the miRNA targets are the first steps to determine the potential role of miRNAs in the cell or organism. As initial targets, the 3′ UTR, 5′ UTR and coding sequences representing ~20,100 unique human genes were collected. Target prediction with the use of the tools4miRs service suggested 271 unique targets for miR156a, 88 unique targets for miR166a, 4 unique targets for miR168a, 15 unique targets for miR167a and 7 unique targets for miR172a (Table S1). Most of the plant miRNAs binding sites were in coding sequence regions, then in 3′ UTRs and 5′ UTRs. Functional annotation was carried out, namely, Kyoto Encyclopedia of Genes and Genomes (KEGG) pathway mapping, and showed that the proposed targets are mostly involved in, inter alia: purine metabolism (ko00230; e.g., XDH and PDE1C), RNA transport (ko03013; e.g., NUP85 and XPO5), thyroid hormone signalling (ko04919; e.g., PLCG1), hippo signalling (ko04390; e.g., PPP2R2), calcium signalling (ko04020; e.g., SLC8A1), Th1 and Th2 cell differentiation (ko04685; e.g., NFATC3 and STAT1), and chemokine signaling pathways (ko04062; e.g., CXCR2). The full KEGG pathway mapping results are presented in the Table S2. The performed Gene Ontology (GO) term annotations from the “Biological Process” category additionally revealed that the predicted plant miRNAs interact with targets playing various, important roles such as protein transport (GO:0015031; e.g., RINT1), regulation of transcription from the RNA polymerase II promoter (GO:0006357; e.g., MED22 and MED24), involvement in immune system processes (GO:0002376; e.g., LAIR1), protein phosphorylation (GO:0006468; e.g., CDC42BPA), signal transduction (GO:0007165; e.g., NSMAF), nervous system development (GO:0007399; e.g., NOTCH2 and ARHGAP26), and involvement in apoptotic processes (GO:0006915; e.g., PDCD6). The graph and table presenting the full GO annotations received for 373 targets can be found in Figure S1 and Table S3, respectively. To better recognize the influence of plant miRNAs on infants, statistical GO enrichment analysis was performed, which indicated few significant GO terms functionally describing the predicted human targets: regulation of localization (GO:0032879), regulation of small GTPase mediated signal transduction (GO:0051056), Ras protein signal transduction (GO:0007265), Rac guanyl-nucleotide exchange factor activity (GO:0030676), and enzyme binding (GO: 0019899). Detailed GO enrichment results can be found in a graph form in Figure S2.

## 3. Discussion

An extensive number of studies has shown that breast milk is the gold standard of infant nutrition, and is composed of different bioactive compounds that contribute to early infant development [1,2,3,5,10]. One of these compounds are miRNA molecules [11]. These molecules have been intensively investigated since their discovery in 1993 [62,63]. However, miRNAs, and especially plant miRNAs, gained even more attention after a report describing their ability to regulate gene expression in a cross-kingdom manner. Zhang et al. first provided evidence that food-derived miRNAs stably transfer to the human blood and inhibit the expression of specific human gene [52]. Since Zhang et al.’s discovery, many scientists have focused their efforts on answering some intriguing questions, such as whether cross-kingdom gene expression regulation by plant miRNAs is possible and biologically meaningful. Some of them have successfully confirmed this phenomenon, and have perceived it as an opportunity for novel therapeutic approaches. Others, in turn, are convinced that the mentioned phenomenon is the result of false positive effects, and provide several supporting pieces of evidence for their hypothesis [53]. Aside from the important questions that were raised, there are several others that need to be answered, such as how plant miRNA molecules enter the targets animal cells. One of the possibilities is that they are packed into intracellular carriers, such as exosomes. Endogenously originating miRNAs are highly enriched in exosomes isolated from different body fluids, including breast milk [25,31,36]. Vesicles were shown to protect miRNAs from degradation by RNases. In one of the studies carried out by our group, the publicly available data from the high-throughput sequencing of miRNAs isolated from mammalian breast milk exosomes were analyzed [55]. This in silico study revealed that several plant food-derived miRNAs were present in the analyzed samples and may potentially influence important biological pathways in infants. These positive results, similar to others in the field, were questioned as to whether they showed the effect of contamination and artifacts, rather than real food-derived miRNAs [56]. In the presented study, the independent qRT-PCR experiments were performed to evaluate the abundance of the selected plant miRNAs in human breast milk collected from healthy volunteers. Five plant miRNAs, namely miR168a, miR156a, miR166a, miR172a, and miR167a, were chosen for investigation for three reasons: (1) their presence in mammalian breast milk exosomes revealed by Lukasik et al. [55]; (2) their level of abundance in animal blood/plasma discovered by, e.g., Zhang et al. [52]; and (3) the presence of these miRNAs in plants eaten by volunteers up to 24 h before breast milk sample collection.

Detection of small amounts of miRNAs in serum, plasma or milk samples requires a method with high sensitivity and specificity. This is especially important in studying exogenous miRNAs, such as plant miRNAs, which are believed to be acquired orally through food intake, a hypothesis that has raised a great deal of controversy. Therefore, the qRT-PCR method with the TaqMan^®^ technology was chosen, which ensures high specificity and a lack of false positive results. The detection level of the miRNA molecules may depend also significantly on the real-time PCR instrument used; therefore, it was verified by generating a parallel standard curve on two instruments, LightCycler 480 (Roche, Basel, Switzerland) and Mic (Bio Molecular Systems, Upper Coomera, Queensland, Australia). As can be seen in Figure S3, the Mic real-time cycler detected lower standard concentrations than the LightCycler 480, and therefore all the experiments in this study were performed using this instrument. The use of the Mic real-time cycler enables the detection of plant miRNAs present in low amounts in the examined samples, which could not be possible with the use of the LightCycler 480. All the plant miRNAs chosen for this study were detected in the examined milk samples. There were some differences between the samples as not all the chosen plant miRNAs were present in every sample. In the whole milk samples all the chosen plant miRNAs were detected, while in the samples obtained from the exosome fraction, only miR156a and miR168a were present. As expected, the concentrations of the plant miRNAs were lower than those of the endogenous hsa-miR-148a control, and were in the range of 4.3–8.5 fM. However, for one plant miRNA, namely miR168a, a higher concentration in all the examined samples was detected (up to 700 fM). As many experimental controls were performed, such as the use of 2 mL of water instead of milk for RNA isolation and no-template controls in the RT and qPCRs reactions, contamination, as well as a false-positive result, can be excluded. Efficiency of the miRNAs isolation was in the range of 9–27%, and potentially depended on the sample composition, as the same isolation procedure dedicated for small RNAs was used for all the examined samples. Therefore, it could be inferred that the detected levels of miRNAs are underestimations of the true levels. This may also be a potential explanation why only two plant miRNAs were identified in the exosomes, namely, the isolated miRNA fraction was insufficient to reach the qRT-PCR signal. Additionally, there may be a specific selection mechanism that packs certain miRNAs in the mentioned carriers. These results also indicate an important issue, namely that the false negative effect should be taken into consideration in this type of research (with low concentrations of miRNAs) to avoid further improper conclusions.

Breast milk samples were collected from mothers with varied dietary preferences, specifically, from vegetarians and non-vegetarians. In this manner, it was interesting to evaluate any potential differences in the plant miRNAs patterns between these two groups of volunteers. As shown in the results section and in Figure 3, there was no difference in the miRNAs concentrations in the exosome samples. In the case of the whole milk samples, only the levels of miR172a and miR168a were higher in the non-vegetarian samples than in the vegetarian samples. Additionally, PCA analysis was also performed and it was confirmed that there were no significant similarities in the plant miRNA profiles within the analyzed groups, since samples representing vegetarians and non-vegetarians did not cluster together (Figure S4). One could expect that, regarding the dietary preferences, the breast milk samples collected from the vegetarian mothers should have higher concentrations of plant miRNAs. However, it must be noticed that although three of women participating in the study were vegetarians, all of them ate a variety of plants every day. This could potentially explain the lack of difference between these groups. Another possibility is that non-vegetarian mothers ate more plant food in the time before breast milk sample collection, or that the analyzed number of samples is simply too small to indicate any statistically significant difference in the levels of the identified plant miRNAs.

As mentioned at the beginning of this discussion, breast milk is necessary for appropriate growth and development of the infant. The results of several studies have also suggested that the infant diet, especially a breastfeeding pattern with frequent consumption of fruits and vegetables, is positively associated with higher IQ in later years, improved cognitive development, and a lower risk of adiposity [64,65,66]. The described outcome may be a summary effect of the actions of various bioactive compounds present in breast milk, vegetables, and fruits, including plant miRNAs. Thus, to propose the potential influence of the five identified plant miRNAs on infants, miRNA target prediction and functional annotations were performed. The human targets selected by three out of five independent bioinformatics methods were further considered. This type of consensus prediction is widely used to reduce the number of false positive results [67,68]. For the five analyzed plant miRNAs, 385 unique putative human targets were proposed (Table S1). Among them, targets encoding proteins involved in regulation of localization, enzyme binding, Ras protein signal transduction and Rac guanyl-nucleotide exchange factor activity were significantly enriched (Figure S2). The Ras and Rac proteins are small GTPases belonging to the Ras superfamily that control various essential biochemical processes in a cell, such as cell proliferation and differentiation, survival, apoptosis, cytoskeleton organization, vesicular transport, protein sorting, and others. Ras molecules act as molecular switches, changing between an active (GTP-bound) and an inactive (GDP-bound) conformational state. Endogenous miRNAs identified in the animals’ breast milk exosomes are shown to affect immunity, inter alia. It was also proposed that the plant miRNAs analyzed in this study potentially influence the inflammatory responses and immune system. One of the predicted targets was the mRNA sequence of leukocyte-associated immunoglobulin-like receptor-1 (LAIR1) that is expressed by most immune cells. Cross-linking of this protein inhibits Natural Killer (NK) cell and T cell-mediated toxicity, as well as B cell-mediated signaling [69,70]. Another important immune-related proposed target is the CXC chemokine receptor type 2 (CXCR2) which is a key regulator of neutrophil recruitment and effector responses in a site of inflammation [71]. From the point of view of the infant health, targets encoding molecules involved in transport, signaling pathways, regulation of transcription, neuron development, apoptosis and metabolism are also essential (Table S2 and Figure S1). As mentioned in the Introduction, human miRNAs abundant in breast milk participate in various important biological processes including immune system development, cell differentiation, endo/exocytosis, metabolic processes, growth, and apoptosis [20,72]. In this manner, identified plant miRNAs may have synergistic or antagonistic roles in relation to these molecules, which altogether potentially result in a complex regulator system. Doubts have been immediately raised in regards to this type of analysis, and to its results concerning whether the identified plant miRNAs are in biologically active amounts that will enable them to effectively regulate the presented human targets. It was estimated that approximately 100–1000 copies of miRNA per cell should sufficiently affect gene expression [73,74]. However, it should be noted that the mechanism of gene expression regulation by endogenous miRNAs has been intensively investigated during the past years, and still new reports are appearing showing how target levels, their half-lives, processing, complementary, competing endogenous molecules, and other conditions affect the miRNAs effectiveness. Thus, it is very difficult to certainly state that a given amount of plant food-derived miRNA is sufficient or insufficient to influence human biological processes, especially when we recall that not all plant miRNAs present in food sources are able to pass through the GI tract and enter human circulation [52]. This phenomenon still needs to be deeply investigated.

To summarize, the present study confirms our earlier findings and shows that certain plant miRNAs are present in human breast milk, including exosome fractions. The concentrations of the identified molecules were rather low compared to the endogenous control, and varied significantly between the samples, which may reflect inter-individual properties. Additionally, no specific association was observed between the dietary pattern of the mothers from which the breast milk was collected and the profiles of the evaluated plant miRNAs. To avoid any further speculation, the appropriate controls were performed at different stages of the experiment and confirmed that the obtained results do not represent a false-positive effect. The potential functionality of the identified plant miRNAs was evaluated by bioinformatics analysis, which showed that the mentioned molecules may influence important biological processes in infants. Considering all the above-discussed results, the present study revealed novel properties of plant food-derived miRNAs, which may potentially be used further in developing natural medical solutions for treating known diseases in very small babies.

## 4. Materials and Methods

### 4.1. Ethics Statement

All the experiments were approved by the Local Bioethics Committee supervising the Medical University of Warsaw (KB/71/2016; 15 March 2016) and performed in compliance with the Declaration of Helsinki. Written informed consent was obtained from all the mothers prior to the study. All mothers were also asked to fill out a questionnaire concerning age, lactation stage, any diagnosed diseases, medications used, and their daily dietary pattern (e.g., vegetarian or non-vegetarian, but including plant food every day). Mothers were also asked to list all the plant food that they consumed up to 24 h before the milk sample collection (Table 2).

### 4.2. Milk Sample Collection

Human mature milk samples were aseptically collected from six mothers using an electric or manual breast pump, sterile bottles, and other accessories. All participating women and their infants were healthy at the time of milk collection. Mothers also did not take any medication/supplements at that time, and did not use any cosmetics/solutions/wraps for breasts up 24 h before sample collection. Depending on the sample volume (range, 25–65 mL), the milk was separated into 5 mL aliquots, directly transported to the laboratory on dry ice, and stored at −80 °C until further analysis. The collected milk samples differed in color (from very white to distinctly yellow) and in the lipid content, which was visible after centrifugation during the exosome isolation procedure.

### 4.3. Total RNA Extraction from Whole Milk and Exosome Fraction

Total RNA extraction was performed using a mirVana isolation kit (Ambion) with some modifications. After thawing the milk on ice, 2 mL of whole milk was diluted with 4 mL of Lysis/Binding solution. To ensure effective denaturation, the samples were well mixed by vortexing, and incubated for 5 min at room temperature. After lysis, 1.6 fmol syn-cel-miR-39 (synthetic miRNA from *C. elegans*, Qiagen N.V., Hilden, Germany) was added to each sample as a spike-in control. Next, 1/10 volumes of miRNA homogenate additive were added, mixed by vortexing for 30 s, and incubated on ice for 10 min. Then, an equal volume of acid/phenol/chloroform solution was added, mixed by vortexing for 1 min and centrifuged at 10,000× *g* for 20 min. After collecting the aqueous phase, the extraction step with acid/phenol/chloroform solution was repeated. Subsequent steps were processed according to the manufacturer’s protocol. At the end of the procedure, RNA was eluted in 100 μL of nuclease-free deionized water (EurX, Gdansk, Poland).

To isolate the total RNA from the exosome fraction, 3 mL of whole milk sample was centrifuged at 2000× *g* for 10 min to remove the fat globules. The supernatant was centrifuged further at 12,000× *g* for 30 min and filtered through a 0.22 μm MF-Millipore MCE membrane filter (Millipore, MA, USA) to eliminate the cells and cellular debris. Two mL of supernatant was mixed with 500 μL of the ExoQuick Exosome Precipitation Solution (SBI, Palo Alto, CA, USA) and incubated at 4 °C for 12 h. The ExoQuick/supernatant mixture was centrifuged at 1500× *g* for 30 min to obtain a beige exosome pellet. The RNA isolation from the exosome fraction was performed immediately by adding 1.2 mL of Lysis/Binding solution. After lysis, which occurred during pipetting, 1.6 fmol syn-cel-miR-39 was added to each sample as a spike-in control. Further isolation steps were performed according to the mirVana isolation kit manufacturer’s protocol, with repeated extraction steps with acid/phenol/chloroform solution. The concentration of the syn-cel-miR-39 recovered in both procedures was used for the calculations of the miRNA isolation efficiency in each sample.

### 4.4. RNA Analysis and Quantifications

The RNA concentration and purity (A260/280 ratio) were measured using the NanoDrop spectrophotometer (ND-1000, Thermo Scientific, Waltham, MA, USA). The miRNA concentration and miRNA/small RNA ratio were quantified by micro-capillary electrophoresis using the small RNA Chip kit (Agilent, Santa Clara, CA, USA) with the Agilent Bioanalyzer 2100 instrument (Agilent, Santa Clara, CA, USA). 

### 4.5. Reverse Transcription

Reverse transcription reactions were performed using the TaqMan^®^ miRNA reverse transcription kit (Applied Biosystems, Foster City, CA, USA) with TaqMan^®^ specific stem-loop primers (miR168a, assay ID 007594_mat; miR156a, assay ID 000333; miR166a, assay ID 000347; miR172a, assay ID 000359; miR167a, assay ID 000348; hsa-miR-148a-3p, assay ID 000470; cel-miR-39, assay ID 000200; Applied Biosystems) in a volume of 15 μL according to the manufacturer’s protocol. In each reaction, 1 μL of total RNA was used. RT was carried out in a thermal cycler (Labcycler SensoQuest, Syngen, Germany) with the following programme: 16 °C for 30 min, 42 °C for 30 min, and 85 °C for 5 min, and held at 4 °C. RT products were stored at −20 °C prior to real-time PCR.

A standard curve was generated using chemically synthesized miR172a molecule (42.2 μM, sequence: AGAAUCUUGAUGAUGCUGCAU), that was serially diluted (in range of 82–0.005 pM) and added to the RT reaction mixtures at a volume of 1 μL per reaction.

### 4.6. Quantitative PCR Analysis

The qRT-PCR reactions were performed with TaqMan^®^ Universal PCR Master Mix (no AmpErase^®^ UNG kit; Applied Biosystems), specific miRNA primers and hydrolysis probes (probe identifiers as above, Applied Biosystems). Each reaction was scaled down to a total volume of 10 μL containing 0.67 μL of RT product and was performed in triplicate. In each run of qRT-PCR, no-template controls were performed. The qRT-PCR reactions were carried out using the Mic thermocycler (Bio Molecular Systems, Queensland, Australia) at the following conditions: 95 °C for 2 min, followed by 40 cycles of 95 °C for 15 s and 60 °C for 1 min. The raw data was analyzed using the automatic quantification cycle setting (Cq) for assigning the baseline and detection threshold.

Absolute concentrations of the miRNAs were calculated based on the standard curve obtained from serial dilutions of synthetic miR172a reverse transcribed and amplified in real-time reaction. The detection limit of the miRNA assay was designated as the highest Cq value (35.2) within the linear range of the generated standard curve (Figure S3). Results with a Cq value >35.5 were excluded from further analysis. The Cq value of the syn-cel-miR-39 was used to normalize the quantity of plant miRNAs in each sample and to compare the expression levels between the milk samples (Delta *C*_t_ method).

### 4.7. Prediction and Annotation of Putative Human Target Genes for Identified Plant miRNAs

To propose the potential influence of the identified food-derived molecules on the infants, the putative human targets for the 5 plant miRNAs were predicted and annotated. In the first step of this analysis, all available human 3′ UTR, 5′ UTR and coding sequences (CDS) were collected from the Ensembl Genes database (December 2016, 87 release). Currently, there is no “golden standard” for plant miRNA target prediction in a cross-kingdom manner, therefore target prediction was performed using the tools4miRs meta-server (www.tools4mirs.org) [75], with 5 different target prediction algorithms incorporated into the analysis: miRanda, PITA, Rna22, GUUGle and miRmap. Moreover, for each programme, restrictive parameters were set up, specifically: (1) for Rna22: no G:U wobbles were allowed in the “seed” region, −19 Kcal/mol was the maximum folding energy of the heteroduplex; sensitivity was 63%, specificity was 61%, no mismatches were allowed in the “seed” region, 12 was the minimum number of paired-up bases in the heteroduplex, 7 was the length of the “seed” region; (2) for miRmap: 6 or 7 was the length of the “seed” region, no mismatches were allowed in the “seed” region and no G:U wobbles were allowed in the “seed” region; (3) for miRanda: 160 was the minimum score, and −19 Kcal/mol was the maximum folding energy of the duplex structure; (4) for PITA: −19 was the maximum ΔΔG score; and (5) for GUUGle: 12 was the minimum number of matches in the duplex. From the results generated by tools4miRs, the unique target:miRNA pairs were collected if the same exact miRNA binding site was predicted by at least 3 out of the 5 prediction algorithms. For further annotation analyses, the Associated Gene Names and protein sequences encoded by the gathered human mRNA targets were collected and used. The GO annotation was performed using Blast2GO software (www.blast2go.com), in which the GO terms were obtained as a result of three steps: (1) Blastp search against the “nr” NCBI database with the “mammals” taxon restriction and E-value threshold of 1e^−10^, (2) mapping Blast hits on the GO terms and (3) filtering the annotations with an E-value threshold of 1e^−10^. For the KEGG pathway mapping, the online service KAAS (KEGG Automatic Annotation Server; http://www.genome.jp/tools/kaas/) [76] was used, which performed Blastn comparison against the manually validated set of human genes in the KEGG GENES database. The GO enrichment was done with the use of the Ontologizer program [77] and the predicted miRNA targets served as a study set. In turn, all the available human sequences collected from the Ensemble Gene database served as the population set. The “Term-For-Term” algorithm with Benjamini-Hochberg correction for multiple testing and a *p*-value threshold set at 0.05 was chosen for the calculations. 

### 4.8. Statistical Analysis

Data shown are presented as the mean ± SD of the quantitative PCR reaction triplicates. The principal component analysis (PCA) was done with the use of the “stats” package in R, and the calculations were based on the relative quantification data obtained for each of the 5 identified plant miRNAs in whole breast milk samples. The comparison of the relative quantification levels calculated for the 5 plant miRNAs identified in the whole milk and exosome samples from vegetarians/non-vegetarians was performed using the unpaired *t*-test. Differences were considered statistically significant at a *p*-value < 0.05. 

## Figures and Tables

**Table 1:** RNA concentration obtained from whole milk and the exosomes fraction of the breast milk samples. Concentration of total RNA was measured on a NanoDrop spectrophotometer. The concentration of microRNA molecules (miRNAs) and the miRNA/small RNA ratio was measured with the Agilent Bioanalyzer 2100 instrument. Isolation efficiency is presented as the % of syn-cel-miR-39 recovered during the isolation procedure (calculated using data from qRT-PCR analysis for syn-cel-miR-39 in each sample).

Sample		Total RNA Concentration (ng/μL)	miRNA Concentration (ng/μL)	miRNA/Small RNA Ratio (%)	Isolation Efficiency (%)
**Whole milk**	R1	1018	51.3	43	9
	R2	120	20.35	43.5	18.5
	R3	94	15.15	40	13.4
	R4	407	41	40.5	11.9
	R5	134	20.25	43	18.8
	R6	111	13.7	45	14.4
**Exosomes**	R1	228	37.19	19.5	9.95
	R2	40	18	33.5	17.8
	R3	42 *	15.45	29.5	17.7
	R4	143	15.35	27	13.9
	R5	21 *	10.85	42.5	18.3
	R6	23	9.75	45	27.15

* samples with A260/280 ratio < 1.8.

**Table 2:** Information regarding participant age, lactation stage, dietary pattern and plant food consumed up to 24 h before milk sample collection. All presented information was collected from the questionnaire obtained from the mothers after they signed informed consent.

Mothers’ ID	R6	R1	R2	R4	R5	R3
Age	32	34	35	32	29	28
Lactation stage (months)	1.5	3.5	7	2.5	5	8
Diet	vegetarian	vegetarian	vegetarian	plants included every day	plants included every day	plants included every day
Plants in diet up to 24 h before milk collection	tomato, pepper, cucumber, banana, pomegranate, oregano, basil, thyme, berry, barley, oat, wheat, rye	strawberries, spinach, tomato, cucumber, carrot, potato, zucchini, orange, salad, olives, mint, coriander, parsley, sesame, pumpkin seeds, sunflowers seeds, raisins, cranberry, oat, rye, millet, wheat	strawberries, tomato cucumber, carrot, salad, wheat, leek	strawberries, tomato, carrot, apple, corn, wheat, rye, kiwi	tomato, pepper, onion, cucumber, potato, radish, chive, salad, zucchini, banana, coriander, bilberry, wheat, rye	tomato, carrot, cauliflower, broccoli, plump, pear, potato, dill, rice, barley, corn, oat, rye

## Supplementary Materials

Supplementary materials can be found at www.mdpi.com/1422-0067/19/1/37/s1.
