# Supplementary material for: Detection of Plant miRNAs Abundance in Human Breast Milk
Source: Int J Mol Sci. 2017 Dec 23;19(1):37. doi: 10.3390/ijms19010037 (PMC5795987; doi:10.3390/ijms19010037)
Supplement: Supplementary file 1 [file ijms-19-00037-s001.pdf]

SUPPLEMENTARY INFORMATION

Detection of plant miRNAs abundance in human breast milk

Anna Lukasik[1,+], Iwona Brzozowska[1,+], Urszula Zielenkiewicz[1] and Piotr Zielenkiewicz[1,2,*]

[1]Institute of Biochemistry and Biophysics, Polish Academy of Sciences, Warsaw, 02-106, Poland
[2]Department of Plant Molecular Biology, Institute of Experimental Plant Biology and Biotechnology, University of Warsaw, Warsaw, 02-096, Poland
[*]piotr@ibb.waw.pl
[+]These authors contributed equally to this work

SUPPLEMENTARY FIGURES

Supplementary Figure S1. Results of the GO term annotation for plant miRNAs targets. The GO terms ("Biological Process" category) annotation was performed by the Blast2GO software. The human targets predicted for 5 evaluated plant miRNAs were used. Figure represents the pie chart graph of certain GO terms distribution – numbers in brackets represent number of annotated human targets.

Supplementary Figure S2. Results of the GO term enrichment analysis for plant miRNA targets. The GO terms annotation was performed by the Blast2GO software. The human targets predicted for 5 evaluated plant miRNAs were used. Figure represent hierarchical tree graph of enriched GO terms in the "Biological Process" and "Molecular Function" categories. The darker the colour, the higher GO term enrichment. The arrow colours indicate type of relationship between the GO terms, with black signifying "is a", blue signifying "part of" and green signifying "regulates".

Figure S1.

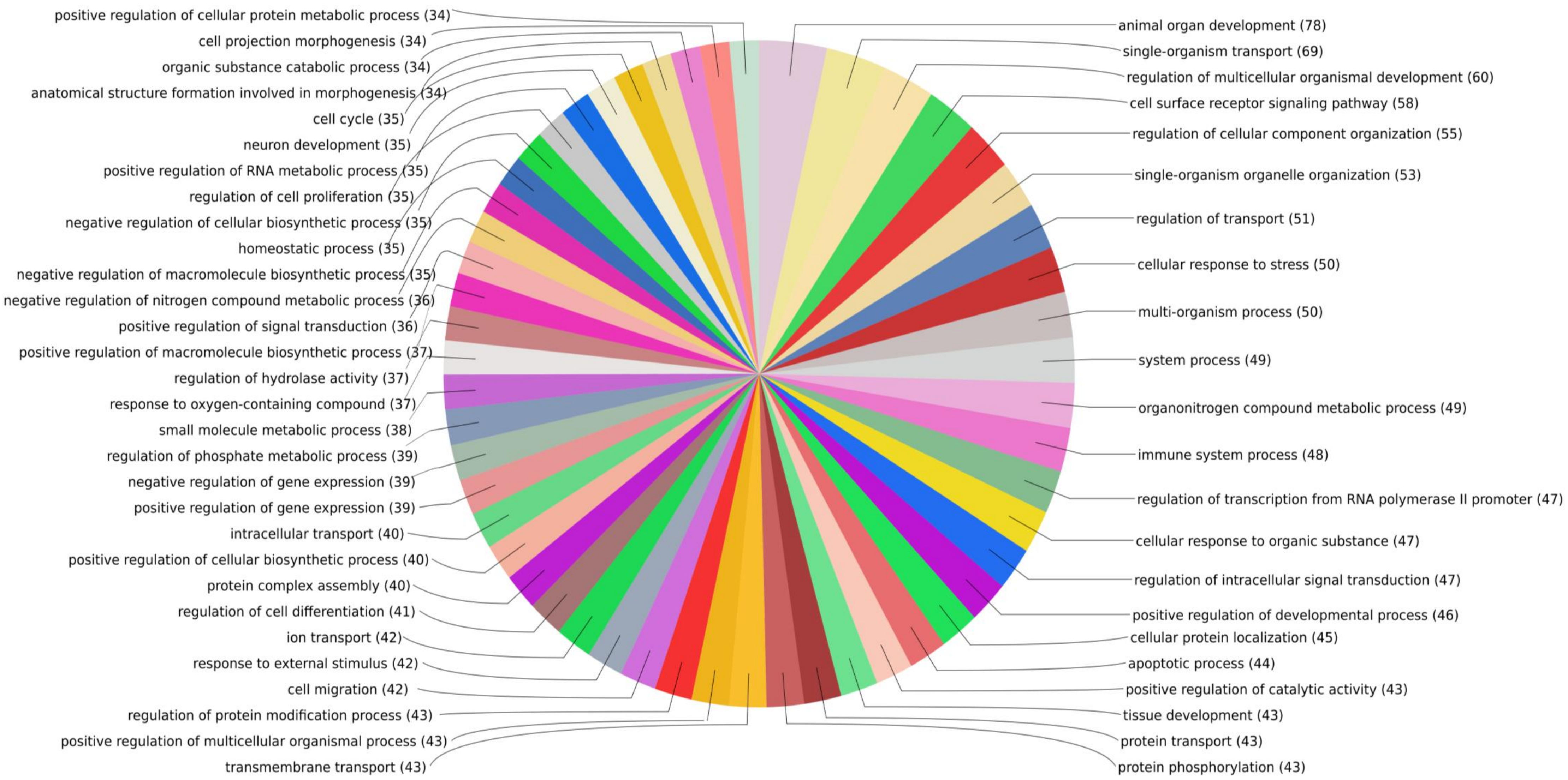
positive regulation of cellular protein metabolic process (34)
cell projection morphogenesis (34)
organic substance catabolic process (34)
anatomical structure formation involved in morphogenesis (34)
cell cycle (35)
neuron development (35)
positive regulation of RNA metabolic process (35)
regulation of cell proliferation (35)
negative regulation of cellular biosynthetic process (35)
homeostatic process (35)
negative regulation of macromolecule biosynthetic process (35)
negative regulation of nitrogen compound metabolic process (36)
positive regulation of signal transduction (36)
positive regulation of macromolecule biosynthetic process (37)
regulation of hydrolase activity (37)
response to oxygen-containing compound (37)
small molecule metabolic process (38)
regulation of phosphate metabolic process (39)
negative regulation of gene expression (39)
positive regulation of gene expression (39)
intracellular transport (40)
positive regulation of cellular biosynthetic process (40)
protein complex assembly (40)
regulation of cell differentiation (41)
ion transport (42)
response to external stimulus (42)
cell migration (42)
regulation of protein modification process (43)
positive regulation of multicellular organismal process (43)
transmembrane transport (43)
animal organ development (78)
single-organism transport (69)
regulation of multicellular organismal development (60)
cell surface receptor signaling pathway (58)
regulation of cellular component organization (55)
single-organism organelle organization (53)
regulation of transport (51)
cellular response to stress (50)
multi-organism process (50)
system process (49)
organonitrogen compound metabolic process (49)
immune system process (48)
regulation of transcription from RNA polymerase II promoter (47)
cellular response to organic substance (47)
regulation of intracellular signal transduction (47)
positive regulation of developmental process (46)
cellular protein localization (45)
apoptotic process (44)
positive regulation of catalytic activity (43)
tissue development (43)
protein transport (43)
protein phosphorylation (43)

Figure S2.

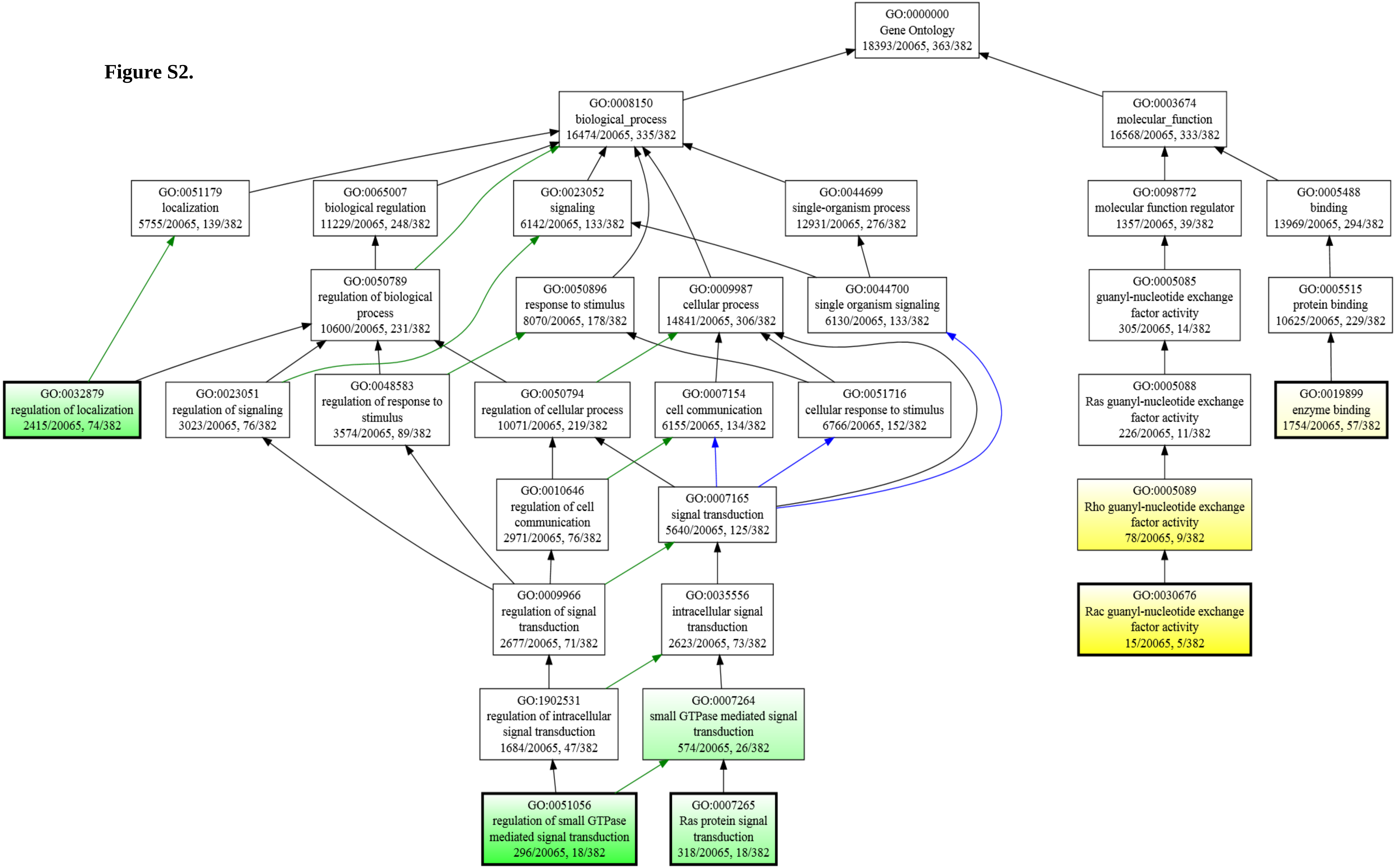
GO:0000000
Gene Ontology
18393/20065, 363/382
GO:0008150
biological_process
16474/20065, 335/382
GO:0003674
molecular_function
16568/20065, 333/382
GO:0051179
localization
5755/20065, 139/382
GO:0065007
biological regulation
11229/20065, 248/382
GO:0023052
signaling
6142/20065, 133/382
GO:0044699
single-organism process
12931/20065, 276/382
GO:0098772
molecular function regulator
1357/20065, 39/382
GO:0005488
binding
13969/20065, 294/382
GO:0050789
regulation of biological process
10600/20065, 231/382
GO:0050896
response to stimulus
8070/20065, 178/382
GO:0009987
cellular process
14841/20065, 306/382
GO:0044700
single organism signaling
6130/20065, 133/382
GO:0005085
guanyl-nucleotide exchange factor activity
305/20065, 14/382
GO:0005515
protein binding
10625/20065, 229/382
GO:0032879
regulation of localization
2415/20065, 74/382
GO:0023051
regulation of signaling
3023/20065, 76/382
GO:0048583
regulation of response to stimulus
3574/20065, 89/382
GO:0050794
regulation of cellular process
10071/20065, 219/382
GO:0007154
cell communication
6155/20065, 134/382
GO:0051716
cellular response to stimulus
6766/20065, 152/382
GO:0005088
Ras guanyl-nucleotide exchange factor activity
226/20065, 11/382
GO:0019899
enzyme binding
1754/20065, 57/382
GO:0010646
regulation of cell communication
2971/20065, 76/382
GO:0007165
signal transduction
5640/20065, 125/382
GO:0005089
Rho guanyl-nucleotide exchange factor activity
78/20065, 9/382
GO:0009966
regulation of signal transduction
2677/20065, 71/382
GO:0035556
intracellular signal transduction
2623/20065, 73/382
GO:0030676
Rac guanyl-nucleotide exchange factor activity
15/20065, 5/382
GO:1902531
regulation of intracellular signal transduction
1684/20065, 47/382
GO:0007264
small GTPase mediated signal transduction
574/20065, 26/382
GO:0051056
regulation of small GTPase mediated signal transduction
296/20065, 18/382
GO:0007265
Ras protein signal transduction
318/20065, 18/382

a

Mic Bio Molecular Systems

standard	conc [pM]	log_2 [conc]	mean Cq
S1	82	6,358	18,90
S3	5,125	2,358	23,58
S4	1,28	0,356	26,04
S6	0,08	-3,644	30,15
S7	0,02	-5,644	32,43
S8	0,005	-7,644	35,21
S10	0,0003125	-11,644	37,42
S11	0,000078	-13,646	0,00

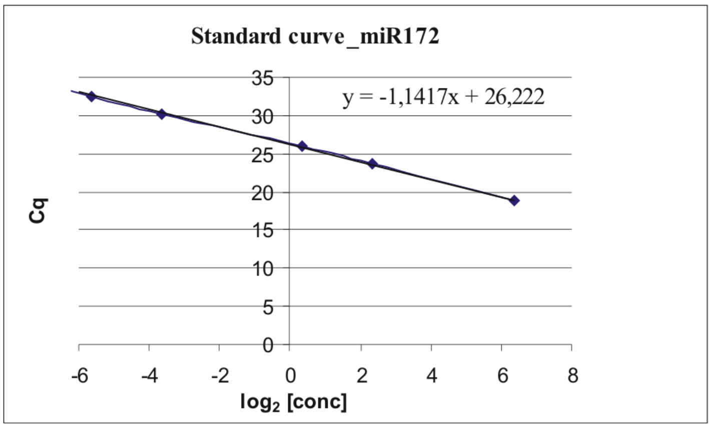

b

LightCycler 480 Roche

standard	conc [pM]	log_2 [conc]	mean Cq
S1	82	6,358	20,69
S3	5,125	2,358	25,28
S4	1,28	0,356	27,85
S6	0,08	-3,644	31,77
S7	0,02	-5,644	34,78
S8	0,005	-5,644	35,00
S10	0,0003125	-5,644	35,00
S11	0,000078	-5,644	35,00

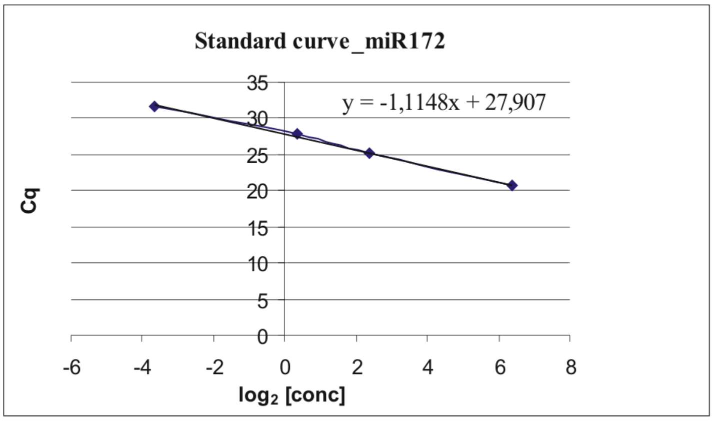

Supplementary Figure S3. Standard curves generated in qRT-PCR analysis for synthetic miR172a molecule using Mic instrument (a) and LightCycler480 (b). Values marked in red were excluded from the analysis.

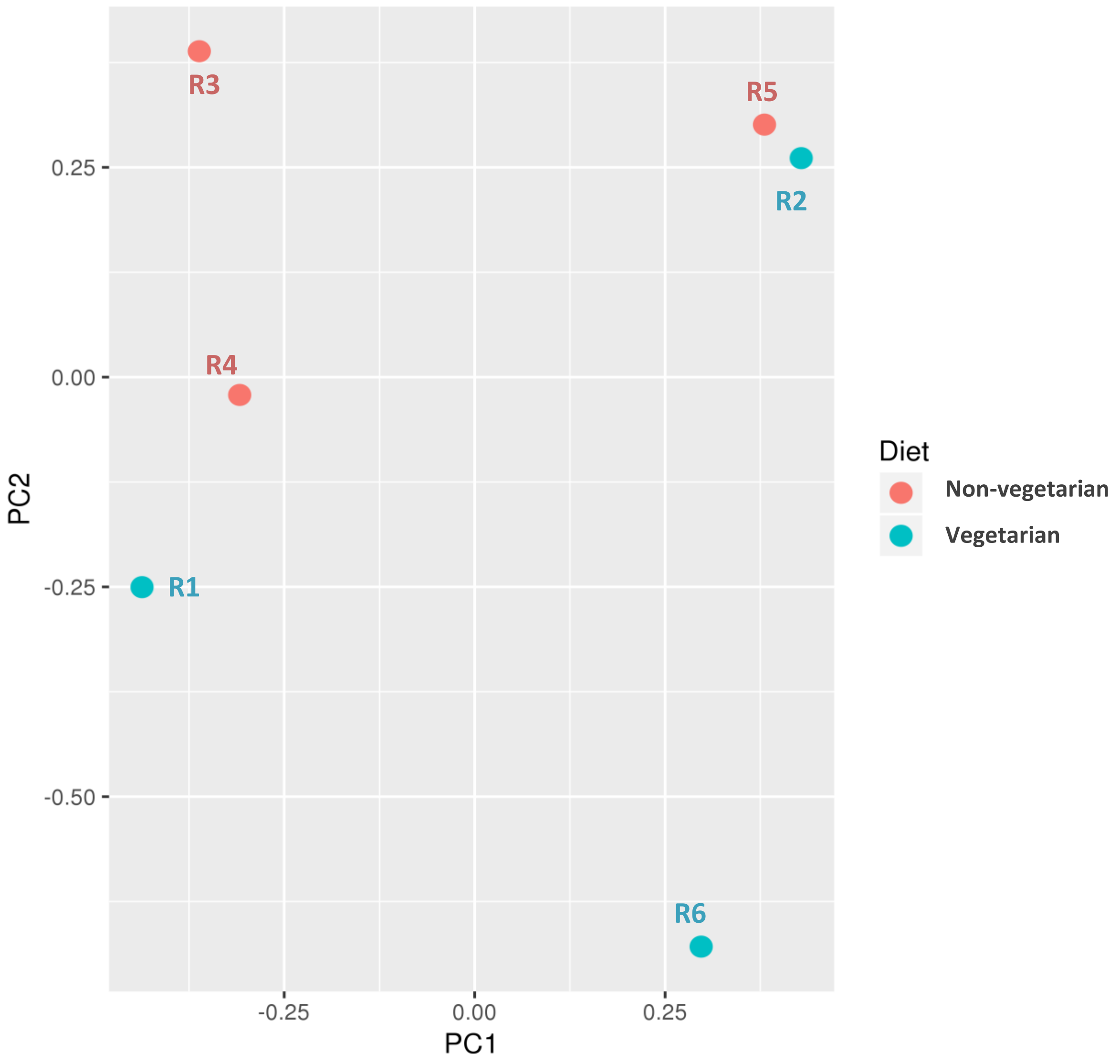

Supplementary Figure S4. Clustering of the individual human breast milk samples. The Principal Component Analysis (PCA) was performed based on relative quantification data of 5 plant miRNAs, which levels were measured by qRT-PCR in the whole breast milk samples collected from healthy human volunteers.

SUPPLEMENTARY TABLES

Supplementary Table S1. Results of the target prediction for evaluated plant miRNAs. The targets prediction for 5 plant miRNAs was performed with the use of tools4miRs service which incorporated into analysis five different target prediction algorithms. As an initial target set all collected human 3'UTR, 5'UTR and coding sequences were used.

Target mRNA	miRNA	binding site	GUUGle (length of miRNA:mRNA match)	miRanda (binding free energy)	miRmap (ΔΔG)	PITA (binding free energy)	RNA22 (binding free energy)	#tools
3utr|ENST00000005995|PRSS21	ath-miR156a-5p	43:62	13	-23,65			-19,00	3
3utr|ENST00000157600|LMCD1	ath-miR156a-5p	6430:6451	12	-25,52			-21,60	3
3utr|ENST00000216133|CBX7	ath-miR167a-5p	2049:2070	12	-28,52		-28,60		3
3utr|ENST00000216187|FOXRED2	ath-miR156a-5p	793:812		-20,20	-19,20	-22,16		3
3utr|ENST00000219478|ZNF500	ath-miR166a-3p	69:81	12		-25,40	-25,10		3
3utr|ENST00000220509|VPS18	ath-miR156a-5p	289:307		-21,82	-20,50	-21,20		3
3utr|ENST00000220597|PAG1	ath-miR156a-5p	643:662		-20,29	-19,60	-19,20		3
3utr|ENST00000223368|BCL7B	ath-miR167a-5p	310:329	13	-27,06		-26,70		3
3utr|ENST00000231368|LNPEP	ath-miR156a-5p	5039:5059		-20,21	-18,60		-19,30	3
3utr|ENST00000231656|CDX1	ath-miR166a-3p	72:86	14		-26,60	-29,70		3
3utr|ENST00000233202|SLC11A1	ath-miR166a-3p	824:836	12		-21,00	-20,70		3
3utr|ENST00000238477|ALG2	ath-miR156a-5p	1271:1291		-20,28	-19,00	-19,30		3
3utr|ENST00000238789|ATAD2B	ath-miR156a-5p	928:941	13		-20,00	-20,40		3
3utr|ENST00000242108|EEPD1	ath-miR167a-5p	634:657		-25,55	-24,60	-21,90		3
3utr|ENST00000248633|PEX1	ath-miR167a-5p	374:395		-19,79	-21,50	-19,50		3
3utr|ENST00000249786|SERF2	ath-miR156a-5p	682:695	13		-26,80	-27,20		3
3utr|ENST00000251038|ZC3H14	ath-miR156a-5p	14282:14300		-22,09	-21,50	-21,60		3
3utr|ENST00000252660|MAS1	ath-miR156a-5p	1274:1293		-21,02	-20,60	-21,70		3

3utr|ENST00000254963|HSPA12B	ath-miR166a-3p	183:204		-29,04	-27,60	-27,60		3
3utr|ENST00000255078|IGHMBP2	ath-miR156a-5p	488:507		-23,26	-22,50	-22,80		3
3utr|ENST00000256031|ATP13A3	ath-miR156a-5p	2029:2049		-20,92	-20,90	-21,30		3
3utr|ENST00000256186|MICALCL	ath-miR167a-5p	142:163		-21,77	-22,50	-19,40		3
3utr|ENST00000259021|KAT7	ath-miR156a-5p	4931:4943	12		-18,30	-20,99		3
3utr|ENST00000259050|MARCH7	ath-miR156a-5p	1043:1056	13		-17,70	-20,21		3
3utr|ENST00000259392|SLC31A2	ath-miR156a-5p	436:454		-21,99	-21,00	-20,70		3
3utr|ENST00000260276|C11orf1	ath-miR156a-5p	645:664		-19,35	-16,50	-20,00		3
3utr|ENST00000260327|CTDSPL2	ath-miR156a-5p	1954:1973		-21,31	-19,90	-20,60		3
3utr|ENST00000260364|NOX5	ath-miR166a-3p	2606:2626		-26,97	-22,80	-23,30		3
3utr|ENST00000260662|CENPO	ath-miR156a-5p	1076:1096		-19,75	-19,00	-22,40		3
3utr|ENST00000261263|RAB21	ath-miR156a-5p	1009:1030		-20,47	-20,00	-20,30		3
3utr|ENST00000261381|XYLT1	ath-miR156a-5p	6427:6442	15		-23,30	-23,40		3
3utr|ENST00000262238|YY1	ath-miR156a-5p	2356:2374		-23,19	-23,30	-22,90		3
3utr|ENST00000262502|SLC12A3	ath-miR156a-5p	598:620		-22,87	-16,40	-22,40		3
3utr|ENST00000262710|ACIN1	ath-miR156a-5p	429:448		-21,64	-22,40	-22,30		3
3utr|ENST00000263095|ZNF264	ath-miR156a-5p	6440:6458		-20,07	-20,40	-20,30		3
3utr|ENST00000264344|FAM13A	ath-miR156a-5p	1581:1595	14		-26,20	-26,80		3
3utr|ENST00000264607|ASB1	ath-miR156a-5p	793:812		-21,93	-21,30	-21,90		3
3utr|ENST00000264658|FBXL20	ath-miR156a-5p	7032:7045	13		-20,00	-21,43		3
3utr|ENST00000266544|NDUFA9	ath-miR156a-5p	2083:2101		-22,44	-20,50	-20,80		3
3utr|ENST00000267890|TTBK2	ath-miR156a-5p	4139:4157		-21,87	-19,10	-19,10		3
3utr|ENST00000271620|PRUNE	ath-miR156a-5p	624:642		-22,44	-22,40	-22,40		3
3utr|ENST00000272647|AMMECR1L	ath-miR156a-5p	1562:1577	15		-21,40	-21,50		3
3utr|ENST00000273853|CENPC	ath-miR156a-5p	2426:2445		-24,57	-22,70	-23,50		3
3utr|ENST00000274773|TRIM7	ath-miR166a-3p	57:77		-25,25	-24,20	-29,97		3
3utr|ENST00000279024|KIAA1755	ath-miR156a-5p	1401:1420		-20,79	-19,80	-19,50		3
3utr|ENST00000280241|DLG2	ath-miR156a-5p	3285:3305		-22,94	-20,90	-20,90		3

3utr|ENST00000281938|HSPB8	ath-miR156a-5p	657:669	12		-18,30	-19,30		3
3utr|ENST00000283426|PLEKHG4B	ath-miR156a-5p	3910:3932		-21,05	-18,80	-19,10		3
3utr|ENST00000283946|FBXO36	ath-miR156a-5p	54:73		-20,15	-21,10	-23,60		3
3utr|ENST00000285397|AK9	ath-miR156a-5p	277:298		-24,68	-23,80	-23,80		3
3utr|ENST00000285599|MAN2B2	ath-miR156a-5p	224:244		-24,07	-21,60	-22,30		3
3utr|ENST00000287675|EXOG	ath-miR156a-5p	684:707		-25,77	-20,10	-24,30		3
3utr|ENST00000288050|PDPR	ath-miR156a-5p	3022:3042		-19,28	-16,70	-20,93		3
3utr|ENST00000297323|ADCY1	ath-miR166a-3p	3909:3922	13		-25,00	-24,80		3
3utr|ENST00000300176|AGFG2	ath-miR156a-5p	2009:2030		-23,51	-21,70	-22,10		3
3utr|ENST00000302475|MCC	ath-miR156a-5p	2214:2233		-20,55	-20,50	-19,60		3
3utr|ENST00000302506|CDC25A	ath-miR156a-5p	178:197		-24,99	-24,30	-24,60		3
3utr|ENST00000302824|STARD5	ath-miR156a-5p	3523:3541		-23,11	-20,50	-21,10		3
3utr|ENST00000302851|ZNF561	ath-miR156a-5p	605:625		-23,20	-18,80	-20,00		3
3utr|ENST00000303077|SIX2	ath-miR156a-5p	419:441		-24,27	-19,50	-23,70		3
3utr|ENST00000303391|MECP2	ath-miR156a-5p	5657:5675		-22,75	-22,90	-22,40		3
3utr|ENST00000304987|SIK2	ath-miR156a-5p	3026:3046		-23,00	-20,60	-21,00		3
3utr|ENST00000308910|GCSAM	ath-miR156a-5p	305:323		-25,04	-22,80	-24,90		3
3utr|ENST00000309859|GGA2	ath-miR156a-5p	1064:1083		-21,67	-19,80	-19,40		3
3utr|ENST00000311565|PPARD	ath-miR156a-5p	469:487		-20,20	-19,70	-20,80		3
3utr|ENST00000311601|SH3PXD2B	ath-miR156a-5p	4284:4303		-26,41	-25,50	-26,50		3
3utr|ENST00000315599|CD209	ath-miR156a-5p	2211:2223	12		-18,70	-19,20		3
3utr|ENST00000315792|C8orf4	ath-miR156a-5p	105:118	13		-21,40	-22,50		3
3utr|ENST00000317802|TSPYL6	ath-miR156a-5p	941:960		-22,61	-22,40	-22,50		3
3utr|ENST00000318282|MKL2	ath-miR156a-5p	1113:1126	13		-22,70	-23,30		3
3utr|ENST00000319033|ALG2	ath-miR156a-5p	278:298		-20,28	-19,00	-19,30		3
3utr|ENST00000324262|CNDP2	ath-miR156a-5p	807:826		-20,30	-21,40	-20,30		3
3utr|ENST00000324301|CNDP2	ath-miR156a-5p	807:826		-20,30	-21,40	-20,30		3
3utr|ENST00000324685|RNF40	ath-miR166a-3p	1329:1352		-29,60	-20,00	-25,80		3

3utr|ENST00000324727|SCN4B	ath-miR156a-5p	1997:2014		-24,80	-24,10	-24,40		3
3utr|ENST00000325134|ZNF550	ath-miR156a-5p	2596:2615		-22,13	-20,80	-21,00		3
3utr|ENST00000328554|C22orf29	ath-miR156a-5p	469:490		-19,38	-19,40	-19,70		3
3utr|ENST00000329203|FAM181B	ath-miR156a-5p	586:600	14		-19,30	-24,81		3
3utr|ENST00000330276|SV2B	ath-miR156a-5p	1791:1811		-29,60	-26,60	-27,00		3
3utr|ENST00000330634|INF2	ath-miR156a-5p	1427:1448		-19,88	-20,60	-21,20		3
3utr|ENST00000331194|CCDC88C	ath-miR156a-5p	1019:1035	16		-27,60	-28,00		3
3utr|ENST00000332129|KIF21B	ath-miR166a-3p	2268:2288	16	-30,73		-33,24		3
3utr|ENST00000332411|KRT76	ath-miR156a-5p	461:480		-21,39	-20,80	-20,50		3
3utr|ENST00000333209|GPRIN3	ath-miR156a-5p	2009:2029		-23,22	-19,20	-20,10		3
3utr|ENST00000334218|CDC42BPA	ath-miR156a-5p	2167:2187		-22,09	-21,60	-22,50		3
3utr|ENST00000334801|BCL9L	ath-miR156a-5p	458:478		-19,98	-20,50	-21,00		3
3utr|ENST00000335185|CCDC73	ath-miR156a-5p	136:156		-22,90	-22,00	-22,60		3
3utr|ENST00000335251|INTU	ath-miR156a-5p	5136:5154		-20,69	-20,10	-19,90		3
3utr|ENST00000337990|ZC4H2	ath-miR156a-5p	576:596		-20,60	-21,10	-21,30		3
3utr|ENST00000338631|ACIN1	ath-miR156a-5p	429:448		-21,64	-22,40	-22,30		3
3utr|ENST00000339854|MEFV	ath-miR156a-5p	348:368		-24,83	-24,20	-24,60		3
3utr|ENST00000342665|SOX12	ath-miR166a-3p	2268:2281	13		-22,40	-24,79		3
3utr|ENST00000343003|SPATA13	ath-miR156a-5p	485:504	15	-26,45		-26,00		3
3utr|ENST00000343110|PRELP	ath-miR156a-5p	2633:2652		-19,74	-19,10	-21,20		3
3utr|ENST00000343218|DPYSL3	ath-miR156a-5p	909:928		-22,18	-20,20	-20,90		3
3utr|ENST00000343968|MS4A4A	ath-miR166a-3p	320:333	13		-25,20	-25,82		3
3utr|ENST00000345496|UBE2G2	ath-miR156a-5p	2476:2496		-20,83	-17,60	-20,67		3
3utr|ENST00000348831|ADARB1	ath-miR156a-5p	2064:2082		-21,45	-19,30	-19,70		3
3utr|ENST00000349139|WDR3	ath-miR156a-5p	5058:5077		-22,13	-17,90	-19,60		3
3utr|ENST00000349384|RREB1	ath-miR156a-5p	1013:1026	13		-19,80	-22,35		3
3utr|ENST00000351231|CDC25A	ath-miR156a-5p	178:197		-24,99	-24,30	-24,60		3
3utr|ENST00000351989|DGCR8	ath-miR156a-5p	1394:1413		-19,82	-19,10	-20,50		3

3utr|ENST00000354200|TBC1D20	ath-miR156a-5p	2300:2318		-25,38	-22,70	-23,30		3
3utr|ENST00000355209|POLR3H	ath-miR156a-5p	3393:3405	12		-22,70	-22,50		3
3utr|ENST00000355327|THSD4	ath-miR156a-5p	2789:2802	13		-22,60	-22,70		3
3utr|ENST00000356003|WWP2	ath-miR156a-5p	1510:1529		-21,15	-22,40	-21,90		3
3utr|ENST00000357260|FAM212B	ath-miR156a-5p	4169:4187		-21,01	-21,00	-23,06		3
3utr|ENST00000357481|ACIN1	ath-miR156a-5p	429:448		-21,64	-22,40	-22,30		3
3utr|ENST00000357947|TLL2	ath-miR156a-5p	193:212		-19,38	-18,80	-19,00		3
3utr|ENST00000358246|MS4A7	ath-miR156a-5p	1064:1079	15		-19,30	-21,10		3
3utr|ENST00000359062|PDE3A	ath-miR156a-5p	4012:4031		-22,43	-18,80	-21,30		3
3utr|ENST00000359154|WWP2	ath-miR156a-5p	1510:1529		-21,15	-22,40	-21,90		3
3utr|ENST00000360694|PPARD	ath-miR156a-5p	469:487		-20,20	-19,70	-20,80		3
3utr|ENST00000360697|ADARB1	ath-miR156a-5p	2064:2082		-21,45	-19,30	-19,70		3
3utr|ENST00000360835|SCUBE1	ath-miR166a-3p	21:39		-33,80	-31,00	-31,10		3
3utr|ENST00000361642|ZEB1	ath-miR156a-5p	2227:2246		-22,90	-20,40	-21,40		3
3utr|ENST00000366764|CDC42BPA	ath-miR156a-5p	2167:2187		-22,09	-21,60	-22,50		3
3utr|ENST00000366766|CDC42BPA	ath-miR156a-5p	2167:2187		-22,09	-21,60	-22,50		3
3utr|ENST00000366767|CDC42BPA	ath-miR156a-5p	2167:2187		-22,09	-21,60	-22,50		3
3utr|ENST00000366769|CDC42BPA	ath-miR156a-5p	2167:2187		-22,09	-21,60	-22,50		3
3utr|ENST00000366847|FGFR1OP	ath-miR156a-5p	6604:6622		-20,17	-20,10	-20,10		3
3utr|ENST00000366898|PARK2	ath-miR156a-5p	1916:1935		-22,27	-22,00	-21,80		3
3utr|ENST00000367080|PFKFB2	ath-miR156a-5p	1321:1341		-22,80	-18,50	-19,45		3
3utr|ENST00000367467|SASH1	ath-miR156a-5p	363:382		-22,86	-22,60	-23,20		3
3utr|ENST00000367615|NPHS2	ath-miR156a-5p	405:426		-22,22	-19,90	-19,90		3
3utr|ENST00000367616|NPHS2	ath-miR156a-5p	405:426		-22,22	-19,90	-19,90		3
3utr|ENST00000368614|LRRC27	ath-miR156a-5p	3319:3331	12		-22,60	-23,82		3
3utr|ENST00000368614|LRRC27	ath-miR156a-5p	1020:1039		-20,64	-20,00	-20,50		3
3utr|ENST00000368934|PRUNE	ath-miR156a-5p	624:642		-22,44	-22,40	-22,40		3
3utr|ENST00000368935|PRUNE	ath-miR156a-5p	624:642		-22,44	-22,40	-22,40		3

3utr|ENST00000368936|PRUNE	ath-miR156a-5p	624:642		-22,44	-22,40	-22,40		3
3utr|ENST00000368937|PRUNE	ath-miR156a-5p	624:642		-22,44	-22,40	-22,40		3
3utr|ENST00000369183|FAM204A	ath-miR156a-5p	1351:1369		-29,46	-26,90	-27,50		3
3utr|ENST00000369423|IL9R	ath-miR156a-5p	509:528		-20,29	-20,10	-20,40		3
3utr|ENST00000370328|GABRE	ath-miR156a-5p	1291:1305	14		-21,60	-25,50		3
3utr|ENST00000370958|LRRC7	ath-miR156a-5p	1433:1452		-21,32	-18,90	-21,20		3
3utr|ENST00000371873|CMPK1	ath-miR156a-5p	1201:1214	13		-18,10	-20,80		3
3utr|ENST00000373471|FNDC5	ath-miR156a-5p	426:448		-21,93	-16,80	-22,10		3
3utr|ENST00000373504|CHIC1	ath-miR156a-5p	6005:6026		-23,86	-20,60	-20,70		3
3utr|ENST00000373652|FBXO36	ath-miR156a-5p	54:73		-20,15	-21,10	-23,60		3
3utr|ENST00000373789|RTKN2	ath-miR156a-5p	2403:2423		-20,24	-19,70	-19,60		3
3utr|ENST00000374220|SLC31A2	ath-miR156a-5p	699:717		-21,99	-21,00	-20,70		3
3utr|ENST00000374839|ZC4H2	ath-miR156a-5p	576:596		-20,60	-21,10	-21,30		3
3utr|ENST00000375604|MCF2L	ath-miR156a-5p	1846:1863	17		-24,80	-25,80		3
3utr|ENST00000376230|ZNF550	ath-miR156a-5p	2010:2029		-22,13	-20,80	-21,00		3
3utr|ENST00000376451|KIAA1217	ath-miR156a-5p	928:946		-19,16	-18,30	-26,61		3
3utr|ENST00000376550|XYLT2	ath-miR166a-3p	324:349		-29,22	-18,10	-26,00		3
3utr|ENST00000376572|KIAA2013	ath-miR156a-5p	286:298	12		-18,30	-21,52		3
3utr|ENST00000377275|ARL5B	ath-miR156a-5p	2022:2036	14		-25,60	-26,00		3
3utr|ENST00000379416|XDH	ath-miR156a-5p	1157:1176		-26,16	-26,90	-26,50		3
3utr|ENST00000379441|BAZ2A	ath-miR156a-5p	681:700		-20,19	-20,80	-20,71		3
3utr|ENST00000379472|KCNC1	ath-miR166a-3p	1054:1075		-28,86	-25,90	-25,00		3
3utr|ENST00000379731|B4GALT1	ath-miR166a-3p	2335:2356		-29,39	-25,90	-27,81		3
3utr|ENST00000379938|RREB1	ath-miR156a-5p	1013:1026	13		-19,80	-22,35		3
3utr|ENST00000380455|NQO2	ath-miR156a-5p	3936:3955		-20,21	-17,30	-19,10		3
3utr|ENST00000380620|B3GALT5	ath-miR156a-5p	8409:8428		-21,90	-19,60	-20,53		3
3utr|ENST00000380672|BNC2	ath-miR156a-5p	7778:7798		-22,28	-19,90	-20,80		3
3utr|ENST00000380769|WRNIP1	ath-miR156a-5p	1125:1143		-21,12	-20,20	-20,60		3

3utr|ENST00000380834|CENPO	ath-miR156a-5p	1076:1096		-19,75	-19,00	-22,40		3
3utr|ENST00000381024|ATAD2B	ath-miR156a-5p	928:941	13		-20,00	-20,40		3
3utr|ENST00000381151|SLC5A3	ath-miR156a-5p	4747:4760	13		-18,20	-19,90		3
3utr|ENST00000381359|SERF2	ath-miR156a-5p	682:695	13		-26,80	-27,20		3
3utr|ENST00000382095|SPATA13	ath-miR156a-5p	485:504	15	-26,45		-26,00		3
3utr|ENST00000382108|SPATA13	ath-miR156a-5p	485:504	15	-26,45		-26,00		3
3utr|ENST00000382545|KCNA1	ath-miR156a-5p	5309:5328		-21,21	-19,40	-20,40		3
3utr|ENST00000382850|NEDD4L	ath-miR156a-5p	3798:3817		-21,75	-20,20	-22,80		3
3utr|ENST00000383024|DGCR8	ath-miR156a-5p	1394:1413		-19,82	-19,10	-20,50		3
3utr|ENST00000389857|CCDC88C	ath-miR156a-5p	1019:1035	16		-27,60	-28,00		3
3utr|ENST00000391741|LAIR1	ath-miR156a-5p	36:55		-22,65	-22,50	-22,40		3
3utr|ENST00000393001|AMMECR1L	ath-miR156a-5p	1562:1577	15		-21,40	-21,50		3
3utr|ENST00000393315|TRIM7	ath-miR166a-3p	57:77		-25,25	-24,20	-29,97		3
3utr|ENST00000393319|TRIM7	ath-miR166a-3p	57:77		-25,25	-24,20	-29,97		3
3utr|ENST00000394232|SV2B	ath-miR156a-5p	1791:1811		-29,60	-26,60	-27,00		3
3utr|ENST00000394287|MED1	ath-miR156a-5p	278:298		-21,15	-19,90	-19,60		3
3utr|ENST00000394409|PPP2R2B	ath-miR156a-5p	3054:3073		-24,02	-18,30	-20,30		3
3utr|ENST00000395002|FAM13A	ath-miR156a-5p	1581:1595	14		-26,20	-26,80		3
3utr|ENST00000395751|SREBF1	ath-miR166a-3p	947:967		-28,21	-24,80	-25,36		3
3utr|ENST00000395756|SREBF1	ath-miR166a-3p	1009:1029		-28,21	-24,80	-25,36		3
3utr|ENST00000396184|PDE1C	ath-miR156a-5p	1450:1462	12		-17,50	-19,80		3
3utr|ENST00000396445|KIAA1217	ath-miR156a-5p	928:946		-19,16	-18,30	-26,61		3
3utr|ENST00000396502|CLEC12B	ath-miR156a-5p	1260:1280		-26,76	-23,90	-24,30		3
3utr|ENST00000396504|POLR3H	ath-miR156a-5p	3393:3405	12		-22,70	-22,50		3
3utr|ENST00000397030|MCF2L	ath-miR156a-5p	2218:2235	17		-24,80	-25,80		3
3utr|ENST00000397224|FOXRED2	ath-miR156a-5p	793:812		-20,20	-19,20	-22,16		3
3utr|ENST00000397341|ACIN1	ath-miR156a-5p	429:448		-21,64	-22,40	-22,30		3
3utr|ENST00000397554|UQCC1	ath-miR156a-5p	1520:1540	12	-19,05	-18,50			3

3utr|ENST00000397980|PHACTR2	ath-miR166a-3p	78:98		-25,67	-23,90	-23,80		3
3utr|ENST00000398004|SLC35E3	ath-miR156a-5p	12568:12588		-22,44	-21,20	-21,50		3
3utr|ENST00000398261|WHSC1	ath-miR166a-3p	4616:4638		-36,15	-30,30	-31,70		3
3utr|ENST00000398309|DLG2	ath-miR156a-5p	3285:3305		-22,94	-20,90	-20,90		3
3utr|ENST00000398514|DPYSL3	ath-miR156a-5p	909:928		-22,18	-20,20	-20,90		3
3utr|ENST00000398653|THADA	ath-miR156a-5p	4459:4477		-22,32	-22,80	-23,60		3
3utr|ENST00000398835|XPO5	ath-miR156a-5p	664:683		-20,18	-18,50	-19,90		3
3utr|ENST00000399701|HSPA12B	ath-miR166a-3p	183:204		-29,04	-27,60	-27,60		3
3utr|ENST00000399949|SPATA13	ath-miR156a-5p	485:504	15	-26,45		-26,00		3
3utr|ENST00000404127|ARHGAP4	ath-miR156a-5p	348:366		-19,86	-20,50	-20,60		3
3utr|ENST00000405640|C22orf29	ath-miR156a-5p	469:490		-19,38	-19,40	-19,70		3
3utr|ENST00000406785|SLC8A1	ath-miR156a-5p	2022:2035	13		-19,10	-19,60		3
3utr|ENST00000407472|C22orf29	ath-miR156a-5p	469:490		-19,38	-19,40	-19,70		3
3utr|ENST00000407755|DGCR8	ath-miR156a-5p	1394:1413		-19,82	-19,10	-20,50		3
3utr|ENST00000408936|DAB2IP	ath-miR156a-5p	1739:1758		-22,18	-21,10	-21,00		3
3utr|ENST00000409939|TBC1D10B	ath-miR156a-5p	490:511		-28,54	-26,40	-29,00		3
3utr|ENST00000409992|FBXO36	ath-miR156a-5p	54:73		-20,15	-21,10	-23,60		3
3utr|ENST00000416284|FAM19A2	ath-miR156a-5p	372:392		-19,80	-19,60	-19,60		3
3utr|ENST00000416671|CHEK2	ath-miR172a	336:356	14	-21,10	-21,80			3
3utr|ENST00000417588|CHEK2	ath-miR172a	143:163	14	-21,10	-21,80			3
3utr|ENST00000417826|CCDC103	ath-miR156a-5p	2189:2208		-21,88	-18,70	-20,69		3
3utr|ENST00000418556|LAIR1	ath-miR156a-5p	268:287		-22,65	-22,50	-22,40		3
3utr|ENST00000418635|PPARD	ath-miR156a-5p	469:487		-20,20	-19,70	-20,80		3
3utr|ENST00000420619|LDLRAD1	ath-miR156a-5p	850:870		-21,33	-20,60	-22,80		3
3utr|ENST00000420808|NISCH	ath-miR156a-5p	514:534		-23,45	-21,50	-21,80		3
3utr|ENST00000422067|TRIM7	ath-miR166a-3p	57:77		-25,25	-24,20	-29,97		3
3utr|ENST00000424629|ZNF561	ath-miR156a-5p	605:625		-23,20	-18,80	-20,00		3
3utr|ENST00000424834|SPATA13	ath-miR156a-5p	485:504	15	-26,45		-26,00		3

3utr|ENST00000426717|DLG2	ath-miR156a-5p	3285:3305		-22,94	-20,90	-20,90		3
3utr|ENST00000429136|ATP13A3	ath-miR156a-5p	2029:2049		-20,92	-20,90	-21,30		3
3utr|ENST00000433028|CHEK2	ath-miR172a	571:591	14	-21,10	-21,80			3
3utr|ENST00000433197|ERN1	ath-miR156a-5p	2317:2337		-25,92	-25,10	-25,40		3
3utr|ENST00000435753|DDX11	ath-miR156a-5p	2085:2104		-23,76	-22,40	-21,50		3
3utr|ENST00000435817|FCHSD1	ath-miR156a-5p	218:231	13		-19,80	-20,57		3
3utr|ENST00000436881|C9orf172	ath-miR166a-3p	1084:1104		-22,85	-19,80	-19,74		3
3utr|ENST00000437205|ABCC5	ath-miR156a-5p	3656:3674		-21,31	-21,50	-20,90		3
3utr|ENST00000437844|ZEB1	ath-miR156a-5p	5742:5761		-22,90	-20,40	-21,40		3
3utr|ENST00000438926|SLC12A3	ath-miR156a-5p	598:620		-22,87	-16,40	-22,40		3
3utr|ENST00000439040|ATP13A3	ath-miR156a-5p	2029:2049		-20,92	-20,90	-21,30		3
3utr|ENST00000439346|CHEK2	ath-miR172a	143:163	14	-21,10	-21,80			3
3utr|ENST00000441020|LANCL1	ath-miR156a-5p	1598:1616		-24,30	-22,80	-24,10		3
3utr|ENST00000441787|PRDM15	ath-miR166a-3p	22:41		-31,83	-28,40	-28,40		3
3utr|ENST00000443276|GDPD5	ath-miR156a-5p	131:152		-24,44	-22,20	-22,10		3
3utr|ENST00000443314|LANCL1	ath-miR156a-5p	1598:1616		-24,30	-22,80	-24,10		3
3utr|ENST00000445816|SERF2	ath-miR156a-5p	245:258	13		-26,80	-27,20		3
3utr|ENST00000446923|ZEB1	ath-miR156a-5p	2227:2246		-22,90	-20,40	-21,40		3
3utr|ENST00000447096|DNAJC25	ath-miR156a-5p	1844:1864		-23,02	-21,20	-21,00		3
3utr|ENST00000447310|ZNF550	ath-miR156a-5p	2596:2615		-22,13	-20,80	-21,00		3
3utr|ENST00000447788|ZC4H2	ath-miR156a-5p	557:577		-20,60	-21,10	-21,30		3
3utr|ENST00000448077|PPARD	ath-miR156a-5p	469:487		-20,20	-19,70	-20,80		3
3utr|ENST00000448511|CHEK2	ath-miR172a	238:258	14	-21,10	-21,80			3
3utr|ENST00000448940|CDC42BPA	ath-miR156a-5p	2167:2187		-22,09	-21,60	-22,50		3
3utr|ENST00000449395|PRDM15	ath-miR166a-3p	22:41		-31,83	-28,40	-28,40		3
3utr|ENST00000450366|LANCL1	ath-miR156a-5p	1598:1616		-24,30	-22,80	-24,10		3
3utr|ENST00000452281|STAT1	ath-miR156a-5p	841:855	14		-18,20	-21,51		3
3utr|ENST00000452398|KCNH8	ath-miR156a-5p	3427:3447		-24,26	-22,20	-22,20		3

3utr|ENST00000456986|NEDD4L	ath-miR156a-5p	3798:3817		-21,75	-20,20	-22,80		3
3utr|ENST00000457177|ZNF550	ath-miR156a-5p	1663:1682		-22,13	-20,80	-21,00		3
3utr|ENST00000457367|EXOG	ath-miR156a-5p	1565:1588		-25,77	-20,10	-24,30		3
3utr|ENST00000457657|ACIN1	ath-miR156a-5p	429:448		-21,64	-22,40	-22,30		3
3utr|ENST00000463589|DNAJC25	ath-miR156a-5p	1906:1926		-23,02	-21,20	-21,00		3
3utr|ENST00000472232|BAG1	ath-miR156a-5p	185:203		-20,43	-21,30	-20,80		3
3utr|ENST00000472487|MYSM1	ath-miR156a-5p	3717:3736		-20,74	-22,70	-21,60		3
3utr|ENST00000473706|CENPO	ath-miR156a-5p	1076:1096		-19,75	-19,00	-22,40		3
3utr|ENST00000473758|ACIN1	ath-miR156a-5p	3278:3297		-21,64	-22,40	-22,30		3
3utr|ENST00000476832|ALG2	ath-miR156a-5p	278:298		-20,28	-19,00	-19,30		3
3utr|ENST00000488380|NISCH	ath-miR156a-5p	893:913		-23,45	-21,50	-21,80		3
3utr|ENST00000488690|AHI1	ath-miR156a-5p	223:241		-19,31	-19,40	-20,00		3
3utr|ENST00000493950|ADGB	ath-miR166a-3p	2300:2319		-28,73	-24,90	-19,95		3
3utr|ENST00000495632|BTN2A2	ath-miR156a-5p	134:155		-27,36	-23,60	-25,10		3
3utr|ENST00000497979|RINT1	ath-miR156a-5p	793:805	12		-19,90	-21,50		3
3utr|ENST00000503128|WHSC1	ath-miR166a-3p	4616:4638		-36,15	-30,30	-31,70		3
3utr|ENST00000505907|MAN2B2	ath-miR156a-5p	3966:3986		-24,07	-21,60	-22,30		3
3utr|ENST00000507978|ASTE1	ath-miR156a-5p	110:128		-27,23	-25,90	-26,50		3
3utr|ENST00000509756|GNPDA2	ath-miR156a-5p	2588:2601	13		-19,30	-19,30		3
3utr|ENST00000517878|SLC45A4	ath-miR166a-3p	88:107		-31,36	-28,90	-28,60		3
3utr|ENST00000519067|SLC45A4	ath-miR166a-3p	707:726		-31,36	-28,90	-28,60		3
3utr|ENST00000521861|EIF3H	ath-miR156a-5p	836:855		-20,95	-19,60	-22,00		3
3utr|ENST00000522126|FCHSD1	ath-miR156a-5p	848:861	13		-19,80	-20,57		3
3utr|ENST00000522783|FCHSD1	ath-miR156a-5p	379:392	13		-19,80	-20,57		3
3utr|ENST00000523756|TG	ath-miR156a-5p	1369:1387		-30,67	-29,70	-29,90		3
3utr|ENST00000526143|BCL9L	ath-miR156a-5p	458:478		-19,98	-20,50	-21,00		3
3utr|ENST00000527056|MS4A4A	ath-miR166a-3p	472:485	13		-25,20	-25,82		3
3utr|ENST00000527820|GDPD5	ath-miR156a-5p	131:152		-24,44	-22,20	-22,10		3

3utr|ENST00000529945|PDE4DIP	ath-miR156a-5p	1927:1948		-23,37	-20,90	-20,90		3
3utr|ENST00000529950|MS4A4A	ath-miR166a-3p	458:471	13		-25,20	-25,82		3
3utr|ENST00000530254|MYO18A	ath-miR156a-5p	5266:5285		-22,97	-19,20	-19,10		3
3utr|ENST00000531709|NXF1	ath-miR156a-5p	1714:1734		-25,09	-24,40	-25,30		3
3utr|ENST00000536440|ACACB	ath-miR156a-5p	519:541		-22,40	-18,10	-21,90		3
3utr|ENST00000536980|MEFV	ath-miR156a-5p	970:990		-24,83	-24,20	-24,60		3
3utr|ENST00000537098|CMIP	ath-miR156a-5p	1256:1274		-21,83	-20,30	-20,80		3
3utr|ENST00000537682|MEFV	ath-miR156a-5p	970:990		-24,83	-24,20	-24,60		3
3utr|ENST00000538326|MEFV	ath-miR156a-5p	1319:1339		-24,83	-24,20	-24,60		3
3utr|ENST00000538404|LAMTOR1	ath-miR156a-5p	120:139		-20,35	-19,40	-19,60		3
3utr|ENST00000539155|CLEC12B	ath-miR156a-5p	1753:1773		-26,76	-23,90	-24,30		3
3utr|ENST00000539778|CMIP	ath-miR156a-5p	1256:1274		-21,83	-20,30	-20,80		3
3utr|ENST00000540176|STAT1	ath-miR156a-5p	841:855	14		-18,20	-21,51		3
3utr|ENST00000541543|CEP41	ath-miR156a-5p	4222:4241		-24,42	-21,70	-22,20		3
3utr|ENST00000542898|MEFV	ath-miR156a-5p	970:990		-24,83	-24,20	-24,60		3
3utr|ENST00000543251|UTP15	ath-miR156a-5p	2359:2380		-25,26	-25,20	-24,90		3
3utr|ENST00000543415|GTF2H3	ath-miR156a-5p	6:24		-21,72	-20,70	-21,10		3
3utr|ENST00000545928|LDLRAD1	ath-miR156a-5p	850:870		-21,33	-20,60	-22,80		3
3utr|ENST00000549379|FAM19A2	ath-miR156a-5p	610:630		-19,80	-19,60	-19,60		3
3utr|ENST00000550003|FAM19A2	ath-miR156a-5p	372:392		-19,80	-19,60	-19,60		3
3utr|ENST00000551590|TCTN1	ath-miR166a-3p	347:366		-28,16	-23,40	-23,30		3
3utr|ENST00000551619|FAM19A2	ath-miR156a-5p	372:392		-19,80	-19,60	-19,60		3
3utr|ENST00000551812|BAZ2A	ath-miR156a-5p	681:700		-20,19	-20,80	-20,71		3
3utr|ENST00000554752|MAP3K9	ath-miR156a-5p	4703:4715	12		-19,60	-21,70		3
3utr|ENST00000556726|CCDC88C	ath-miR156a-5p	2940:2956	16		-27,60	-28,00		3
3utr|ENST00000557515|ACIN1	ath-miR156a-5p	429:448		-21,64	-22,40	-22,30		3
3utr|ENST00000558373|CTDSPL2	ath-miR156a-5p	1954:1973		-21,31	-19,90	-20,60		3
3utr|ENST00000563236|SLC12A3	ath-miR156a-5p	598:620		-22,87	-16,40	-22,40		3

3utr|ENST00000566454|SLC7A6	ath-miR156a-5p	1132:1152		-23,43	-23,20	-23,90		3
3utr|ENST00000568530|PDPR	ath-miR156a-5p	3022:3042		-19,28	-16,70	-20,93		3
3utr|ENST00000568684|WWP2	ath-miR156a-5p	1510:1529		-21,15	-22,40	-21,90		3
3utr|ENST00000571589|MKL2	ath-miR156a-5p	1113:1126	13		-22,70	-23,30		3
3utr|ENST00000573331|TMEM256-PLSCR3	ath-miR166a-3p	1520:1534	14		-31,20	-30,50		3
3utr|ENST00000575112|RPAIN	ath-miR156a-5p	2763:2784		-21,30	-22,40	-22,70		3
3utr|ENST00000579624|CNDP2	ath-miR156a-5p	617:636		-20,30	-21,40	-20,30		3
3utr|ENST00000579847|CNDP2	ath-miR156a-5p	807:826		-20,30	-21,40	-20,30		3
3utr|ENST00000581104|NUP85	ath-miR156a-5p	1035:1054		-22,68	-22,10	-22,50		3
3utr|ENST00000586870|FAM134C	ath-miR156a-5p	354:373		-20,25	-23,20	-23,10		3
3utr|ENST00000589422|ZNF500	ath-miR166a-3p	1040:1052	12		-25,40	-25,10		3
3utr|ENST00000589522|ZNF233	ath-miR156a-5p	312:331		-19,56	-19,00	-19,80		3
3utr|ENST00000589797|FAM134C	ath-miR156a-5p	939:958		-20,25	-23,20	-23,10		3
3utr|ENST00000591662|CNTNAP1	ath-miR166a-3p	378:398		-29,16	-25,40	-25,90		3
3utr|ENST00000594030|ZNF765	ath-miR156a-5p	3891:3911		-23,26	-23,30	-24,10		3
3utr|ENST00000596814|VRK3	ath-miR156a-5p	853:872		-22,97	-21,50	-21,50		3
3utr|ENST00000599598|FBXO17	ath-miR156a-5p	50:71		-25,29	-22,30	-22,30		3
3utr|ENST00000601341|VRK3	ath-miR156a-5p	324:343		-22,97	-21,50	-21,50		3
3utr|ENST00000602307|PTPN23	ath-miR166a-3p	2954:2967	13		-24,70	-24,30		3
3utr|ENST00000605057|ACIN1	ath-miR156a-5p	429:448		-21,64	-22,40	-22,30		3
3utr|ENST00000606888|ANKRD34A	ath-miR156a-5p	708:731		-24,24	-19,00	-24,90		3
3utr|ENST00000608192|DZANK1	ath-miR156a-5p	203:222		-21,50	-20,60	-21,00		3
3utr|ENST00000609438|GPRIN3	ath-miR156a-5p	2009:2029		-23,22	-19,20	-20,10		3
3utr|ENST00000610888|MED22	ath-miR156a-5p	2329:2349		-21,95	-19,80	-22,70		3
3utr|ENST00000611109|RREB1	ath-miR156a-5p	1013:1026	13		-19,80	-22,35		3
3utr|ENST00000611463|ZNF2	ath-miR156a-5p	1198:1218		-22,79	-22,10	-22,90		3
3utr|ENST00000611725|SNX13	ath-miR156a-5p	880:893	13		-21,90	-22,40		3

3utr|ENST00000611745|SRRM3	ath-miR166a-3p	1344:1359	15		-34,40	-34,60		3
3utr|ENST00000611979|MAP3K9	ath-miR156a-5p	4703:4715	12		-19,60	-21,70		3
3utr|ENST00000612909|LAIR1	ath-miR156a-5p	36:55		-22,65	-22,50	-22,40		3
3utr|ENST00000614493|MED22	ath-miR156a-5p	2329:2349		-21,95	-19,80	-22,70		3
3utr|ENST00000614977|CPEB1	ath-miR156a-5p	687:700	13		-23,60	-23,30		3
3utr|ENST00000616154|CDX1	ath-miR166a-3p	72:86	14		-26,60	-29,70		3
3utr|ENST00000616933|SLC12A5	ath-miR166a-3p	2614:2633		-23,14	-22,00	-21,70		3
3utr|ENST00000617923|ZNF2	ath-miR156a-5p	1198:1218		-22,79	-22,10	-22,90		3
3utr|ENST00000618043|SLC7A6	ath-miR156a-5p	1845:1865		-23,43	-23,20	-23,90		3
3utr|ENST00000618156|PPP1R2	ath-miR156a-5p	897:916		-24,36	-20,40	-23,10		3
3utr|ENST00000618524|LAIR1	ath-miR156a-5p	268:287		-22,65	-22,50	-22,40		3
3utr|ENST00000619199|ATP13A3	ath-miR156a-5p	147:167		-20,92	-20,90	-21,30		3
3utr|ENST00000619701|ABCG4	ath-miR156a-5p	1241:1262		-25,16	-24,70	-24,80		3
3utr|ENST00000619732|MECP2	ath-miR156a-5p	5584:5602		-22,75	-22,90	-22,40		3
3utr|ENST00000620340|RPS6KA6	ath-miR156a-5p	1835:1855		-23,44	-19,90	-20,50		3
3utr|ENST00000621537|CMIP	ath-miR156a-5p	1256:1274		-21,83	-20,30	-20,80		3
3utr|ENST00000622663|SASH1	ath-miR156a-5p	363:382		-22,86	-22,60	-23,20		3
3utr|ENST00000622766|MYSM1	ath-miR156a-5p	3717:3736		-20,74	-22,70	-21,60		3
3utr|ENST00000626118|MED22	ath-miR156a-5p	2329:2349		-21,95	-19,80	-22,70		3
3utr|ENST00000629975|MED22	ath-miR156a-5p	2329:2349		-21,95	-19,80	-22,70		3
3utr|ENST00000632883|CPEB1	ath-miR156a-5p	687:700	13		-23,60	-23,30		3
5utr|ENST00000007516|NDUFAB1	ath-miR166a-3p	10:30		-29,08	-26,40	-25,90		3
5utr|ENST00000083182|APPBP2	ath-miR156a-5p	66:84		-21,49	-21,70		-19,20	3
5utr|ENST00000174653|AP3M2	ath-miR156a-5p	254:268	14		-20,90	-22,93		3
5utr|ENST00000246949|DNASE1	ath-miR172a	1946:1965		-22,26	-22,40	-19,70		3
5utr|ENST00000253109|ANGPTL6	ath-miR156a-5p	99:117		-21,39	-20,20	-20,90		3
5utr|ENST00000254301|LGALS3	ath-miR166a-3p	99:111	12		-24,10	-29,70		3
5utr|ENST00000262096|ZDHHC2	osa-miR168a-5p	137:157	12	-33,80			-29,60	3

5utr|ENST00000262370|MGRN1	ath-miR166a-3p	80:92	12		-26,40	-27,00		3
5utr|ENST00000263552|TBXAS1	ath-miR156a-5p	289:311		-24,57	-19,90	-24,30		3
5utr|ENST00000276198|HTR2C	ath-miR156a-5p	146:166		-21,35	-19,30	-20,50		3
5utr|ENST00000289373|TMSB15A	ath-miR166a-3p	42:63		-28,53	-25,30	-25,60		3
5utr|ENST00000309784|TRIM59	ath-miR166a-3p	83:101		-28,56	-26,40	-26,70		3
5utr|ENST00000312423|SWSAP1	ath-miR166a-3p	38:50	12		-32,20	-34,60		3
5utr|ENST00000323289|ADGRB1	ath-miR166a-3p	114:137		-25,52	-20,20	-23,00		3
5utr|ENST00000326925|NDUFAF3	ath-miR166a-3p	415:428	13		-22,30	-24,40		3
5utr|ENST00000336425|TBXAS1	ath-miR156a-5p	137:159		-24,57	-19,90	-24,30		3
5utr|ENST00000357736|MAFG	ath-miR166a-3p	20:33	13		-28,60	-30,60		3
5utr|ENST00000359450|TIAF1	ath-miR156a-5p	4206:4225		-22,97	-19,20	-19,10		3
5utr|ENST00000359963|CCT8L2	ath-miR166a-3p	87:108		-26,93	-23,70	-24,52		3
5utr|ENST00000371950|HTR2C	ath-miR156a-5p	142:162		-21,35	-19,30	-20,50		3
5utr|ENST00000371951|HTR2C	ath-miR156a-5p	146:166		-21,35	-19,30	-20,50		3
5utr|ENST00000372043|GBGT1	ath-miR156a-5p	1:18	12	-21,76			-19,50	3
5utr|ENST00000379251|SAT1	ath-miR166a-3p	133:153	13	-28,19			-25,40	3
5utr|ENST00000379253|SAT1	ath-miR166a-3p	133:153	13	-28,19			-25,40	3
5utr|ENST00000379254|SAT1	ath-miR166a-3p	133:153	13	-28,19			-25,40	3
5utr|ENST00000379270|SAT1	ath-miR166a-3p	133:153	13	-28,19			-25,40	3
5utr|ENST00000381418|HR	ath-miR166a-3p	507:519	12		-24,80	-28,91		3
5utr|ENST00000383827|TTLL3	ath-miR156a-5p	969:990		-21,06	-19,30	-19,00		3
5utr|ENST00000396926|AP3M2	ath-miR156a-5p	139:153	14		-20,90	-22,93		3
5utr|ENST00000399510|DLG4	ath-miR156a-5p	354:373		-20,32	-19,70	-19,60		3
5utr|ENST00000413454|NUP62	ath-miR156a-5p	102:121		-19,95	-20,60	-19,40		3
5utr|ENST00000415496|MGRN1	ath-miR166a-3p	46:58	12		-26,40	-27,00		3
5utr|ENST00000416849|TBXAS1	ath-miR156a-5p	289:311		-24,57	-19,90	-24,30		3
5utr|ENST00000418878|CXCR2	ath-miR156a-5p	19:38		-19,87	-19,20	-19,90		3
5utr|ENST00000444677|GARNL3	ath-miR166a-3p	29:42	13		-25,80	-28,90		3

5utr|ENST00000455274|TTLL3	ath-miR156a-5p	38:59		-21,06	-19,30	-19,00		3
5utr|ENST00000460265|LMNB1	ath-miR166a-3p	261:274	13		-25,30	-26,15		3
5utr|ENST00000468904|XKRX	ath-miR156a-5p	382:402		-25,10	-25,00	-25,40		3
5utr|ENST00000471155|TRIM59	ath-miR166a-3p	19:37		-28,56	-26,40	-26,70		3
5utr|ENST00000471396|TRIM59	ath-miR166a-3p	17:35		-28,56	-26,40	-26,70		3
5utr|ENST00000473661|TTLL3	ath-miR156a-5p	780:801		-21,06	-19,30	-19,00		3
5utr|ENST00000474145|SLC2A5	ath-miR166a-3p	110:130		-33,91	-31,70	-31,80		3
5utr|ENST00000476183|LIME1	ath-miR156a-5p	12:30	12	-20,54			-19,50	3
5utr|ENST00000483754|RP11-432B6.3	ath-miR166a-3p	83:101		-28,56	-26,40	-26,70		3
5utr|ENST00000495558|VWA1	ath-miR156a-5p	245:264		-19,87	-18,70	-20,01		3
5utr|ENST00000496222|TRIM59	ath-miR166a-3p	14:32		-28,56	-26,40	-26,70		3
5utr|ENST00000497245|LRCH4	ath-miR166a-3p	522:542			-23,90	-23,90	-23,40	3
5utr|ENST00000498603|SEC61B	ath-miR166a-3p	107:119	12		-24,70	-25,58		3
5utr|ENST00000502274|SH3TC2	ath-miR156a-5p	1388:1406		-22,32	-20,90	-21,31		3
5utr|ENST00000511438|KIAA1257	ath-miR156a-5p	258:278		-21,76	-19,30		-20,50	3
5utr|ENST00000517865|AP3M2	ath-miR156a-5p	1667:1681	14		-20,90	-22,93		3
5utr|ENST00000517894|ADGRB1	ath-miR166a-3p	825:848		-25,52	-20,20	-23,00		3
5utr|ENST00000518421|AP3M2	ath-miR156a-5p	263:277	14		-20,90	-22,93		3
5utr|ENST00000520339|CTNNA1	ath-miR156a-5p	225:247			-21,20	-22,40	-20,40	3
5utr|ENST00000521208|ADGRB1	ath-miR166a-3p	576:599		-25,52	-20,20	-23,00		3
5utr|ENST00000522288|AP3M2	ath-miR156a-5p	128:142	14		-20,90	-22,93		3
5utr|ENST00000530055|NADSYN1	ath-miR156a-5p	555:576			-17,10	-20,50	-19,30	3
5utr|ENST00000530375|AP3M2	ath-miR156a-5p	65:79	14		-20,90	-22,93		3
5utr|ENST00000531804|FAM167A	ath-miR156a-5p	417:436		-20,31	-20,40	-20,70		3
5utr|ENST00000536343|MGRN1	ath-miR166a-3p	28:40	12		-26,40	-27,00		3
5utr|ENST00000537729|LRRC43	ath-miR166a-3p	150:162	12		-23,50	-27,10		3
5utr|ENST00000546474|BRF1	ath-miR156a-5p	7159:7180		-24,21	-24,40	-23,80		3

5utr|ENST00000547562|BRF1	ath-miR166a-3p	70:90	13	-29,97			-25,80	3
5utr|ENST00000549780|TGIF1	ath-miR156a-5p	42:61	14	-24,62			-22,30	3
5utr|ENST00000561871|GANC	ath-miR156a-5p	416:435		-19,87	-20,10		-20,20	3
5utr|ENST00000563788|YPEL3	ath-miR166a-3p	105:123		-31,05	-28,00	-27,60		3
5utr|ENST00000566595|YPEL3	ath-miR166a-3p	102:120		-31,05	-28,00	-27,60		3
5utr|ENST00000574686|MAFG	ath-miR166a-3p	14:27	13		-28,60	-30,60		3
5utr|ENST00000577495|OGFOD3	ath-miR166a-3p	93:105	12		-24,60	-26,42		3
5utr|ENST00000580163|KSR1	ath-miR166a-3p	156:176		-27,22	-24,60	-25,10		3
5utr|ENST00000587747|MGRN1	ath-miR166a-3p	24:36	12		-26,40	-27,00		3
5utr|ENST00000596217|NUP62	ath-miR156a-5p	1360:1379		-19,95	-20,60	-19,40		3
5utr|ENST00000597017|KDELR1	ath-miR156a-5p	240:258		-21,89	-20,60	-22,50		3
5utr|ENST00000615353|HIST1H4I	ath-miR166a-3p	635:655			-22,10	-22,20	-20,60	3
5utr|ENST00000615747|DGCR2	ath-miR166a-3p	200:212	12		-24,10	-23,80		3
5utr|ENST00000619071|LRCH4	ath-miR166a-3p	275:295			-23,90	-23,90	-23,40	3
5utr|ENST00000630715|MICAL1	ath-miR166a-3p	42:62	13	-29,02			-25,30	3
cds|ENST00000007722|ITGA3	ath-miR167a-5p	757:778		-21,76	-23,30	-21,70		3
cds|ENST00000017003|XYLT2	ath-miR166a-3p	2440:2465		-29,22	-18,10	-26,00		3
cds|ENST00000024061|SLC45A4	ath-miR166a-3p	2355:2374		-31,36	-28,90	-28,60		3
cds|ENST00000033079|FAM13B	osa-miR168a-5p	2277:2295		-24,60		-25,60	-21,40	3
cds|ENST00000038176|NSMAF	ath-miR172a	211:233	14	-24,16		-21,20		3
cds|ENST00000066544|CDC27	ath-miR156a-5p	1649:1668		-21,79	-21,50		-19,70	3
cds|ENST00000075430|CTDP1	ath-miR166a-3p	1777:1797		-27,07	-23,10	-23,90		3
cds|ENST00000085219|CD22	ath-miR167a-5p	643:665		-23,22	-22,30	-21,20		3
cds|ENST00000160298|CAMSAP3	ath-miR156a-5p	1068:1086		-20,89	-20,20	-20,80		3
cds|ENST00000200181|ITGB4	ath-miR167a-5p	232:253		-22,77	-23,40	-21,40		3
cds|ENST00000206765|TGM1	ath-miR167a-5p	1520:1540		-23,83	-22,10	-20,90		3
cds|ENST00000216194|ADSL	ath-miR167a-5p	1259:1278		-20,09	-22,10	-20,20		3
cds|ENST00000217926|H2BFWT	osa-miR168a-5p	372:390		-27,35		-23,70	-21,90	3

cds|ENST00000218056|WDR13	ath-miR156a-5p	1222:1241			-21,80	-22,50	-20,10	3
cds|ENST00000219611|CAPN15	ath-miR166a-3p	2229:2249		-25,05	-22,50	-21,90		3
cds|ENST00000222584|SP4	ath-miR172a	2288:2310		-21,00	-23,00	-20,30		3
cds|ENST00000223145|GLCCI1	ath-miR156a-5p	1040:1060		-21,26	-19,40	-19,20		3
cds|ENST00000224721|CDH23	ath-miR156a-5p	9544:9563		-20,84	-19,40	-19,80		3
cds|ENST00000225927|NAGLU	ath-miR166a-3p	303:315	12		-24,80	-24,80		3
cds|ENST00000228955|GTF2H3	ath-miR156a-5p	632:650		-21,72	-20,70	-21,10		3
cds|ENST00000230538|LAMA4	ath-miR172a	2790:2810	16	-20,87	-22,20			3
cds|ENST00000231484|PCDH12	ath-miR166a-3p	2798:2812	14		-28,30	-28,70		3
cds|ENST00000233099|HEATR5B	ath-miR167a-5p	3578:3597		-24,93	-24,60	-22,40		3
cds|ENST00000234142|GREB1	ath-miR156a-5p	1888:1909		-28,56	-24,50	-24,40		3
cds|ENST00000241256|GHSR	ath-miR156a-5p	664:683		-19,67	-19,40	-19,90		3
cds|ENST00000241274|SLITRK3	ath-miR156a-5p	952:973		-21,67	-18,30	-21,71		3
cds|ENST00000242351|ZC3HAV1	ath-miR156a-5p	1764:1786		-24,46	-21,10	-21,10		3
cds|ENST00000243964|SLC12A5	ath-miR166a-3p	3296:3315		-23,14	-22,00	-21,70		3
cds|ENST00000244007|PLCG1	ath-miR167a-5p	823:841		-21,11	-21,30	-19,40		3
cds|ENST00000244174|IL9R	ath-miR156a-5p	1518:1537		-20,29	-20,10	-20,40		3
cds|ENST00000245544|NUP85	ath-miR156a-5p	1667:1686		-22,68	-22,10	-22,50		3
cds|ENST00000249284|TAS2R16	ath-miR156a-5p	156:176		-21,85	-21,50	-22,00		3
cds|ENST00000251871|MED17	ath-miR156a-5p	1568:1581	13		-18,30	-20,70		3
cds|ENST00000252015|TRPC4AP	ath-miR166a-3p	146:160	14		-27,80	-28,00		3
cds|ENST00000254605|RRP8	ath-miR167a-5p	1324:1346		-19,89	-12,10	-19,00		3
cds|ENST00000256646|NOTCH2	ath-miR167a-5p	4676:4696		-20,93	-21,70	-19,40		3
cds|ENST00000257700|RINT1	ath-miR156a-5p	1188:1200	12		-19,90	-21,50		3
cds|ENST00000259362|OR13C9	ath-miR172a	666:686		-24,09	-24,90	-22,30		3
cds|ENST00000261646|SREBF1	ath-miR166a-3p	2316:2336		-28,21	-24,80	-25,36		3
cds|ENST00000262318|CLCN7	ath-miR166a-3p	15:35		-25,05	-21,80	-22,10		3
cds|ENST00000262410|MAPT	ath-miR156a-5p	1325:1337	12		-19,90	-21,76		3

cds|ENST00000262547|DZANK1	ath-miR156a-5p	664:683		-21,50	-20,60	-21,00		3
cds|ENST00000264276|ALS2	ath-miR166a-3p	43:63	12	-30,19		-31,60		3
cds|ENST00000264638|CNTNAP1	ath-miR166a-3p	2617:2637		-29,16	-25,40	-25,90		3
cds|ENST00000264992|ASTE1	ath-miR156a-5p	1613:1631		-27,23	-25,90	-26,50		3
cds|ENST00000265351|XPO5	ath-miR156a-5p	2692:2711		-20,18	-18,50	-19,90		3
cds|ENST00000265562|PTPN23	ath-miR166a-3p	3277:3290	13		-24,70	-24,30		3
cds|ENST00000265742|ANKIB1	ath-miR156a-5p	926:946		-26,02	-22,60	-22,70		3
cds|ENST00000265806|R3HCC1	ath-miR167a-5p	176:196		-20,00	-20,50	-19,30		3
cds|ENST00000265960|MAPKAP1	ath-miR166a-3p	1039:1059		-32,75	-30,60	-30,70		3
cds|ENST00000269844|PRDM15	ath-miR166a-3p	2669:2688		-31,83	-28,40	-28,40		3
cds|ENST00000271854|NUP210L	ath-miR172a	4661:4681	14	-21,10	-21,80			3
cds|ENST00000273153|CSRNP1	ath-miR156a-5p	99:118		-19,54	-19,20	-23,20		3
cds|ENST00000273814|DGKQ	ath-miR166a-3p	340:352	12		-24,90	-26,10		3
cds|ENST00000274498|ARHGAP26	ath-miR166a-3p	494:513		-27,86	-24,00	-24,60		3
cds|ENST00000280481|FREM2	ath-miR156a-5p	7102:7123		-20,42	-18,80	-19,10		3
cds|ENST00000281416|MFSD6	ath-miR156a-5p	898:919		-22,53	-20,10	-20,20		3
cds|ENST00000282406|PLEKHH2	ath-miR156a-5p	4428:4446		-23,55	-22,60	-22,70		3
cds|ENST00000282516|NIPBL	ath-miR166a-3p	657:679		-19,84	-16,80	-19,90		3
cds|ENST00000284311|GPR15	ath-miR156a-5p	493:513		-19,81	-20,30	-20,10		3
cds|ENST00000285243|ANKRD40	ath-miR156a-5p	656:670	14		-16,20	-21,70		3
cds|ENST00000286827|TIAM1	ath-miR156a-5p	304:325		-24,09	-20,40	-22,80		3
cds|ENST00000288368|PREX2	ath-miR156a-5p	3956:3975		-21,48	-19,20	-19,50		3
cds|ENST00000292616|LRWD1	ath-miR166a-3p	1112:1126	14		-27,50	-28,74		3
cds|ENST00000293328|STAT5B	ath-miR166a-3p	302:321		-24,05	-22,00	-21,80		3
cds|ENST00000293350|ALDH16A1	ath-miR166a-3p	882:902		-27,05	-25,10	-28,10		3
cds|ENST00000293373|NCKAP1L	ath-miR166a-3p	1235:1257		-32,64	-23,60	-26,70		3
cds|ENST00000293373|NCKAP1L	ath-miR156a-5p	418:438		-21,84	-19,00	-19,30		3
cds|ENST00000294618|DOCK6	ath-miR166a-3p	3043:3055	12		-24,10	-29,90		3

cds|ENST00000296452|BSN	ath-miR166a-3p	8653:8673		-24,47	-23,10	-30,15		3
cds|ENST00000297183|ANKHD1	ath-miR156a-5p	1098:1117		-19,19	-19,50	-19,10		3
cds|ENST00000299543|CTDP1	ath-miR166a-3p	1420:1440		-27,07	-23,10	-23,90		3
cds|ENST00000300027|FANCI	ath-miR156a-5p	924:945		-21,41	-23,40	-24,00		3
cds|ENST00000303921|GPR37	ath-miR156a-5p	1807:1828		-22,61	-19,60	-21,60		3
cds|ENST00000304338|PPP4R4	ath-miR156a-5p	2253:2272		-20,52	-18,80	-21,80		3
cds|ENST00000304613|KNDC1	ath-miR166a-3p	2189:2209		-30,50	-27,40	-27,60		3
cds|ENST00000307179|USP8	ath-miR172a	619:639	13	-27,32	-9,87			3
cds|ENST00000307859|TRIM40	ath-miR156a-5p	93:105	12		-23,70	-24,40		3
cds|ENST00000308508|CSPG4	ath-miR166a-3p	3710:3729		-24,26	-22,60	-23,09		3
cds|ENST00000308811|ZNF596	ath-miR156a-5p	1279:1300		-19,16	-17,60	-19,30		3
cds|ENST00000309428|FAM134C	ath-miR156a-5p	787:806		-20,25	-23,20	-23,10		3
cds|ENST00000310317|SLC4A2	ath-miR166a-3p	2182:2205		-31,16	-26,40	-26,60		3
cds|ENST00000310373|GP6	ath-miR156a-5p	13:32		-21,61	-21,10	-21,60		3
cds|ENST00000310775|FANCI	ath-miR156a-5p	924:945		-21,41	-23,40	-24,00		3
cds|ENST00000313038|LAIR1	ath-miR156a-5p	493:512		-22,65	-22,50	-22,40		3
cds|ENST00000314393|ZHX2	ath-miR166a-3p	1996:2008	12		-24,50	-25,21		3
cds|ENST00000320552|ZNF596	ath-miR156a-5p	1279:1300		-19,16	-17,60	-19,30		3
cds|ENST00000321543|OR51L1	ath-miR156a-5p	84:104		-21,39	-19,60	-22,40		3
cds|ENST00000322285|SV2C	ath-miR156a-5p	731:744	13		-24,30	-25,00		3
cds|ENST00000328354|CHEK2	ath-miR172a	846:866	14	-21,10	-21,80			3
cds|ENST00000328405|KCNH8	ath-miR156a-5p	3083:3103		-24,26	-22,20	-22,20		3
cds|ENST00000329314|TRIM61	ath-miR156a-5p	313:327	14		-22,60	-22,70		3
cds|ENST00000329524|NFATC3	ath-miR156a-5p	928:946		-21,30	-20,50	-21,40		3
cds|ENST00000331433|CLCNKA	ath-miR156a-5p	890:908		-32,11	-30,00	-30,10		3
cds|ENST00000333884|GP6	ath-miR156a-5p	13:32		-21,61	-21,10	-21,60		3
cds|ENST00000337908|MS4A4A	ath-miR166a-3p	381:394	13		-25,20	-25,82		3
cds|ENST00000338316|ADCY2	ath-miR156a-5p	1280:1292	12		-26,20	-28,80		3

cds|ENST00000339777|LRRC43	ath-miR166a-3p	255:267	12		-23,50	-27,10		3
cds|ENST00000340381|TMEM201	ath-miR156a-5p	1473:1486	13		-22,80	-22,80		3
cds|ENST00000342187|TMEM91	ath-miR156a-5p	330:350		-22,50	-22,50	-21,90		3
cds|ENST00000344290|MAPT	ath-miR156a-5p	1325:1337	12		-19,90	-21,76		3
cds|ENST00000345063|TPRG1	ath-miR156a-5p	521:540		-23,37	-23,30	-23,30		3
cds|ENST00000345506|STAT5A	ath-miR166a-3p	343:363		-24,22	-22,90	-22,40		3
cds|ENST00000346183|NFATC3	ath-miR156a-5p	928:946		-21,30	-20,50	-21,40		3
cds|ENST00000347132|KCNQ4	ath-miR166a-3p	449:461	12		-20,90	-22,40		3
cds|ENST00000348231|LAIR1	ath-miR156a-5p	463:482		-22,65	-22,50	-22,40		3
cds|ENST00000349223|NFATC3	ath-miR156a-5p	928:946		-21,30	-20,50	-21,40		3
cds|ENST00000350060|ARHGAP4	ath-miR156a-5p	974:992		-19,86	-20,50	-20,60		3
cds|ENST00000355072|HTT	ath-miR156a-5p	5692:5711	15	-27,51		-24,30		3
cds|ENST00000355815|SREBF1	ath-miR166a-3p	2406:2426		-28,21	-24,80	-25,36		3
cds|ENST00000356385|TMEM91	ath-miR156a-5p	330:350		-22,50	-22,50	-21,90		3
cds|ENST00000357605|OR51D1	ath-miR156a-5p	560:578		-22,33	-20,70	-21,20		3
cds|ENST00000358334|MYOF	ath-miR166a-3p	3016:3036		-26,84	-25,40	-24,40		3
cds|ENST00000358334|MYOF	ath-miR156a-5p	4201:4224		-23,11	-17,20	-23,30		3
cds|ENST00000358866|DZANK1	ath-miR156a-5p	664:683		-21,50	-20,60	-21,00		3
cds|ENST00000359263|MYOF	ath-miR166a-3p	3055:3075		-26,84	-25,40	-24,40		3
cds|ENST00000359263|MYOF	ath-miR156a-5p	4240:4263		-23,11	-17,20	-23,30		3
cds|ENST00000360013|KALRN	ath-miR166a-3p	5047:5067		-26,32	-22,80	-21,65		3
cds|ENST00000360839|ANKHD1	ath-miR156a-5p	5949:5968		-19,19	-19,50	-19,10		3
cds|ENST00000361099|STAT1	ath-miR156a-5p	1515:1529	14		-18,20	-21,51		3
cds|ENST00000361516|FCRL2	ath-miR156a-5p	66:85		-24,66	-22,60	-23,00		3
cds|ENST00000368181|FCRL2	ath-miR156a-5p	66:85		-24,66	-22,60	-23,00		3
cds|ENST00000368571|KNDC1	ath-miR166a-3p	1994:2014		-30,50	-27,40	-27,60		3
cds|ENST00000368801|HRNR	ath-miR156a-5p	3828:3850		-23,11	-19,90	-23,01		3
cds|ENST00000368801|HRNR	ath-miR156a-5p	5238:5260		-23,11	-19,90	-23,01		3

cds|ENST00000368801|HRNR	ath-miR156a-5p	6648:6670		-23,11	-19,90	-23,01		3
cds|ENST00000369209|HSPA12A	ath-miR166a-3p	892:911		-26,35	-21,90	-22,40		3
cds|ENST00000370016|ARHGAP4	ath-miR156a-5p	911:929		-19,86	-20,50	-20,60		3
cds|ENST00000370028|ARHGAP4	ath-miR156a-5p	1094:1112		-19,86	-20,50	-20,60		3
cds|ENST00000371149|APCDD1L	ath-miR166a-3p	580:600		-25,68	-23,70	-24,01		3
cds|ENST00000373498|MAPKAP1	ath-miR166a-3p	1039:1059		-32,75	-30,60	-30,70		3
cds|ENST00000373503|MAPKAP1	ath-miR166a-3p	463:483		-32,75	-30,60	-30,70		3
cds|ENST00000373511|MAPKAP1	ath-miR166a-3p	1039:1059		-32,75	-30,60	-30,70		3
cds|ENST00000374694|FZD8	ath-miR166a-3p	1808:1821	13		-29,20	-29,50		3
cds|ENST00000375679|CLCNKB	ath-miR156a-5p	890:908		-28,42	-27,20	-27,80		3
cds|ENST00000375692|CLCNKA	ath-miR156a-5p	890:908		-32,11	-30,00	-30,10		3
cds|ENST00000376452|KIAA1217	ath-miR156a-5p	3847:3865		-19,16	-18,30	-26,61		3
cds|ENST00000376454|KIAA1217	ath-miR156a-5p	5554:5572		-19,16	-18,30	-26,61		3
cds|ENST00000376462|KIAA1217	ath-miR156a-5p	3517:3535		-19,16	-18,30	-26,61		3
cds|ENST00000376724|TRIM40	ath-miR156a-5p	93:105	12		-23,70	-24,40		3
cds|ENST00000378004|ARHGAP26	ath-miR166a-3p	494:513		-27,86	-24,00	-24,60		3
cds|ENST00000378486|PLCH2	ath-miR166a-3p	189:201	12		-23,70	-30,55		3
cds|ENST00000378858|LRRN4	ath-miR156a-5p	789:808		-21,93	-19,60	-19,80		3
cds|ENST00000380217|FRY	osa-miR168a-5p	463:483	14	-31,94		-31,10		3
cds|ENST00000380250|FRY	osa-miR168a-5p	8914:8934	14	-31,94		-31,10		3
cds|ENST00000381486|GREB1	ath-miR156a-5p	1888:1909		-28,56	-24,50	-24,40		3
cds|ENST00000382745|CLCN7	ath-miR166a-3p	15:35		-25,05	-21,80	-22,10		3
cds|ENST00000383610|TRIM40	ath-miR156a-5p	93:105	12		-23,70	-24,40		3
cds|ENST00000389758|MROH2A	ath-miR156a-5p	1525:1546		-22,91	-20,50	-22,00		3
cds|ENST00000389793|LYST	ath-miR156a-5p	214:234		-23,37	-22,20	-21,90		3
cds|ENST00000389794|LYST	ath-miR156a-5p	214:234		-23,37	-22,20	-21,90		3
cds|ENST00000391742|LAIR1	ath-miR156a-5p	514:533		-22,65	-22,50	-22,40		3
cds|ENST00000391743|LAIR1	ath-miR156a-5p	460:479		-22,65	-22,50	-22,40		3

cds|ENST00000392002|TMEM91	ath-miR156a-5p	330:350		-22,50	-22,50	-21,90		3
cds|ENST00000392322|STAT1	ath-miR156a-5p	1515:1529	14		-18,20	-21,51		3
cds|ENST00000392323|STAT1	ath-miR156a-5p	1521:1535	14		-18,20	-21,51		3
cds|ENST00000392328|MFSD6	ath-miR156a-5p	898:919		-22,53	-20,10	-20,20		3
cds|ENST00000392826|SLC4A2	ath-miR166a-3p	2401:2424		-31,16	-26,40	-26,60		3
cds|ENST00000393085|MTPN	ath-miR156a-5p	202:222		-22,11	-21,30	-23,02		3
cds|ENST00000394063|MAPKAP1	ath-miR166a-3p	463:483		-32,75	-30,60	-30,70		3
cds|ENST00000394126|MED24	ath-miR156a-5p	291:310		-23,19	-20,70	-20,70		3
cds|ENST00000394128|MED24	ath-miR156a-5p	216:235		-23,19	-20,70	-20,70		3
cds|ENST00000395757|SREBF1	ath-miR166a-3p	1554:1574		-28,21	-24,80	-25,36		3
cds|ENST00000396581|TRIM40	ath-miR156a-5p	93:105	12		-23,70	-24,40		3
cds|ENST00000397527|CEP250	ath-miR166a-3p	285:306		-26,59	-23,70	-23,04		3
cds|ENST00000398548|PRDM15	ath-miR166a-3p	1682:1701		-31,83	-28,40	-28,40		3
cds|ENST00000398612|ZNF596	ath-miR156a-5p	1279:1300		-19,16	-17,60	-19,30		3
cds|ENST00000398788|CDH23	ath-miR156a-5p	2674:2693		-20,84	-19,40	-19,80		3
cds|ENST00000400651|TRIM40	ath-miR156a-5p	93:105	12		-23,70	-24,40		3
cds|ENST00000400677|HMX1	ath-miR166a-3p	821:843		-30,79	-25,00	-28,00		3
cds|ENST00000401669|NRXN1	ath-miR156a-5p	2572:2584	12		-20,00	-19,22		3
cds|ENST00000402717|NRXN1	ath-miR156a-5p	1717:1729	12		-20,00	-19,22		3
cds|ENST00000404971|NRXN1	ath-miR156a-5p	2692:2704	12		-20,00	-19,22		3
cds|ENST00000405006|THADA	ath-miR156a-5p	5543:5561		-22,32	-22,80	-23,60		3
cds|ENST00000405472|NRXN1	ath-miR156a-5p	2536:2548	12		-20,00	-19,22		3
cds|ENST00000405975|THADA	ath-miR156a-5p	5543:5561		-22,32	-22,80	-23,60		3
cds|ENST00000406316|NRXN1	ath-miR156a-5p	2572:2584	12		-20,00	-19,22		3
cds|ENST00000406859|NRXN1	ath-miR156a-5p	1762:1774	12		-20,00	-19,22		3
cds|ENST00000409465|STAT1	ath-miR156a-5p	1515:1529	14		-18,20	-21,51		3
cds|ENST00000409552|CLEC16A	ath-miR156a-5p	929:948		-20,98	-18,90	-21,40		3
cds|ENST00000409632|ALS2	ath-miR166a-3p	43:63	12	-30,19		-31,60		3

cds|ENST00000409790|CLEC16A	ath-miR156a-5p	935:954		-20,98	-18,90	-21,40		3
cds|ENST00000410052|ALS2	ath-miR166a-3p	43:63	12	-30,19		-31,60		3
cds|ENST00000413014|TMEM91	ath-miR156a-5p	330:350		-22,50	-22,50	-21,90		3
cds|ENST00000413384|SLC4A2	ath-miR166a-3p	2428:2451		-31,16	-26,40	-26,60		3
cds|ENST00000415613|MAPT	ath-miR156a-5p	1325:1337	12		-19,90	-21,76		3
cds|ENST00000417454|GP6	ath-miR156a-5p	13:32		-21,61	-21,10	-21,60		3
cds|ENST00000418267|TRIM40	ath-miR156a-5p	93:105	12		-23,70	-24,40		3
cds|ENST00000419816|PLCH2	ath-miR166a-3p	189:201	12		-23,70	-30,55		3
cds|ENST00000420383|ARHGAP4	ath-miR156a-5p	974:992		-19,86	-20,50	-20,60		3
cds|ENST00000421981|TRIM40	ath-miR156a-5p	93:105	12		-23,70	-24,40		3
cds|ENST00000422911|PRDM15	ath-miR166a-3p	1742:1761		-31,83	-28,40	-28,40		3
cds|ENST00000422918|ARHGAP4	ath-miR156a-5p	299:317		-19,86	-20,50	-20,60		3
cds|ENST00000425845|TRIM40	ath-miR156a-5p	93:105	12		-23,70	-24,40		3
cds|ENST00000425934|CEP250	ath-miR166a-3p	285:306		-26,59	-23,70	-23,04		3
cds|ENST00000427970|GHSR	ath-miR156a-5p	664:683		-19,67	-19,40	-19,90		3
cds|ENST00000429471|TRIM40	ath-miR156a-5p	93:105	12		-23,70	-24,40		3
cds|ENST00000430453|KIAA1217	ath-miR156a-5p	3517:3535		-19,16	-18,30	-26,61		3
cds|ENST00000430485|TRIM40	ath-miR156a-5p	93:105	12		-23,70	-24,40		3
cds|ENST00000431397|TRIM40	ath-miR156a-5p	93:105	12		-23,70	-24,40		3
cds|ENST00000431508|ANKHD1	ath-miR156a-5p	1917:1936		-19,19	-19,50	-19,10		3
cds|ENST00000432692|TANK	ath-miR156a-5p	332:350		-21,70	-19,60	-20,50		3
cds|ENST00000433049|ANKHD1	ath-miR156a-5p	1515:1534		-19,19	-19,50	-19,10		3
cds|ENST00000433067|PRDM15	ath-miR166a-3p	2669:2688		-31,83	-28,40	-28,40		3
cds|ENST00000433713|TRIM40	ath-miR156a-5p	93:105	12		-23,70	-24,40		3
cds|ENST00000433971|TPRG1	ath-miR156a-5p	521:540		-23,37	-23,30	-23,30		3
cds|ENST00000434151|TRIM40	ath-miR156a-5p	93:105	12		-23,70	-24,40		3
cds|ENST00000434277|LAIR1	ath-miR156a-5p	511:530		-22,65	-22,50	-22,40		3
cds|ENST00000434758|C11orf70	ath-miR156a-5p	640:658		-20,76	-20,10	-20,60		3

cds|ENST00000436170|TMEM91	ath-miR156a-5p	330:350		-22,50	-22,50	-21,90		3
cds|ENST00000436951|TRIM40	ath-miR156a-5p	93:105	12		-23,70	-24,40		3
cds|ENST00000437563|TRIM40	ath-miR156a-5p	93:105	12		-23,70	-24,40		3
cds|ENST00000445004|TRIM40	ath-miR156a-5p	93:105	12		-23,70	-24,40		3
cds|ENST00000446248|CAMSAP3	ath-miR156a-5p	1149:1167		-20,89	-20,20	-20,80		3
cds|ENST00000447016|PRDM15	ath-miR166a-3p	1571:1590		-31,83	-28,40	-28,40		3
cds|ENST00000447207|PRDM15	ath-miR166a-3p	1571:1590		-31,83	-28,40	-28,40		3
cds|ENST00000447302|TMEM91	ath-miR156a-5p	330:350		-22,50	-22,50	-21,90		3
cds|ENST00000447611|FANCI	ath-miR156a-5p	924:945		-21,41	-23,40	-24,00		3
cds|ENST00000447648|TECPR1	ath-miR166a-3p	781:804		-31,72	-27,10	-28,00		3
cds|ENST00000448238|NIPBL	ath-miR166a-3p	657:679		-19,84	-16,80	-19,90		3
cds|ENST00000448525|CLCN7	ath-miR166a-3p	15:35		-25,05	-21,80	-22,10		3
cds|ENST00000449969|PLCH2	ath-miR166a-3p	108:120	12		-23,70	-30,55		3
cds|ENST00000451813|TRPC4AP	ath-miR166a-3p	146:160	14		-27,80	-28,00		3
cds|ENST00000454036|SLC12A5	ath-miR166a-3p	3365:3384		-23,14	-22,00	-21,70		3
cds|ENST00000456884|TRIM40	ath-miR156a-5p	93:105	12		-23,70	-24,40		3
cds|ENST00000458595|KIAA1217	ath-miR156a-5p	3772:3790		-19,16	-18,30	-26,61		3
cds|ENST00000461052|ARHGAP4	ath-miR156a-5p	905:923		-19,86	-20,50	-20,60		3
cds|ENST00000461735|SLC4A2	ath-miR166a-3p	2386:2409		-31,16	-26,40	-26,60		3
cds|ENST00000464606|ZC3HAV1	ath-miR156a-5p	2130:2152		-24,46	-21,10	-21,10		3
cds|ENST00000467448|ALS2	ath-miR166a-3p	43:63	12	-30,19		-31,60		3
cds|ENST00000471652|ZC3HAV1	ath-miR156a-5p	1764:1786		-24,46	-21,10	-21,10		3
cds|ENST00000474878|LAIR1	ath-miR156a-5p	460:479		-22,65	-22,50	-22,40		3
cds|ENST00000475390|SLITRK3	ath-miR156a-5p	952:973		-21,67	-18,30	-21,71		3
cds|ENST00000478144|PQLC1	ath-miR156a-5p	127:146		-20,45	-20,10	-21,00		3
cds|ENST00000485713|SLC4A2	ath-miR166a-3p	2428:2451		-31,16	-26,40	-26,60		3
cds|ENST00000501516|MED24	ath-miR156a-5p	216:235		-23,19	-20,70	-20,70		3
cds|ENST00000502753|TRPC7	ath-miR166a-3p	893:906	13		-22,70	-25,10		3

cds|ENST00000502798|SV2C	ath-miR156a-5p	731:744	13		-24,30	-25,00		3
cds|ENST00000503275|TRPC7	ath-miR166a-3p	893:906	13		-22,70	-25,10		3
cds|ENST00000509682|KCNQ4	ath-miR166a-3p	449:461	12		-20,90	-22,40		3
cds|ENST00000510286|DGKQ	ath-miR166a-3p	115:127	12		-24,90	-26,10		3
cds|ENST00000513104|TRPC7	ath-miR166a-3p	893:906	13		-22,70	-25,10		3
cds|ENST00000514044|ASTE1	ath-miR156a-5p	1613:1631		-27,23	-25,90	-26,50		3
cds|ENST00000514182|CSRNP1	ath-miR156a-5p	99:118		-19,54	-19,20	-23,20		3
cds|ENST00000524981|CFAP54	ath-miR156a-5p	6832:6852		-19,28	-19,60	-20,20		3
cds|ENST00000526781|C11orf70	ath-miR156a-5p	640:658		-20,76	-20,10	-20,60		3
cds|ENST00000532219|ANKHD1-EIF4EBP3	ath-miR156a-5p	5949:5968		-19,19	-19,50	-19,10		3
cds|ENST00000535071|MED24	ath-miR156a-5p	66:85		-23,19	-20,70	-20,70		3
cds|ENST00000535508|MED24	ath-miR156a-5p	216:235		-23,19	-20,70	-20,70		3
cds|ENST00000536375|GTF2H3	ath-miR156a-5p	626:644		-21,72	-20,70	-21,10		3
cds|ENST00000537121|ADCY2	ath-miR156a-5p	1274:1286	12		-26,20	-28,80		3
cds|ENST00000537674|MED24	ath-miR156a-5p	291:310		-23,19	-20,70	-20,70		3
cds|ENST00000539627|TMEM91	ath-miR156a-5p	330:350		-22,50	-22,50	-21,90		3
cds|ENST00000540768|NUP85	ath-miR156a-5p	476:495		-22,68	-22,10	-22,50		3
cds|ENST00000541036|TIAM1	ath-miR156a-5p	304:325		-24,09	-20,40	-22,80		3
cds|ENST00000542859|FRY	osa-miR168a-5p	8917:8937	14	-31,94		-31,10		3
cds|ENST00000542945|TMEM91	ath-miR156a-5p	330:350		-22,50	-22,50	-21,90		3
cds|ENST00000543154|GTF2H3	ath-miR156a-5p	413:431		-21,72	-20,70	-21,10		3
cds|ENST00000543341|GTF2H3	ath-miR156a-5p	755:773		-21,72	-20,70	-21,10		3
cds|ENST00000544232|TMEM91	ath-miR156a-5p	330:350		-22,50	-22,50	-21,90		3
cds|ENST00000545638|NCKAP1L	ath-miR166a-3p	1085:1107		-32,64	-23,60	-26,70		3
cds|ENST00000545638|NCKAP1L	ath-miR156a-5p	268:288		-21,84	-19,00	-19,30		3
cds|ENST00000547289|TRIM40	ath-miR156a-5p	93:105	12		-23,70	-24,40		3
cds|ENST00000548221|NCKAP1L	ath-miR166a-3p	1235:1257		-32,64	-23,60	-26,70		3

cds|ENST00000548672|TRIM40	ath-miR156a-5p	93:105	12		-23,70	-24,40		3
cds|ENST00000549728|TRIM40	ath-miR156a-5p	93:105	12		-23,70	-24,40		3
cds|ENST00000552119|TRIM40	ath-miR156a-5p	93:105	12		-23,70	-24,40		3
cds|ENST00000571987|MAPT	ath-miR156a-5p	1325:1337	12		-19,90	-21,76		3
cds|ENST00000575270|NFATC3	ath-miR156a-5p	928:946		-21,30	-20,50	-21,40		3
cds|ENST00000578161|MED24	ath-miR156a-5p	291:310		-23,19	-20,70	-20,70		3
cds|ENST00000579298|NUP85	ath-miR156a-5p	1532:1551		-22,68	-22,10	-22,50		3
cds|ENST00000579324|NUP85	ath-miR156a-5p	1331:1350		-22,68	-22,10	-22,50		3
cds|ENST00000579900|NUP85	ath-miR156a-5p	86:105		-22,68	-22,10	-22,50		3
cds|ENST00000582023|MED24	ath-miR156a-5p	354:373		-23,19	-20,70	-20,70		3
cds|ENST00000585306|MED24	ath-miR156a-5p	216:235		-23,19	-20,70	-20,70		3
cds|ENST00000585894|FAM134C	ath-miR156a-5p	496:515		-20,25	-23,20	-23,10		3
cds|ENST00000588868|STAT5A	ath-miR166a-3p	343:363		-24,22	-22,90	-22,40		3
cds|ENST00000590949|STAT5A	ath-miR166a-3p	343:363		-24,22	-22,90	-22,40		3
cds|ENST00000591587|NAGLU	ath-miR166a-3p	46:58	12		-24,80	-24,80		3
cds|ENST00000591598|CTDP1	ath-miR166a-3p	1573:1593		-27,07	-23,10	-23,90		3
cds|ENST00000598234|CTD-3088G3.8	ath-miR166a-3p	7884:7897	13		-25,10	-26,90		3
cds|ENST00000604123|TMEM91	ath-miR156a-5p	501:521		-22,50	-22,50	-21,90		3
cds|ENST00000609981|PLCH2	ath-miR166a-3p	180:192	12		-23,70	-30,55		3
cds|ENST00000610549|LAIR1	ath-miR156a-5p	460:479		-22,65	-22,50	-22,40		3
cds|ENST00000610596|GP6	ath-miR156a-5p	13:32		-21,61	-21,10	-21,60		3
cds|ENST00000610719|CPEB1	ath-miR156a-5p	1556:1569	13		-23,60	-23,30		3
cds|ENST00000610746|GP6	ath-miR156a-5p	13:32		-21,61	-21,10	-21,60		3
cds|ENST00000610772|MROH2A	ath-miR156a-5p	1534:1555		-22,91	-20,50	-22,00		3
cds|ENST00000611031|CPEB1	ath-miR156a-5p	1556:1569	13		-23,60	-23,30		3
cds|ENST00000611163|CPEB1	ath-miR156a-5p	1331:1344	13		-23,60	-23,30		3
cds|ENST00000611304|LAIR1	ath-miR156a-5p	481:500		-22,65	-22,50	-22,40		3

cds|ENST00000611436|GP6	ath-miR156a-5p	13:32		-21,61	-21,10	-21,60		3
cds|ENST00000612096|GP6	ath-miR156a-5p	13:32		-21,61	-21,10	-21,60		3
cds|ENST00000612184|GP6	ath-miR156a-5p	13:32		-21,61	-21,10	-21,60		3
cds|ENST00000612535|MILR1	ath-miR156a-5p	38:53	15		-23,30	-23,30		3
cds|ENST00000612862|GP6	ath-miR156a-5p	13:32		-21,61	-21,10	-21,60		3
cds|ENST00000613122|CTDP1	ath-miR166a-3p	1777:1797		-27,07	-23,10	-23,90		3
cds|ENST00000613311|LAIR1	ath-miR156a-5p	511:530		-22,65	-22,50	-22,40		3
cds|ENST00000613649|GP6	ath-miR156a-5p	13:32		-21,61	-21,10	-21,60		3
cds|ENST00000613801|GP6	ath-miR156a-5p	13:32		-21,61	-21,10	-21,60		3
cds|ENST00000614384|MED24	ath-miR156a-5p	216:235		-23,19	-20,70	-20,70		3
cds|ENST00000614405|TRIM40	ath-miR156a-5p	93:105	12		-23,70	-24,40		3
cds|ENST00000614662|GP6	ath-miR156a-5p	13:32		-21,61	-21,10	-21,60		3
cds|ENST00000614889|GP6	ath-miR156a-5p	13:32		-21,61	-21,10	-21,60		3
cds|ENST00000614918|CPEB1	ath-miR156a-5p	1637:1650	13		-23,60	-23,30		3
cds|ENST00000614937|LAIR1	ath-miR156a-5p	460:479		-22,65	-22,50	-22,40		3
cds|ENST00000614946|LAIR1	ath-miR156a-5p	460:479		-22,65	-22,50	-22,40		3
cds|ENST00000615198|CPEB1	ath-miR156a-5p	1556:1569	13		-23,60	-23,30		3
cds|ENST00000615220|MILR1	ath-miR156a-5p	38:53	15		-23,30	-23,30		3
cds|ENST00000615407|GP6	ath-miR156a-5p	13:32		-21,61	-21,10	-21,60		3
cds|ENST00000616319|GP6	ath-miR156a-5p	13:32		-21,61	-21,10	-21,60		3
cds|ENST00000616332|TRIM61	ath-miR156a-5p	313:327	14		-22,60	-22,70		3
cds|ENST00000616351|TRIM40	ath-miR156a-5p	93:105	12		-23,70	-24,40		3
cds|ENST00000616413|LAIR1	ath-miR156a-5p	514:533		-22,65	-22,50	-22,40		3
cds|ENST00000616456|GP6	ath-miR156a-5p	13:32		-21,61	-21,10	-21,60		3
cds|ENST00000616498|MILR1	ath-miR156a-5p	38:53	15		-23,30	-23,30		3
cds|ENST00000616612|LAIR1	ath-miR156a-5p	514:533		-22,65	-22,50	-22,40		3
cds|ENST00000616724|LAIR1	ath-miR156a-5p	460:479		-22,65	-22,50	-22,40		3
cds|ENST00000616775|CPEB1	ath-miR156a-5p	1346:1359	13		-23,60	-23,30		3

cds|ENST00000616959|CPEB1	ath-miR156a-5p	1565:1578	13		-23,60	-23,30		3
cds|ENST00000617029|LAIR1	ath-miR156a-5p	511:530		-22,65	-22,50	-22,40		3
cds|ENST00000617071|MILR1	ath-miR156a-5p	38:53	15		-23,30	-23,30		3
cds|ENST00000617386|LAIR1	ath-miR156a-5p	511:530		-22,65	-22,50	-22,40		3
cds|ENST00000617462|CPEB1	ath-miR156a-5p	1331:1344	13		-23,60	-23,30		3
cds|ENST00000617522|CPEB1	ath-miR156a-5p	1331:1344	13		-23,60	-23,30		3
cds|ENST00000617954|GP6	ath-miR156a-5p	13:32		-21,61	-21,10	-21,60		3
cds|ENST00000617958|CPEB1	ath-miR156a-5p	1751:1764	13		-23,60	-23,30		3
cds|ENST00000618029|MAPT	ath-miR156a-5p	1325:1337	12		-22,30	-22,90		3
cds|ENST00000618160|GTF2H3	ath-miR156a-5p	317:335		-21,72	-20,70	-21,10		3
cds|ENST00000618291|LAIR1	ath-miR156a-5p	460:479		-22,65	-22,50	-22,40		3
cds|ENST00000618434|LAIR1	ath-miR156a-5p	514:533		-22,65	-22,50	-22,40		3
cds|ENST00000618449|CPEB1	ath-miR156a-5p	1346:1359	13		-23,60	-23,30		3
cds|ENST00000618825|MAPT	ath-miR156a-5p	1325:1337	12		-22,30	-22,90		3
cds|ENST00000618891|TRIM40	ath-miR156a-5p	93:105	12		-23,70	-24,40		3
cds|ENST00000619136|GP6	ath-miR156a-5p	13:32		-21,61	-21,10	-21,60		3
cds|ENST00000619286|MILR1	ath-miR156a-5p	38:53	15		-23,30	-23,30		3
cds|ENST00000619696|CPEB1	ath-miR156a-5p	1331:1344	13		-23,60	-23,30		3
cds|ENST00000619717|LAIR1	ath-miR156a-5p	514:533		-22,65	-22,50	-22,40		3
cds|ENST00000619817|LAIR1	ath-miR156a-5p	463:482		-22,65	-22,50	-22,40		3
cds|ENST00000619887|CDH23	ath-miR156a-5p	2674:2693		-20,84	-19,40	-19,80		3
cds|ENST00000619905|PPP4R4	ath-miR156a-5p	2253:2272		-20,52	-18,80	-21,80		3
cds|ENST00000620077|LAIR1	ath-miR156a-5p	463:482		-22,65	-22,50	-22,40		3
cds|ENST00000620182|CPEB1	ath-miR156a-5p	1331:1344	13		-23,60	-23,30		3
cds|ENST00000620212|CPEB1	ath-miR156a-5p	1331:1344	13		-23,60	-23,30		3
cds|ENST00000620357|LAIR1	ath-miR156a-5p	463:482		-22,65	-22,50	-22,40		3
cds|ENST00000620687|PLCH2	ath-miR166a-3p	189:201	12		-23,70	-30,55		3
cds|ENST00000620726|LAIR1	ath-miR156a-5p	460:479		-22,65	-22,50	-22,40		3

cds|ENST00000621278|GP6	ath-miR156a-5p	13:32		-21,61	-21,10	-21,60		3
cds|ENST00000621291|LAIR1	ath-miR156a-5p	460:479		-22,65	-22,50	-22,40		3
cds|ENST00000621769|LAIR1	ath-miR156a-5p	460:479		-22,65	-22,50	-22,40		3
cds|ENST00000622097|LAIR1	ath-miR156a-5p	463:482		-22,65	-22,50	-22,40		3
cds|ENST00000622279|GP6	ath-miR156a-5p	13:32		-21,61	-21,10	-21,60		3
cds|ENST00000622589|GP6	ath-miR156a-5p	13:32		-21,61	-21,10	-21,60		3
cds|ENST00000622653|GP6	ath-miR156a-5p	13:32		-21,61	-21,10	-21,60		3
cds|ENST00000622827|CDH23	ath-miR156a-5p	9394:9413		-20,84	-19,40	-19,80		3
cds|ENST00000625672|NRXN1	ath-miR156a-5p	2548:2560	12		-20,00	-19,22		3
cds|ENST00000626246|PLCH2	ath-miR166a-3p	189:201	12		-23,70	-30,55		3
cds|ENST00000627711|MAPT	ath-miR156a-5p	1325:1337	12		-19,90	-21,76		3
cds|ENST00000627854|PLCH2	ath-miR166a-3p	108:120	12		-23,70	-30,55		3
cds|ENST00000629368|MAPT	ath-miR156a-5p	1325:1337	12		-19,90	-21,76		3
cds|ENST00000630543|NRXN1	ath-miR156a-5p	2548:2560	12		-20,00	-19,22		3
cds|ENST00000631674|CPEB1	ath-miR156a-5p	1331:1344	13		-23,60	-23,30		3
cds|ENST00000632172|CPEB1	ath-miR156a-5p	1751:1764	13		-23,60	-23,30		3
cds|ENST00000632301|CTDP1	ath-miR166a-3p	1777:1797		-27,07	-23,10	-23,90		3
cds|ENST00000632305|CPEB1	ath-miR156a-5p	1541:1554	13		-23,60	-23,30		3
cds|ENST00000632450|CTDP1	ath-miR166a-3p	1573:1593		-27,07	-23,10	-23,90		3
cds|ENST00000632526|CPEB1	ath-miR156a-5p	1331:1344	13		-23,60	-23,30		3
cds|ENST00000632932|CTDP1	ath-miR166a-3p	1777:1797		-27,07	-23,10	-23,90		3
cds|ENST00000633054|MAPT	ath-miR156a-5p	1325:1337	12		-22,30	-22,90		3
cds|ENST00000633293|CPEB1	ath-miR156a-5p	1565:1578	13		-23,60	-23,30		3
cds|ENST00000633801|MAPT	ath-miR156a-5p	1325:1337	12		-22,30	-22,90		3
3utr|ENST00000171887|TNS1	ath-miR156a-5p	2841:2860		-22,85	-21,40	-22,00	-20,00	4
3utr|ENST00000173229|NTN1	ath-miR156a-5p	3211:3229		-21,00	-20,80	-21,00	-19,80	4
3utr|ENST00000192788|UHRF1BP1	ath-miR156a-5p	869:890		-23,91	-21,60	-23,40	-22,20	4
3utr|ENST00000219343|SLC7A6	ath-miR156a-5p	1132:1152		-23,43	-23,20	-23,90	-21,40	4

3utr|ENST00000219596|MEFV	ath-miR156a-5p	348:368		-24,83	-24,20	-24,60	-22,70	4
3utr|ENST00000223208|CEP41	ath-miR156a-5p	4222:4241		-24,42	-21,70	-22,20	-19,50	4
5utr|ENST00000320717|GLS	ath-miR156a-5p	140:160		-21,52	-19,50	-20,20	-19,10	4
5utr|ENST00000338435|GLS	ath-miR156a-5p	133:153		-21,52	-19,50	-20,20	-19,10	4
5utr|ENST00000341947|COL11A2	ath-miR156a-5p	142:161		-27,57	-26,50	-26,30	-25,60	4
5utr|ENST00000360461|PLEKHG4	ath-miR156a-5p	114:133		-24,04	-23,70	-24,30	-19,40	4
5utr|ENST00000361917|COL11A2	ath-miR156a-5p	142:161		-27,57	-26,50	-26,30	-25,60	4
5utr|ENST00000374708|COL11A2	ath-miR156a-5p	173:192		-27,57	-26,50	-26,30	-25,60	4
5utr|ENST00000383087|COL11A2	ath-miR156a-5p	173:192		-27,57	-26,50	-26,30	-25,60	4
5utr|ENST00000383088|COL11A2	ath-miR156a-5p	128:147		-27,57	-26,50	-26,30	-25,60	4
5utr|ENST00000383219|COL11A2	ath-miR156a-5p	173:192		-27,57	-26,50	-26,30	-25,60	4
5utr|ENST00000395194|COL11A2	ath-miR156a-5p	128:147		-27,57	-26,50	-26,30	-25,60	4
5utr|ENST00000420405|COL11A2	ath-miR156a-5p	141:160		-27,57	-26,50	-26,30	-25,60	4
5utr|ENST00000425729|COL11A2	ath-miR156a-5p	173:192		-27,57	-26,50	-26,30	-25,60	4
5utr|ENST00000435763|COL11A2	ath-miR156a-5p	128:147		-27,57	-26,50	-26,30	-25,60	4
5utr|ENST00000438711|COL11A2	ath-miR156a-5p	141:160		-27,57	-26,50	-26,30	-25,60	4
5utr|ENST00000439039|COL11A2	ath-miR156a-5p	128:147		-27,57	-26,50	-26,30	-25,60	4
5utr|ENST00000447349|COL11A2	ath-miR156a-5p	173:192		-27,57	-26,50	-26,30	-25,60	4
5utr|ENST00000447741|COL11A2	ath-miR156a-5p	128:147		-27,57	-26,50	-26,30	-25,60	4
5utr|ENST00000448717|COL11A2	ath-miR156a-5p	173:192		-27,57	-26,50	-26,30	-25,60	4
5utr|ENST00000451040|COL11A2	ath-miR156a-5p	141:160		-27,57	-26,50	-26,30	-25,60	4
5utr|ENST00000452044|COL11A2	ath-miR156a-5p	141:160		-27,57	-26,50	-26,30	-25,60	4
5utr|ENST00000452730|COL11A2	ath-miR156a-5p	141:160		-27,57	-26,50	-26,30	-25,60	4
5utr|ENST00000452937|COL11A2	ath-miR156a-5p	128:147		-27,57	-26,50	-26,30	-25,60	4
5utr|ENST00000457788|COL11A2	ath-miR156a-5p	141:160		-27,57	-26,50	-26,30	-25,60	4
5utr|ENST00000468137|ABTB1	ath-miR156a-5p	16:37		-21,27	-20,60	-20,10	-19,10	4
5utr|ENST00000508079|WDR1	ath-miR156a-5p	32:53		-22,75	-22,40	-22,70	-20,20	4
5utr|ENST00000536384|SERINC2	ath-miR156a-5p	51:70		-19,62	-20,40	-22,00	-19,20	4

5utr|ENST00000549491|COL11A2	ath-miR156a-5p	142:161		-27,57	-26,50	-26,30	-25,60	4
5utr|ENST00000549811|COL11A2	ath-miR156a-5p	142:161		-27,57	-26,50	-26,30	-25,60	4
5utr|ENST00000549836|COL11A2	ath-miR156a-5p	142:161		-27,57	-26,50	-26,30	-25,60	4
5utr|ENST00000549885|COL11A2	ath-miR156a-5p	142:161		-27,57	-26,50	-26,30	-25,60	4
5utr|ENST00000550998|COL11A2	ath-miR156a-5p	142:161		-27,57	-26,50	-26,30	-25,60	4
5utr|ENST00000551413|COL11A2	ath-miR156a-5p	142:161		-27,57	-26,50	-26,30	-25,60	4
5utr|ENST00000551542|COL11A2	ath-miR156a-5p	142:161		-27,57	-26,50	-26,30	-25,60	4
5utr|ENST00000551758|COL11A2	ath-miR156a-5p	142:161		-27,57	-26,50	-26,30	-25,60	4
5utr|ENST00000552134|COL11A2	ath-miR156a-5p	142:161		-27,57	-26,50	-26,30	-25,60	4
5utr|ENST00000552473|COL11A2	ath-miR156a-5p	142:161		-27,57	-26,50	-26,30	-25,60	4
5utr|ENST00000563969|PLEKHG4	ath-miR156a-5p	114:133		-24,04	-23,70	-24,30	-19,40	4
5utr|ENST00000571253|GABARAP	ath-miR156a-5p	531:550		-23,74	-22,80	-23,10	-20,20	4
5utr|ENST00000593844|DAPK3	ath-miR156a-5p	74:92		-22,27	-21,40	-22,30	-19,20	4
cds|ENST00000220616|TG	ath-miR156a-5p	5156:5174		-30,67	-29,70	-29,90	-27,90	4

Supplementary Table S2. Results of the KEGG pathway mapping analysis for predicted plant miRNA targets. The KEGG pathway mapping was performed by the KAAS service. The human targets predicted for 5 evaluated plant miRNAs were used.

KEGG pathway ID and name	# of targets in pathway
ko01100 Metabolic pathways	24
ko05200 Pathways in cancer	12
ko00230 Purine metabolism	9
ko05166 HTLV-I infection	8
ko03013 RNA transport	7
ko04919 Thyroid hormone signaling pathway	7
ko04120 Ubiquitin mediated proteolysis	6
ko04390 Hippo signaling pathway	6
ko04020 Calcium signaling pathway	6
ko04658 Th1 and Th2 cell differentiation	6
ko04062 Chemokine signaling pathway	6
ko04914 Progesterone-mediated oocyte maturation	6
ko04727 GABAergic synapse	6
ko01110 Biosynthesis of secondary metabolites	5
ko04141 Protein processing in endoplasmic reticulum	5
ko04072 Phospholipase D signaling pathway	5
ko04024 cAMP signaling pathway	5
ko04022 cGMP-PKG signaling pathway	5
ko04512 ECM-receptor interaction	5
ko04810 Regulation of actin cytoskeleton	5
ko04114 Oocyte meiosis	5
ko04659 Th17 cell differentiation	5
ko04724 Glutamatergic synapse	5
ko05206 MicroRNAs in cancer	5
ko05203 Viral carcinogenesis	5
ko05012 Parkinson's disease	5
ko05032 Morphine addiction	5
ko05162 Measles	5
ko04015 Rap1 signaling pathway	4
ko04630 Jak-STAT signaling pathway	4
ko04151 PI3K-Akt signaling pathway	4
ko04150 mTOR signaling pathway	4
ko04080 Neuroactive ligand-receptor interaction	4
ko04142 Lysosome	4
ko04111 Cell cycle - yeast	4

ko04611 Platelet activation	4
ko04621 NOD-like receptor signaling pathway	4
ko04261 Adrenergic signaling in cardiomyocytes	4
ko04750 Inflammatory mediator regulation of TRP channels	4
ko05010 Alzheimer's disease	4
ko05016 Huntington's disease	4
ko05414 Dilated cardiomyopathy	4
ko04932 Non-alcoholic fatty liver disease NAFLD	4
ko04933 AGE-RAGE signaling pathway in diabetic complications	4
ko05161 Hepatitis B	4
ko01522 Endocrine resistance	4
ko00240 Pyrimidine metabolism	3
ko03015 mRNA surveillance pathway	3
ko03008 Ribosome biogenesis in eukaryotes	3
ko04014 Ras signaling pathway	3
ko04010 MAPK signaling pathway	3
ko04012 ErbB signaling pathway	3
ko04310 Wnt signaling pathway	3
ko04152 AMPK signaling pathway	3
ko04514 Cell adhesion molecules CAMs	3
ko04110 Cell cycle	3
ko04210 Apoptosis	3
ko04510 Focal adhesion	3
ko04530 Tight junction	3
ko04540 Gap junction	3
ko04640 Hematopoietic cell lineage	3
ko04922 Glucagon signaling pathway	3
ko04917 Prolactin signaling pathway	3
ko04921 Oxytocin signaling pathway	3
ko04918 Thyroid hormone synthesis	3
ko04916 Melanogenesis	3
ko04970 Salivary secretion	3
ko04971 Gastric acid secretion	3
ko04972 Pancreatic secretion	3
ko04976 Bile secretion	3
ko04725 Cholinergic synapse	3
ko04723 Retrograde endocannabinoid signaling	3
ko04740 Olfactory transduction	3
ko04360 Axon guidance	3
ko05205 Proteoglycans in cancer	3
ko05221 Acute myeloid leukemia	3
ko05412 Arrhythmogenic right ventricular cardiomyopathy ARVC	3

ko04931 Insulin resistance	3
ko05110 Vibrio cholerae infection	3
ko05152 Tuberculosis	3
ko01130 Biosynthesis of antibiotics	2
ko00052 Galactose metabolism	2
ko00562 Inositol phosphate metabolism	2
ko00190 Oxidative phosphorylation	2
ko00250 Alanine aspartate and glutamate metabolism	2
ko00330 Arginine and proline metabolism	2
ko00510 N-Glycan biosynthesis	2
ko00513 Various types of N-glycan biosynthesis	2
ko00601 Glycosphingolipid biosynthesis - lacto and neolacto series	2
ko00603 Glycosphingolipid biosynthesis - globo and isoglobo series	2
ko02010 ABC transporters	2
ko04070 Phosphatidylinositol signaling system	2
ko04071 Sphingolipid signaling pathway	2
ko04060 Cytokine-cytokine receptor interaction	2
ko04144 Endocytosis	2
ko04145 Phagosome	2
ko04146 Peroxisome	2
ko04660 T cell receptor signaling pathway	2
ko04662 B cell receptor signaling pathway	2
ko04670 Leukocyte transendothelial migration	2
ko04911 Insulin secretion	2
ko04910 Insulin signaling pathway	2
ko04923 Regulation of lipolysis in adipocytes	2
ko04912 GnRH signaling pathway	2
ko04913 Ovarian steroidogenesis	2
ko04915 Estrogen signaling pathway	2
ko04924 Renin secretion	2
ko04614 Renin-angiotensin system	2
ko04925 Aldosterone synthesis and secretion	2
ko04270 Vascular smooth muscle contraction	2
ko04974 Protein digestion and absorption	2
ko04726 Serotonergic synapse	2
ko04720 Long-term potentiation	2
ko04722 Neurotrophin signaling pathway	2
ko04742 Taste transduction	2
ko04320 Dorso-ventral axis formation	2
ko04211 Longevity regulating pathway	2
ko04713 Circadian entrainment	2
ko05231 Choline metabolism in cancer	2

ko05220 Chronic myeloid leukemia	2
ko05224 Breast cancer	2
ko05222 Small cell lung cancer	2
ko05322 Systemic lupus erythematosus	2
ko05034 Alcoholism	2
ko05410 Hypertrophic cardiomyopathy HCM	2
ko05120 Epithelial cell signaling in Helicobacter pylori infection	2
ko05164 Influenza A	2
ko05160 Hepatitis C	2
ko05168 Herpes simplex infection	2
ko05169 Epstein-Barr virus infection	2
ko05146 Amoebiasis	2
ko05145 Toxoplasmosis	2
ko05142 Chagas disease American trypanosomiasis	2
ko01120 Microbial metabolism in diverse environments	1
ko00051 Fructose and mannose metabolism	1
ko00500 Starch and sucrose metabolism	1
ko00520 Amino sugar and nucleotide sugar metabolism	1
ko00620 Pyruvate metabolism	1
ko00640 Propanoate metabolism	1
ko00061 Fatty acid biosynthesis	1
ko00561 Glycerolipid metabolism	1
ko00564 Glycerophospholipid metabolism	1
ko00590 Arachidonic acid metabolism	1
ko00310 Lysine degradation	1
ko00220 Arginine biosynthesis	1
ko00340 Histidine metabolism	1
ko00410 beta-Alanine metabolism	1
ko00471 D-Glutamine and D-glutamate metabolism	1
ko00515 Mannose type O-glycan biosynthesis	1
ko00514 Other types of O-glycan biosynthesis	1
ko00532 Glycosaminoglycan biosynthesis - chondroitin sulfate / dermatan sulfate	1
ko00534 Glycosaminoglycan biosynthesis - heparan sulfate / heparin	1
ko00533 Glycosaminoglycan biosynthesis - keratan sulfate	1
ko00531 Glycosaminoglycan degradation	1
ko00511 Other glycan degradation	1
ko00760 Nicotinate and nicotinamide metabolism	1
ko00232 Caffeine metabolism	1
ko00254 Aflatoxin biosynthesis	1
ko00983 Drug metabolism - other enzymes	1
ko03020 RNA polymerase	1
ko03022 Basal transcription factors	1

ko03040 Spliceosome	1
ko03060 Protein export	1
ko03410 Base excision repair	1
ko03420 Nucleotide excision repair	1
ko03460 Fanconi anemia pathway	1
ko04330 Notch signaling pathway	1
ko04350 TGF-beta signaling pathway	1
ko04370 VEGF signaling pathway	1
ko04064 NF-kappa B signaling pathway	1
ko04668 TNF signaling pathway	1
ko04066 HIF-1 signaling pathway	1
ko04068 FoxO signaling pathway	1
ko04140 Autophagy	1
ko04115 p53 signaling pathway	1
ko04520 Adherens junction	1
ko04550 Signaling pathways regulating pluripotency of stem cells	1
ko04620 Toll-like receptor signaling pathway	1
ko04622 RIG-I-like receptor signaling pathway	1
ko04623 Cytosolic DNA-sensing pathway	1
ko04650 Natural killer cell mediated cytotoxicity	1
ko04664 Fc epsilon RI signaling pathway	1
ko04666 Fc gamma R-mediated phagocytosis	1
ko04920 Adipocytokine signaling pathway	1
ko03320 PPAR signaling pathway	1
ko04973 Carbohydrate digestion and absorption	1
ko04978 Mineral absorption	1
ko04960 Aldosterone-regulated sodium reabsorption	1
ko04964 Proximal tubule bicarbonate reclamation	1
ko04966 Collecting duct acid secretion	1
ko04728 Dopaminergic synapse	1
ko04380 Osteoclast differentiation	1
ko05230 Central carbon metabolism in cancer	1
ko05202 Transcriptional misregulation in cancer	1
ko05212 Pancreatic cancer	1
ko05214 Glioma	1
ko05217 Basal cell carcinoma	1
ko05219 Bladder cancer	1
ko05213 Endometrial cancer	1
ko05223 Non-small cell lung cancer	1
ko05320 Autoimmune thyroid disease	1
ko05321 Inflammatory bowel disease IBD	1
ko05014 Amyotrophic lateral sclerosis ALS	1

ko05030 Cocaine addiction	1
ko05033 Nicotine addiction	1
ko05100 Bacterial invasion of epithelial cells	1
ko05140 Leishmaniasis	1
ko05143 African trypanosomiasis	1
ko01521 EGFR tyrosine kinase inhibitor resistance	1
ko01523 Antifolate resistance	1

Supplementary Table S3. Results of the GO terms annotation for predicted plant miRNAs targets. The GO terms ("Biological Process" category) annotation was performed by the Blast2GO software. The human targets predicted for 5 evaluated plant miRNAs were used.

Target Association Name	Annotated GO terms
ZNF550	GO:0005634, GO:0003677, GO:0005515, GO:0046872, GO:0006351, GO:0006355
LIME1	GO:0005615, GO:0016021, GO:0019815, GO:0019901, GO:0002250, GO:0006357, GO:0014066, GO:0042345, GO:0043405, GO:0050852, GO:0050853, GO:0051279
ZNF596	GO:0005634, GO:0003677, GO:0046872, GO:0006351, GO:0006355
SP4	GO:0005654, GO:0005737, GO:0015630, GO:0045171, GO:0000981, GO:0003677, GO:0003713, GO:0005515, GO:0046872, GO:0006351, GO:0006357
APCDD1L	GO:0016021
YPEL3	GO:0005730, GO:0046872, GO:0006915, GO:2000774
MAS1	GO:0005622, GO:0005887, GO:0009986, GO:0004945, GO:0005515, GO:0017046, GO:0001933, GO:0007250, GO:0007283, GO:0008283, GO:0008284, GO:0008584, GO:0009653, GO:0014823, GO:0021766, GO:0034698, GO:0038166, GO:0042493, GO:0045740, GO:0050727, GO:0060732, GO:0070528
GTF2H3	GO:0000439, GO:0005675, GO:0003684, GO:0003700, GO:0008094, GO:0008135, GO:0008353, GO:0046872, GO:0047485, GO:0000717, GO:0006283, GO:0006293, GO:0006294, GO:0006295, GO:0006296, GO:0006361, GO:0006362, GO:0006363, GO:0006367, GO:0006368, GO:0006370, GO:0006412, GO:0070816, GO:0070911
CD22	GO:0005886, GO:0016021, GO:0070062, GO:0005515, GO:0030246, GO:0007155
SPATA13	GO:0005654, GO:0005737, GO:0030027, GO:0030175, GO:0032587, GO:0005515, GO:0030676, GO:0016477, GO:0030032, GO:0030334, GO:0035023, GO:0043547, GO:0046847
ZNF224	GO:0017053, GO:0031965, GO:0003677, GO:0003700, GO:0005515, GO:0046872, GO:0006351, GO:0045892
CLCN7	GO:0005765, GO:0016021, GO:0031410, GO:0005247, GO:0005524, GO:0015297, GO:0009268, GO:1902476, GO:1903959
SLC12A3	GO:0005829, GO:0005887, GO:0016324, GO:0070062, GO:0015378, GO:0019899, GO:0035725, GO:1902476
SERINC2	GO:0016021, GO:0070062, GO:0015194, GO:0006658, GO:0006665, GO:0015825, GO:1904219, GO:1904222
TSPYL6	GO:0005634, GO:0006334
NRXN1	GO:0005783, GO:0005887, GO:0009986, GO:0030054, GO:0031965, GO:0031982, GO:0042734, GO:0043025, GO:0043234, GO:0004872, GO:0005246, GO:0005509, GO:0033130, GO:0050839, GO:0097109, GO:0007158, GO:0007269, GO:0007411, GO:0030534, GO:0035176, GO:0042297, GO:0050885, GO:0051965, GO:0051968, GO:0060134, GO:0061178, GO:0071625, GO:0090129, GO:0097116, GO:0097118, GO:0097119, GO:2000463, GO:2000821
TCTN1	GO:0005615, GO:0005829, GO:0005856, GO:0016020, GO:0036038, GO:0001701, GO:0001841, GO:0008589, GO:0021523, GO:0021537, GO:0021904, GO:0021956, GO:0060271
SLC26A1	GO:0005887, GO:0005254, GO:0008271, GO:0015106, GO:0015301, GO:0019531, GO:0015701, GO:0019532, GO:0042391, GO:0050428, GO:0051453,

	GO:1902358, GO:1902476
BSN	GO:0005634, GO:0005802, GO:0009986, GO:0014069, GO:0030054, GO:0030424, GO:0030425, GO:0043025, GO:0044306, GO:0048788, GO:0060077, GO:0097470, GO:0046872, GO:0007268, GO:0007416
HMX1	GO:0005634, GO:0000977, GO:0001227, GO:0000122, GO:0006351, GO:0007275
ASTE1	GO:0004518, GO:0005515, GO:0006281, GO:0090305
KAT7	GO:0000123, GO:0005730, GO:0005737, GO:0003700, GO:0004402, GO:0005515, GO:0008270, GO:0006260, GO:0006351, GO:0006355, GO:0043966, GO:0043981, GO:0043982, GO:0043983, GO:0043984, GO:1900182
AK9	GO:0005654, GO:0005829, GO:0031965, GO:0004550, GO:0005524, GO:0019206, GO:0050145, GO:0006174, GO:0006186, GO:0006756, GO:0006757, GO:0015949, GO:0061508, GO:0061565, GO:0061566, GO:0061567, GO:0061568, GO:0061569, GO:0061570, GO:0061571
SAT1	GO:0005829, GO:0004145, GO:0005515, GO:0001525, GO:0006596, GO:0009447, GO:0042127
AP3M2	GO:0005794, GO:0030131, GO:0030659, GO:1904115, GO:0006886, GO:0048490
FBXO17	GO:0019005, GO:0001948, GO:0005515
LMCD1	GO:0005615, GO:0005654, GO:0005737, GO:0031012, GO:0003714, GO:0008270, GO:0000122, GO:0002230, GO:0006351, GO:0010611, GO:0044267, GO:0070886, GO:0098779, GO:0098792
CLCNKA	GO:0005887, GO:0034707, GO:0005247, GO:0046872, GO:0007588, GO:1902476, GO:1903959
NRXN1	GO:0005783, GO:0005887, GO:0009986, GO:0030054, GO:0030139, GO:0031965, GO:0042734, GO:0043025, GO:0043234, GO:0044295, GO:0004888, GO:0005246, GO:0005509, GO:0033130, GO:0048306, GO:0050839, GO:0097109, GO:0001525, GO:0007157, GO:0007158, GO:0007269, GO:0007411, GO:0016339, GO:0021707, GO:0023041, GO:0030534, GO:0035176, GO:0042297, GO:0045184, GO:0050885, GO:0051490, GO:0051965, GO:0051968, GO:0060134, GO:0061178, GO:0071625, GO:0090004, GO:0090126, GO:0090129, GO:0097091, GO:0097105, GO:0097112, GO:0097114, GO:0097116, GO:0097117, GO:0097118, GO:0097119, GO:0097120, GO:2000310, GO:2000311, GO:2000463, GO:2000821
XKRX	GO:0005886, GO:0016021
BNC2	GO:0005634, GO:0005737, GO:0005886, GO:0003677, GO:0046872, GO:0003416, GO:0006351, GO:0006355, GO:0043586, GO:0060021, GO:0060485
LAIR1	GO:0005886, GO:0016021, GO:0070062, GO:0005515, GO:0002250, GO:0050776, GO:0002376
GREB1	GO:0016021, GO:0070062
GP6	GO:0005887, GO:0009986, GO:0070062, GO:0097197, GO:0004888, GO:0005518, GO:0007167, GO:0030168, GO:0050900
FAM13B	GO:0005829, GO:0005096, GO:0002230, GO:0007165, GO:0043547, GO:0051056, GO:0098779, GO:0098792
SLC2A5	GO:0005887, GO:0016324, GO:0042383, GO:0070062, GO:0005353, GO:0005355, GO:0070061, GO:0003044, GO:0005975, GO:0071332, GO:1904659, GO:1990539
ACACB	GO:0005634, GO:0005741, GO:0005829, GO:0012505, GO:0003989, GO:0004075, GO:0005515, GO:0005524, GO:0046872, GO:0006084, GO:0006633, GO:0006768, GO:0006853, GO:0010629, GO:0010884, GO:0010906, GO:0031325, GO:0031667, GO:0031999, GO:0043086, GO:0051289, GO:0060421, GO:0097009, GO:2001295
MS4A4A	GO:0016021
HTT	GO:0005654, GO:0005770, GO:0005776, GO:0005783, GO:0005794, GO:0005814, GO:0005829, GO:0014069, GO:0030136, GO:0030424, GO:0030425,

	GO:0030659, GO:0043025, GO:0043234, GO:0071598, GO:0002039, GO:0005102, GO:0005522, GO:0008134, GO:0034452, GO:0042802, GO:0044325, GO:0045505, GO:0048487, GO:0000132, GO:0006890, GO:0006915, GO:0007030, GO:0007417, GO:0031587, GO:0034047, GO:0042297, GO:0045724, GO:0047496, GO:0048513, GO:0051028, GO:2000117, GO:2001237
LAMA4	GO:0005605, GO:0070062, GO:0005102, GO:0005201, GO:0001568, GO:0007155, GO:0030155, GO:0030198, GO:0030334, GO:0045995, GO:0050873
TRPC4AP	GO:0005886, GO:0031464, GO:0005262, GO:0019902, GO:0006511, GO:0016567, GO:0070588
ZNF765	GO:0005634, GO:0003677, GO:0003700, GO:0005515, GO:0046872, GO:0006351, GO:0006355
ATP13A3	GO:0005887, GO:0043231, GO:0005524, GO:0019829, GO:0046872, GO:0006874, GO:0098655
GABRE	GO:0005887, GO:0030054, GO:0034707, GO:0045211, GO:1902711, GO:0004890, GO:0005230, GO:0005254, GO:0007214, GO:1902476
NUP210L	GO:0005643, GO:0016021, GO:0007286, GO:0060009
CMIP	GO:0005634, GO:0005737, GO:0005515
CBX7	GO:0000790, GO:0000792, GO:0005654, GO:0005737, GO:0035102, GO:0003682, GO:0003727, GO:0035064, GO:0000122, GO:0006351, GO:0042493, GO:0048733
CCDC88C	GO:0005737, GO:0030165, GO:0043621, GO:0001932, GO:0016055, GO:0031098, GO:0031648, GO:0051260
IL9R	GO:0005615, GO:0005887, GO:0004919, GO:0008283, GO:0038113, GO:1903955
TLL2	GO:0005576, GO:0004222, GO:0004252, GO:0005509, GO:0008270, GO:0006508, GO:0007275, GO:0022617, GO:0030154, GO:0048632
TRIM61	GO:0005622, GO:0008270
LGALS3	GO:0001772, GO:0005615, GO:0005681, GO:0005743, GO:0031012, GO:0070062, GO:0019863, GO:0030246, GO:0042056, GO:0043236, GO:0044822, GO:0002548, GO:0006397, GO:0008380, GO:0030593, GO:0030855, GO:0042129, GO:0045087, GO:0045806, GO:0048245, GO:0048246, GO:0050860, GO:0050918, GO:0070232, GO:0071677, GO:0090280, GO:1902041, GO:2000521, GO:2001189, GO:2001200, GO:2001237
ABTB1	GO:0005730, GO:0005737, GO:0005886, GO:0003746, GO:0005515, GO:0006414
LDLRAD1	GO:0016021, GO:0005515
NEDD4L	GO:0005654, GO:0005829, GO:0005886, GO:0070062, GO:0004842, GO:0016874, GO:0019870, GO:0019871, GO:0044325, GO:0000122, GO:0003254, GO:0006513, GO:0006814, GO:0006883, GO:0007588, GO:0010038, GO:0019058, GO:0030104, GO:0034220, GO:0042787, GO:0043161, GO:0045732, GO:0060306, GO:0070936, GO:0086005, GO:1901017, GO:1903861, GO:2000009, GO:2000650, GO:2001288
PEX1	GO:0005778, GO:0005829, GO:0070062, GO:0005524, GO:0008022, GO:0032403, GO:0042623, GO:0016558, GO:0060152
ARL5B	GO:0005622, GO:0005525, GO:0007264
CDC27	GO:0005654, GO:0005680, GO:0005813, GO:0005829, GO:0005876, GO:0019903, GO:0000090, GO:0007091, GO:0008283, GO:0031145, GO:0042787, GO:0051301, GO:0051436, GO:0051437, GO:0070979
ADARB1	GO:0005654, GO:0005730, GO:0005737, GO:0003725, GO:0003726, GO:0003729, GO:0005515, GO:0046872, GO:0006382, GO:0006397, GO:0008285, GO:0030336, GO:0044387, GO:0045070, GO:0045087, GO:0051607, GO:0051726
DPYSL3	GO:0005615, GO:0005829, GO:0030027, GO:0030426, GO:0031941, GO:0044297, GO:0070382, GO:0016810, GO:0017124, GO:0031005, GO:0035374, GO:0010976, GO:0010977, GO:0030336, GO:0048666, GO:0048678, GO:0051017, GO:0051260, GO:0051491, GO:0051764, GO:0071345
NSMAF	GO:0005829, GO:0012505, GO:0019898, GO:0005057, GO:0005515, GO:0005543, GO:0016230, GO:0006672, GO:0007165, GO:0043065, GO:0043085,

	GO:2000304
BAG1	GO:0005634, GO:0005829, GO:0000774, GO:0005057, GO:0051087, GO:0006915, GO:0007166, GO:0043066, GO:0050790, GO:0070389, GO:1900034
TMEM201	GO:0005637, GO:0016021, GO:0005521, GO:0051015, GO:0010761, GO:0030473
GHSR	GO:0005886, GO:0009986, GO:0016021, GO:0043005, GO:0045121, GO:0001616, GO:0016520, GO:0017046, GO:0007186, GO:0008154, GO:0008343, GO:0009755, GO:0030252, GO:0032094, GO:0032100, GO:0032691, GO:0032869, GO:0040018, GO:0042536, GO:0043134, GO:0043568, GO:0045409, GO:0045923, GO:0046676, GO:0046697, GO:0050728, GO:0051963
TANK	GO:0005829, GO:0031625, GO:0046872, GO:0007249, GO:0035666
FANCI	GO:0005654, GO:0005737, GO:0016020, GO:0003677, GO:0070182, GO:0007095, GO:0031398, GO:0036297
CMPK1	GO:0005634, GO:0005829, GO:0070062, GO:0004127, GO:0004550, GO:0004849, GO:0005524, GO:0009041, GO:0050145, GO:0006165, GO:0006207, GO:0006222, GO:0009142, GO:0015949, GO:0046940
PLCG1	GO:0001726, GO:0005829, GO:0005886, GO:0005911, GO:0008180, GO:0030027, GO:0004435, GO:0005057, GO:0005168, GO:0005509, GO:0030971, GO:0035254, GO:0000186, GO:0001701, GO:0007173, GO:0009395, GO:0016032, GO:0019722, GO:0038095, GO:0038096, GO:0043536, GO:0043647, GO:0045766, GO:0050852, GO:0050900, GO:0051281, GO:0071364
SNX13	GO:0016021, GO:0031901, GO:0032266, GO:0006886, GO:0009968, GO:0043547
TBC1D10B	GO:0005829, GO:0005886, GO:0012505, GO:0005096, GO:0017137, GO:0006886, GO:0031338, GO:0042147, GO:0090630, GO:1902017
XDH	GO:0005615, GO:0005777, GO:0005829, GO:0016529, GO:0004854, GO:0004855, GO:0005506, GO:0009055, GO:0016614, GO:0016903, GO:0030151, GO:0042803, GO:0043546, GO:0050660, GO:0051537, GO:0001933, GO:0001937, GO:0006195, GO:0006919, GO:0007595, GO:0009115, GO:0010629, GO:0045602, GO:0051898, GO:0055114, GO:1900745, GO:1900747, GO:2000379, GO:2001213
CXCR2	GO:0005887, GO:0009986, GO:0042629, GO:0004918, GO:0019959, GO:0002407, GO:0006954, GO:0006968, GO:0007200, GO:0008284, GO:0030593, GO:0031623, GO:0038112, GO:0042119, GO:0070098
MAPKAP1	GO:0005654, GO:0005794, GO:0005829, GO:0005886, GO:0016023, GO:0031932, GO:0005546, GO:0005547, GO:0016301, GO:0017016, GO:0019901, GO:0043325, GO:0070300, GO:0080025, GO:0016310, GO:0021762, GO:0031295, GO:0046580, GO:0048015
TMEM91	GO:0016021, GO:0002244, GO:0009607
WDR13	GO:0005654, GO:0005737
TIAF1	GO:0005634, GO:0006915, GO:0007249, GO:0043066
WDR3	GO:0005654, GO:0031965, GO:0032040, GO:0034388, GO:0030515, GO:0044822, GO:0000462
DNASE1	GO:0005635, GO:0070062, GO:0003779, GO:0004530, GO:0006308, GO:0006915, GO:0090305
PHACTR2	GO:0005886, GO:0031092, GO:0003779, GO:0004864, GO:0002576, GO:0043086
C22orf29	GO:0005739, GO:0005515, GO:0051881, GO:0097345
PFKFB2	GO:0005829, GO:0003873, GO:0004331, GO:0005524, GO:0019901, GO:0006000, GO:0006003, GO:0006089, GO:0009749, GO:0016311, GO:0032024, GO:0033133, GO:0046835, GO:0061621
FAM19A2	GO:0005737
LRRN4	GO:0005887, GO:0070062, GO:0007616, GO:0008542

CEP250	GO:0005814, GO:0005829, GO:0005929, GO:0043234, GO:0048471, GO:0070062, GO:0008022, GO:0019901, GO:0019904, GO:0000086, GO:0010457, GO:0030997, GO:0033365, GO:0035058
GABARAP	GO:0000139, GO:0000421, GO:0005764, GO:0005790, GO:0005829, GO:0005874, GO:0005875, GO:0005886, GO:0005930, GO:0015629, GO:0031410, GO:0044297, GO:0048471, GO:0097225, GO:0008017, GO:0016791, GO:0031625, GO:0048487, GO:0050811, GO:0000045, GO:0000226, GO:0000422, GO:0006605, GO:0006995, GO:0007268, GO:0008625, GO:0016311
FRY	GO:0000922, GO:0005815, GO:0005938, GO:0030427, GO:0004857, GO:0000902, GO:0031175, GO:0043086, GO:0090527, GO:1904428
NFATC3	GO:0005654, GO:0005730, GO:0005829, GO:0000978, GO:0001077, GO:0001227, GO:0005515, GO:0006366, GO:0006954, GO:0038095, GO:0045944, GO:1902894
PARK2	GO:0005634, GO:0005739, GO:0005783, GO:0005794, GO:0005829, GO:0016235, GO:0019005, GO:0043005, GO:0048471, GO:0071797, GO:0097413, GO:0098793, GO:1990452, GO:0000976, GO:0001664, GO:0003700, GO:0003779, GO:0008013, GO:0008270, GO:0015631, GO:0016874, GO:0017124, GO:0019901, GO:0030165, GO:0030544, GO:0031624, GO:0031625, GO:0042802, GO:0042826, GO:0043130, GO:0043274, GO:0051087, GO:0097602, GO:1904264, GO:1990381, GO:1990444, GO:0000122, GO:0000266, GO:0001964, GO:0006351, GO:0006513, GO:0006979, GO:0007165, GO:0007417, GO:0007612, GO:0008344, GO:0010636, GO:0010637, GO:0010994, GO:0014059, GO:0031648, GO:0032232, GO:0032368, GO:0032436, GO:0033132, GO:0034620, GO:0035249, GO:0035519, GO:0036503, GO:0042053, GO:0042415, GO:0042417, GO:0042787, GO:0043123, GO:0043161, GO:0043388, GO:0044314, GO:0044828, GO:0045944, GO:0046329, GO:0046676, GO:0050821, GO:0051582, GO:0051583, GO:0051865, GO:0051881, GO:0055069, GO:0070050, GO:0070534, GO:0070585, GO:0070842, GO:0070936, GO:0070979, GO:0071287, GO:0085020, GO:0090090, GO:0090141, GO:0090201, GO:0097237, GO:0098779, GO:1902254, GO:1902283, GO:1902530, GO:1902803, GO:1903146, GO:1903214, GO:1903265, GO:1903351, GO:1903377, GO:1903378, GO:1903382, GO:1903861, GO:1904049, GO:2000378
SCN4B	GO:0001518, GO:0014704, GO:0017080, GO:0044325, GO:0086006, GO:0010765, GO:0035725, GO:0060078, GO:0060307, GO:0086002, GO:0086012, GO:0086016, GO:0086091, GO:2000649
SEC61B	GO:0005784, GO:0005829, GO:0016021, GO:0031205, GO:0015450, GO:0043022, GO:0044822, GO:0048408, GO:0000060, GO:0030433, GO:0030970, GO:0036498, GO:0071806
VPS18	GO:0005765, GO:0005769, GO:0005776, GO:0005884, GO:0030123, GO:0030136, GO:0030897, GO:0031902, GO:0033263, GO:0003779, GO:0019905, GO:0030674, GO:0046872, GO:0006886, GO:0006904, GO:0006914, GO:0007032, GO:0007040, GO:0008333, GO:0035542, GO:0046718
KNDC1	GO:0030425, GO:0032045, GO:0043025, GO:0005088, GO:0001934, GO:0007264, GO:0021707, GO:0043547, GO:0048814
CHIC1	GO:0005886, GO:0016021, GO:0016023, GO:0006810
MEFV	GO:0001726, GO:0005634, GO:0005776, GO:0005829, GO:0005874, GO:0005875, GO:0030027, GO:0031410, GO:0003779, GO:0008270, GO:0006954, GO:0010508, GO:0032691, GO:0032695, GO:0034341, GO:0071641, GO:1900016, GO:1900226, GO:2001056
MED1	GO:0000151, GO:0000785, GO:0005730, GO:0016020, GO:0016592, GO:0032993, GO:0000978, GO:0000981, GO:0001047, GO:0001104, GO:0004872, GO:0016922, GO:0030331, GO:0030375, GO:0031490, GO:0036033, GO:0042809, GO:0042974, GO:0042975, GO:0046966, GO:0050693, GO:0061630, GO:0000122, GO:0000902, GO:0001525, GO:0001889, GO:0001892, GO:0002088, GO:0002154, GO:0003222, GO:0003406, GO:0006356, GO:0006367, GO:0006590, GO:0006702, GO:0007420, GO:0007595, GO:0010839, GO:0016567, GO:0030216, GO:0030224, GO:0030521, GO:0031100, GO:0033148, GO:0033160, GO:0033601, GO:0035050, GO:0035116, GO:0035162, GO:0035357, GO:0035729, GO:0035855, GO:0042789, GO:0043066, GO:0044255, GO:0045444, GO:0045618, GO:0045648, GO:0045665, GO:0045944, GO:0048822, GO:0060335, GO:0060744, GO:0060745, GO:0060750, GO:0070318, GO:0070371, GO:0070562, GO:0071364, GO:0097067, GO:2000273, GO:2000347

BCL9L	GO:0005654, GO:0005730, GO:0003713, GO:0008013, GO:0006351, GO:0010718, GO:0022604, GO:0030512, GO:0035019, GO:0035914, GO:0045944, GO:0060070, GO:1904837
OR51L1	GO:0005886, GO:0016021, GO:0031012, GO:0004222, GO:0004930, GO:0004984, GO:0008270, GO:0006508, GO:0007186, GO:0050911
SLC12A5	GO:0005887, GO:0043025, GO:0043198, GO:0015379, GO:0019901, GO:0022820, GO:0007268, GO:0007612, GO:0030644, GO:0035264, GO:0040040, GO:0042493, GO:0060996, GO:0071805, GO:1902476
NQO2	GO:0005654, GO:0005737, GO:0070062, GO:0005515, GO:0008753, GO:0009055, GO:0046872, GO:0007613, GO:0055114
NDUFA9	GO:0005654, GO:0005747, GO:0005759, GO:0008137, GO:0032403, GO:0050662, GO:0006120, GO:0006814, GO:0032981, GO:1901006
ACIN1	GO:0005730, GO:0005829, GO:0005886, GO:0016607, GO:0035145, GO:0061574, GO:0000166, GO:0016887, GO:0019899, GO:0044822, GO:0002230, GO:0006397, GO:0008380, GO:0030218, GO:0030263, GO:0043065, GO:0045657, GO:0048025, GO:0098779, GO:0098792
PPP2R2B	GO:0000159, GO:0008601, GO:0034047
GCSAM	GO:0005737, GO:0005886, GO:0003779, GO:0019901, GO:0045159, GO:0050855, GO:2000402
ERN1	GO:0005637, GO:0005739, GO:0030176, GO:1990332, GO:1990597, GO:1990604, GO:1990630, GO:0000287, GO:0004521, GO:0004674, GO:0005524, GO:0019899, GO:0030544, GO:0042803, GO:0043531, GO:0051879, GO:0001935, GO:0006351, GO:0006355, GO:0006402, GO:0006987, GO:0007050, GO:0007257, GO:0016241, GO:0033120, GO:0035924, GO:0070055, GO:0070059, GO:0071333, GO:0090502, GO:1900103, GO:1901142, GO:1990579
MAFG	GO:0005654, GO:0001228, GO:0043565, GO:0046982, GO:0001701, GO:0006366, GO:0007596, GO:0030534, GO:0030641, GO:0042127, GO:0045604, GO:0045944
NTN1	GO:0005604, GO:0005737, GO:0071944, GO:0005515, GO:0001764, GO:0006915, GO:0006930, GO:0008284, GO:0016337, GO:0030334, GO:0030517, GO:0032488, GO:0033564, GO:0040023, GO:0042472, GO:0045773, GO:0060603, GO:2000147
ALG2	GO:0005634, GO:0005789, GO:0016021, GO:0048471, GO:0000033, GO:0004378, GO:0046982, GO:0047485, GO:0048306, GO:0006488, GO:0033577, GO:0051592, GO:0097502
TECPR1	GO:0000421, GO:0005765, GO:0016021, GO:0031410, GO:0005515, GO:0032266, GO:0097352
EXOG	GO:0005743, GO:0043234, GO:0003676, GO:0004519, GO:0004527, GO:0046872, GO:0090305
CENPC	GO:0000777, GO:0000780, GO:0005654, GO:0005721, GO:0005829, GO:0019237, GO:0000090, GO:0000236, GO:0007062, GO:0007067, GO:0034080, GO:0051301, GO:0051382
NADSYN1	GO:0005829, GO:0003952, GO:0004359, GO:0005515, GO:0005524, GO:0009435
MCF2L	GO:0005615, GO:0005829, GO:0012505, GO:0031234, GO:0005089, GO:0035091, GO:0035025, GO:0035556, GO:0043065, GO:0043547
B3GALT5	GO:0000139, GO:0005783, GO:0016021, GO:0008499, GO:0006486
PPARD	GO:0000790, GO:0005654, GO:0001227, GO:0003707, GO:0003713, GO:0004879, GO:0008144, GO:0008270, GO:0043565, GO:0046982, GO:0051059, GO:0070539, GO:0006006, GO:0006029, GO:0006091, GO:0006367, GO:0006635, GO:0006776, GO:0007507, GO:0007566, GO:0008203, GO:0008366, GO:0008654, GO:0009299, GO:0009749, GO:0014068, GO:0014823, GO:0014842, GO:0014912, GO:0015758, GO:0015908, GO:0030154, GO:0030308, GO:0030522, GO:0031589, GO:0032024, GO:0032966, GO:0033189, GO:0042060, GO:0043066, GO:0043401, GO:0043415, GO:0043616, GO:0045600, GO:0045662, GO:0045684, GO:0045893, GO:0045909, GO:0046697, GO:0048662, GO:0050680, GO:0050728, GO:0051546, GO:0060612, GO:0071222, GO:0071456, GO:0097190, GO:1902894, GO:2000288

CLEC16A	GO:0005765, GO:0010008, GO:0030246, GO:0006914
UHRF1BP1	GO:0042802, GO:0042826
CNDP2	GO:0005654, GO:0005829, GO:0070062, GO:0004180, GO:0008237, GO:0034701, GO:0046872, GO:0102008, GO:0000096, GO:0006508, GO:0006750
CHEK2	GO:0000781, GO:0005794, GO:0016605, GO:0004674, GO:0005524, GO:0019901, GO:0031625, GO:0042803, GO:0046872, GO:0000086, GO:0001302, GO:0001934, GO:0006302, GO:0006351, GO:0006975, GO:0006977, GO:0008630, GO:0010332, GO:0018105, GO:0035690, GO:0042176, GO:0044257, GO:0045893, GO:0046777, GO:0050821, GO:0051301, GO:0071157, GO:0072428, GO:0090307, GO:0090399, GO:1901796, GO:1902520, GO:1903926, GO:2000002, GO:2000210
MYOF	GO:0005901, GO:0016021, GO:0030659, GO:0031965, GO:0070062, GO:0005515, GO:0005543, GO:0001778, GO:0006936, GO:0008015, GO:0030947, GO:0034605
FAM134C	GO:0016021, GO:0005515, GO:0010976
FBXO36	GO:0031146
CAPN15	GO:0005737, GO:0003700, GO:0004198, GO:0008270, GO:0006355, GO:0006508
VRK3	GO:0005634, GO:0005737, GO:0004674, GO:0005524, GO:0019903, GO:0008360, GO:0018105, GO:0032516, GO:0070373
MECP2	GO:0000790, GO:0000792, GO:0005615, GO:0005739, GO:0005829, GO:0043234, GO:0098794, GO:0000400, GO:0003700, GO:0003714, GO:0003729, GO:0008134, GO:0008327, GO:0010385, GO:0019904, GO:0031490, GO:0035197, GO:0042826, GO:0045322, GO:0047485, GO:0000122, GO:0001662, GO:0001666, GO:0001964, GO:0001976, GO:0002087, GO:0006020, GO:0006122, GO:0006342, GO:0006349, GO:0006351, GO:0006541, GO:0006576, GO:0007416, GO:0007507, GO:0007585, GO:0007616, GO:0008104, GO:0008211, GO:0008284, GO:0008344, GO:0008542, GO:0009405, GO:0009791, GO:0010212, GO:0010288, GO:0016358, GO:0016571, GO:0016573, GO:0019230, GO:0019233, GO:0021549, GO:0021591, GO:0031061, GO:0032048, GO:0032355, GO:0035067, GO:0035176, GO:0042551, GO:0043524, GO:0044030, GO:0045893, GO:0046470, GO:0048712, GO:0050432, GO:0051965, GO:0060079, GO:0060291
SLITRK3	GO:0016021, GO:0007409, GO:0051965
DAPK3	GO:0005737, GO:0005884, GO:0016605, GO:0045121, GO:0004674, GO:0005524, GO:0008022, GO:0008140, GO:0017048, GO:0042803, GO:0043522, GO:0000910, GO:0006351, GO:0006355, GO:0006940, GO:0007088, GO:0008360, GO:0010506, GO:0017148, GO:0030182, GO:0030335, GO:0035556, GO:0043519, GO:0046777, GO:0051893, GO:0071346, GO:0090263, GO:0097190, GO:2000249, GO:2001241
DDX11	GO:0000790, GO:0000922, GO:0005654, GO:0005730, GO:0030496, GO:0070062, GO:0003690, GO:0003697, GO:0003723, GO:0004003, GO:0005515, GO:0005524, GO:0046872, GO:0051539, GO:0006139, GO:0007062, GO:0016032, GO:0032508, GO:0036498
ZNF264	GO:0005634, GO:0000978, GO:0005515, GO:0046872, GO:0006351, GO:0006355
SIX2	GO:0005654, GO:0005737, GO:0005886, GO:0031965, GO:0000978, GO:0001077, GO:0008134, GO:0032403, GO:0002062, GO:0003337, GO:0006366, GO:0006606, GO:0007501, GO:0009948, GO:0016477, GO:0030278, GO:0032330, GO:0042474, GO:0045596, GO:0045944, GO:0048557, GO:0048701, GO:0072038, GO:0072161, GO:0090189, GO:0097168, GO:1902732
MBD4	GO:0000785, GO:0005654, GO:0003696, GO:0004520, GO:0005515, GO:0008263, GO:0009314, GO:0032355, GO:0045008
ANKIB1	GO:0000151, GO:0005737, GO:0008270, GO:0031624, GO:0061630, GO:0000209, GO:0032436, GO:0042787
CCDC103	GO:0005737, GO:0005930, GO:0031514, GO:0042803, GO:0001947, GO:0036158, GO:0036159, GO:0060287, GO:0071907

PRUNE1	GO:0005634, GO:0005737, GO:0005925, GO:0004427, GO:0005515, GO:0046872
NOTCH2	GO:0000139, GO:0005576, GO:0005654, GO:0005789, GO:0005887, GO:0009986, GO:0043235, GO:0005509, GO:0005515, GO:0038049, GO:0001709, GO:0002315, GO:0003184, GO:0006355, GO:0006367, GO:0006915, GO:0007050, GO:0007399, GO:0008285, GO:0016049, GO:0019827, GO:0030522, GO:0042246, GO:0043066, GO:0046579, GO:0046849, GO:0050793, GO:0060413, GO:0061314
KIAA1217	GO:0005737, GO:0005515, GO:0048706
UTP15	GO:0001650, GO:0005654, GO:0005737, GO:0019013, GO:0030054, GO:0030529, GO:0005515, GO:0044822, GO:0006364
NPHS2	GO:0005783, GO:0005887, GO:0031235, GO:0036057, GO:0043234, GO:0045121, GO:0070062, GO:0005515, GO:0007588, GO:0031532, GO:0072249
HR	GO:0000118, GO:0016604, GO:0003677, GO:0003700, GO:0003714, GO:0016491, GO:0042809, GO:0042826, GO:0046872, GO:0046966, GO:0006351, GO:0043433, GO:0045892, GO:0051291, GO:0055114
CNTNAP1	GO:0008076, GO:0033270, GO:0043209, GO:0004872, GO:0005070, GO:0017124, GO:0002175, GO:0007010, GO:0007155, GO:0007165, GO:0009967, GO:0019227, GO:0030913, GO:0048812, GO:0050884, GO:0050885
FGFR1OP	GO:0005634, GO:0005813, GO:0005829, GO:0048471, GO:0004713, GO:0019901, GO:0030292, GO:0042803, GO:0000086, GO:0008284, GO:0018108, GO:0030307, GO:0030335, GO:0034453, GO:0061099
HSPB8	GO:0005654, GO:0005794, GO:0004672, GO:0042802, GO:1900034
KIF21B	GO:0005737, GO:0005871, GO:0005874, GO:0003777, GO:0005524, GO:0008017, GO:0016887, GO:0007018
MED17	GO:0000151, GO:0005667, GO:0016020, GO:0016592, GO:0001104, GO:0004872, GO:0030374, GO:0042809, GO:0046966, GO:0061630, GO:0006367, GO:0016567, GO:0019827, GO:0030521, GO:0045944
KCNA1	GO:0005783, GO:0005829, GO:0008076, GO:0009986, GO:0016023, GO:0016324, GO:0030054, GO:0030425, GO:0033270, GO:0042734, GO:0043204, GO:0043679, GO:0044224, GO:0005251, GO:0005515, GO:0001964, GO:0006937, GO:0007268, GO:0007405, GO:0010644, GO:0010960, GO:0019228, GO:0021766, GO:0023041, GO:0034613, GO:0034765, GO:0050966, GO:0050976, GO:0051260, GO:0071286, GO:0071805
PLCH2	GO:0005737, GO:0005886, GO:0004435, GO:0004871, GO:0005509, GO:0016042, GO:0035556, GO:0043647, GO:0046488
MFSD6	GO:0016021
TRIM7	GO:0005634, GO:0005737, GO:0005515, GO:0008270
FOXRED2	GO:0005788, GO:0001948, GO:0004497, GO:0005515, GO:0050660, GO:0030433, GO:0055114
LRCH4	GO:0016021, GO:0016605, GO:0005515, GO:0007399
PQLC1	GO:0016021
POLR3H	GO:0005666, GO:0005813, GO:0005829, GO:0001056, GO:0003677, GO:0006384, GO:0032481, GO:0045087, GO:0051607
MICALCL	GO:0005634, GO:0005737, GO:0003779, GO:0008270, GO:0016709, GO:0043914, GO:0051019, GO:0071949, GO:0007275, GO:0007283, GO:0010735, GO:0019417, GO:0030042, GO:0030154
SREBF1	GO:0000139, GO:0005635, GO:0005654, GO:0005789, GO:0005829, GO:0012507, GO:0016021, GO:0043234, GO:0000978, GO:0001077, GO:0003682, GO:0019901, GO:0032403, GO:0032810, GO:0046983, GO:0000122, GO:0003062, GO:0007568, GO:0007623, GO:0008203, GO:0008286, GO:0008610, GO:0009267, GO:0009749, GO:0010867, GO:0019217, GO:0030324, GO:0031065, GO:0031647, GO:0032094, GO:0032526, GO:0032570, GO:0033762, GO:0042493, GO:0042789, GO:0045444, GO:0045542, GO:0045944, GO:0046676, GO:0051591, GO:0071398, GO:1903146, GO:1903214

ABCC5	GO:0005887, GO:0005524, GO:0008514, GO:0043225, GO:0015711, GO:0030213, GO:0098656
ZC3HAV1	GO:0005634, GO:0005764, GO:0005770, GO:0005794, GO:0003950, GO:0005515, GO:0044822, GO:0046872, GO:0032727, GO:0032728, GO:0039507, GO:0043123, GO:0045071, GO:0045087, GO:0050691, GO:0051607, GO:0061014, GO:0071360, GO:1900246
PDE1C	GO:0005829, GO:0005929, GO:0004117, GO:0005516, GO:0046872, GO:0007165, GO:0007608
TIAM1	GO:0005634, GO:0005829, GO:0005874, GO:0031234, GO:0032587, GO:0043025, GO:0043197, GO:0044291, GO:0044295, GO:0044304, GO:0005057, GO:0008017, GO:0008289, GO:0030676, GO:0030971, GO:0048365, GO:0003300, GO:0006915, GO:0007160, GO:0016477, GO:0016601, GO:0032092, GO:0035023, GO:0042220, GO:0043065, GO:0043507, GO:0048013, GO:0050772, GO:0060071, GO:0061003, GO:0061178, GO:0070372, GO:0072657, GO:0090630, GO:1904268, GO:1904338, GO:1990138, GO:2000050
CDX1	GO:0005634, GO:0000980, GO:0001205, GO:0005515, GO:0006366, GO:0009948, GO:0014807, GO:0030154, GO:0045944, GO:0060349
GLCCI1	GO:0016021
TPRG1	GO:0005737
CTNNA1	GO:0001669, GO:0005794, GO:0005829, GO:0005915, GO:0005925, GO:0014704, GO:0015629, GO:0016342, GO:0016600, GO:0030027, GO:0005198, GO:0008013, GO:0017166, GO:0044822, GO:0045295, GO:0045296, GO:0051015, GO:0001541, GO:0007015, GO:0007163, GO:0007406, GO:0007568, GO:0008584, GO:0016264, GO:0031103, GO:0034332, GO:0034613, GO:0042475, GO:0043297, GO:0043627, GO:0045880, GO:0051149, GO:0051291, GO:0071681, GO:0090136, GO:2000146, GO:2001045, GO:2001240, GO:2001241
CDC42BPA	GO:0005737, GO:0005911, GO:0031252, GO:0042641, GO:0070062, GO:0000287, GO:0004674, GO:0005524, GO:0042802, GO:0006468, GO:0016477, GO:0031032, GO:0031532, GO:0035556
DLG4	GO:0005783, GO:0008021, GO:0008076, GO:0014069, GO:0016323, GO:0030054, GO:0030666, GO:0030863, GO:0031234, GO:0032281, GO:0032839, GO:0043197, GO:0044224, GO:0044300, GO:0044306, GO:0045211, GO:0004385, GO:0005088, GO:0005198, GO:0008022, GO:0015276, GO:0019901, GO:0019903, GO:0030165, GO:0031697, GO:0031748, GO:0031812, GO:0032403, GO:0033130, GO:0035255, GO:0042043, GO:0097109, GO:0097110, GO:0000165, GO:0002091, GO:0006461, GO:0007204, GO:0007268, GO:0007612, GO:0016188, GO:0034220, GO:0035176, GO:0035641, GO:0042220, GO:0043547, GO:0045161, GO:0045184, GO:0045197, GO:0046037, GO:0046710, GO:0048169, GO:0050885, GO:0060997, GO:0071625, GO:0097113, GO:0097120, GO:2000310, GO:2000463, GO:2000821
PLEKHG4B	GO:0005089, GO:0035023, GO:0043547
ZDHHC2	GO:0005783, GO:0005794, GO:0005887, GO:0055038, GO:0008270, GO:0019706, GO:0018345
MARCH7	GO:0008270, GO:0016874, GO:0002643, GO:0016567, GO:0042130
DOCK6	GO:0005829, GO:0048471, GO:0005085, GO:0007264, GO:0007596, GO:0043547
ZEB1	GO:0005654, GO:0005667, GO:0005737, GO:0001227, GO:0003682, GO:0003713, GO:0003714, GO:0008134, GO:0008270, GO:0070888, GO:0000122, GO:0006351, GO:0006955, GO:0007389, GO:0007417, GO:0008283, GO:0008285, GO:0010464, GO:0017015, GO:0030857, GO:0033081, GO:0045666, GO:0045944, GO:0048596, GO:0048704, GO:0048752, GO:0051150, GO:0051216, GO:0071230, GO:0090103
WDR1	GO:0002102, GO:0005829, GO:0005884, GO:0030054, GO:0042995, GO:0043209, GO:0070062, GO:0003779, GO:0002576, GO:0007605, GO:0030836, GO:0045214, GO:0048713, GO:0060307
RRP8	GO:0005677, GO:0005730, GO:0005737, GO:0005886, GO:0033553, GO:0008757, GO:0035064, GO:0044822, GO:0000183, GO:0006351, GO:0006364, GO:0032259, GO:0042149, GO:0046015, GO:0071158, GO:0072332

PPP4R4	GO:0005737, GO:0008287, GO:0005515, GO:0019888, GO:0032515, GO:0080163
ARHGAP4	GO:0005794, GO:0005829, GO:0005874, GO:0030426, GO:0005070, GO:0005089, GO:0005096, GO:0048365, GO:0007010, GO:0007266, GO:0009967, GO:0010764, GO:0030517, GO:0043065, GO:0043547, GO:0051056
PDE3A	GO:0005829, GO:0016021, GO:0004115, GO:0004119, GO:0030552, GO:0046872, GO:0047555, GO:0001556, GO:0006198, GO:0016101, GO:0019933, GO:0019934, GO:0040020, GO:0042493, GO:0043066, GO:0043116, GO:0043117, GO:0051591, GO:0060282, GO:0071321, GO:0071560
PDPR	GO:0005759, GO:0004741, GO:0016491, GO:0010510, GO:0016311, GO:0055114
FNDC5	GO:0005576, GO:0005778, GO:0005783, GO:0005886, GO:0016021, GO:0005179, GO:0014850, GO:0090336
BTN2A2	GO:0009986, GO:0016021, GO:0070062, GO:0005515, GO:0014067, GO:0045591, GO:0046007, GO:0050710, GO:0050860, GO:0051898, GO:0070373, GO:2000134
LRRC7	GO:0005737, GO:0014069, GO:0015629, GO:0030054, GO:0030175, GO:0043194, GO:0043197, GO:0045211, GO:0008022, GO:0010976, GO:0014070
FAM13A	GO:0005829, GO:0005096, GO:0007165, GO:0043547, GO:0051056
GBGT1	GO:0000139, GO:0016021, GO:0046872, GO:0047277, GO:0005975, GO:0006486, GO:0009247
NUP62	GO:0000922, GO:0005642, GO:0005737, GO:0030529, GO:0031074, GO:0031965, GO:0044613, GO:0046930, GO:0090543, GO:0003682, GO:0005487, GO:0005543, GO:0017056, GO:0019894, GO:0030159, GO:0030544, GO:0042169, GO:0043130, GO:0046966, GO:0051425, GO:0051879, GO:0006351, GO:0006406, GO:0006409, GO:0006606, GO:0007067, GO:0007077, GO:0007166, GO:0007283, GO:0007569, GO:0008219, GO:0008285, GO:0009755, GO:0010827, GO:0016925, GO:0019083, GO:0031047, GO:0042059, GO:0042306, GO:0043066, GO:0043123, GO:0043407, GO:0045742, GO:0045893, GO:0046580, GO:0070208, GO:0075733, GO:1900034
INTU	GO:0005737, GO:0009986, GO:0010839, GO:0021513, GO:0021915, GO:0030216, GO:0030278, GO:0031069, GO:0042733, GO:0045880, GO:0051782, GO:1903887
HEATR5B	GO:0016020, GO:0070062
MCC	GO:0005654, GO:0005737, GO:0005886, GO:0030027, GO:0004872, GO:0005509, GO:0005515, GO:0010633, GO:0016055, GO:0045184, GO:0050680, GO:0090090
LLGL1	GO:0000137, GO:0005886, GO:0030424, GO:0030864, GO:0031901, GO:0032588, GO:0005096, GO:0005198, GO:0017137, GO:0019901, GO:0019905, GO:0006461, GO:0006887, GO:0006893, GO:0007409, GO:0008593, GO:0017157, GO:0030866, GO:0032878, GO:0043547, GO:0050708, GO:0051294
WWP2	GO:0000151, GO:0005634, GO:0005737, GO:0016020, GO:0070062, GO:0001085, GO:0001190, GO:0016874, GO:0061630, GO:0000122, GO:0006366, GO:0032410, GO:0042391, GO:0042787, GO:0043161, GO:0043433, GO:0045944, GO:0046718, GO:0051224, GO:0051865, GO:0070534, GO:1901016
LMNB1	GO:0005637, GO:0005638, GO:0005654, GO:0016363, GO:0005198, GO:0043274
TGM1	GO:0001533, GO:0031224, GO:0070062, GO:0003810, GO:0005515, GO:0046872, GO:0010838, GO:0018149, GO:0031424, GO:0043163, GO:0045787
NDUFAF3	GO:0005634, GO:0005743, GO:0005515, GO:0032981
RAB21	GO:0005789, GO:0005802, GO:0005925, GO:0009898, GO:0030659, GO:0032154, GO:0032580, GO:0070062, GO:0098559, GO:1904115, GO:0003924, GO:0005515, GO:0005525, GO:0019003, GO:0007264, GO:0008089, GO:0015031, GO:0017157, GO:0030516, GO:0048260, GO:0050775, GO:2000643
CENPO	GO:0000777, GO:0005654, GO:0005829, GO:0005515, GO:0000090, GO:0000236, GO:0007062, GO:0034080
LYST	GO:0005737, GO:0015630, GO:0005515, GO:0007040, GO:0015031, GO:0030595, GO:0032438, GO:0032510, GO:0033364, GO:0042267, GO:0042742,

	GO:0042832, GO:0051607
KCNQ4	GO:0005737, GO:0008076, GO:0009925, GO:0043005, GO:0005251, GO:0005515, GO:0007605, GO:0032227, GO:0034765, GO:0042472, GO:0071805
MTPN	GO:0005634, GO:0005829, GO:0008290, GO:0030424, GO:0048471, GO:0070062, GO:0005515, GO:0043565, GO:0006417, GO:0006584, GO:0010557, GO:0010613, GO:0016049, GO:0016202, GO:0021707, GO:0030307, GO:0043403, GO:0051092, GO:0051146, GO:0051247, GO:0071260, GO:2000812
CSPG4	GO:0005796, GO:0005887, GO:0005925, GO:0009986, GO:0016324, GO:0031258, GO:0043202, GO:0070062, GO:0004871, GO:0019901, GO:0000187, GO:0001525, GO:0007169, GO:0008283, GO:0008347, GO:0030206, GO:0030207, GO:0030208, GO:0035556, GO:0048771, GO:0050731
SLC8A1	GO:0005739, GO:0005829, GO:0005874, GO:0005887, GO:0014704, GO:0030018, GO:0030315, GO:0043197, GO:0043198, GO:0005432, GO:0005509, GO:0005516, GO:0030506, GO:0044325, GO:0002027, GO:0002028, GO:0006883, GO:0007584, GO:0009749, GO:0010649, GO:0010763, GO:0010881, GO:0014829, GO:0021537, GO:0030501, GO:0033198, GO:0034614, GO:0035902, GO:0035994, GO:0042493, GO:0042542, GO:0044557, GO:0051481, GO:0055013, GO:0055119, GO:0060048, GO:0060402, GO:0070509, GO:0070588, GO:0071313, GO:0071320, GO:0071436, GO:0071456, GO:0086012, GO:0086064, GO:0097369, GO:0098735, GO:1901660, GO:1903779
HSPA12B	GO:0005515, GO:0005524
TTBK2	GO:0005615, GO:0005634, GO:0005814, GO:0005829, GO:0035869, GO:0036064, GO:0045095, GO:0004674, GO:0005198, GO:0005515, GO:0005524, GO:0007224, GO:0008360, GO:0018105, GO:0042384
PRDM15	GO:0005634, GO:0000977, GO:0003700, GO:0008168, GO:0046872, GO:0006351, GO:0006355, GO:0032259
SLC45A4	GO:0016021, GO:0008506, GO:0015770, GO:0055085
LRWD1	GO:0005664, GO:0005721, GO:0005737, GO:0005815, GO:0031933, GO:0003682, GO:0008327, GO:0035064, GO:0006270, GO:0006325, GO:0071169
CLCNKB	GO:0005887, GO:0034707, GO:0005247, GO:0046872, GO:0007588, GO:1902476, GO:1903959
RTKN2	GO:0007165, GO:0030097
EIF3H	GO:0005829, GO:0016020, GO:0016282, GO:0033290, GO:0070062, GO:0071541, GO:0003743, GO:0005515, GO:0044822, GO:0001731, GO:0006446
SOX12	GO:0005654, GO:0032993, GO:0000976, GO:0001077, GO:0001105, GO:0006366, GO:0021510, GO:0045165, GO:0045944, GO:0065004
SH3PXD2B	GO:0002102, GO:0005737, GO:0030054, GO:0042995, GO:0010314, GO:0032266, GO:0042169, GO:0070273, GO:0080025, GO:0001654, GO:0006801, GO:0007507, GO:0022617, GO:0030154, GO:0045600, GO:0060348, GO:0060612, GO:0071800, GO:0072657
ZHX2	GO:0005654, GO:0005730, GO:0005737, GO:0005886, GO:0003677, GO:0003700, GO:0003714, GO:0042803, GO:0046872, GO:0046982, GO:0000122, GO:0006351, GO:0006402, GO:0035019, GO:0045665
UBE2G2	GO:0005783, GO:0005829, GO:0005524, GO:0031625, GO:0042802, GO:0061630, GO:0061631, GO:0018279, GO:0030433, GO:0035458, GO:0070936, GO:1904153
TGIF1	GO:0005654, GO:0000978, GO:0001078, GO:0003714, GO:0070410, GO:0000122, GO:0006351, GO:0007275, GO:0042493, GO:0071363
TRIM40	GO:0008385, GO:0005515, GO:0008270, GO:0030308, GO:0032088, GO:0042177, GO:0045116, GO:1900181, GO:1901223
TG	GO:0005615, GO:0005783, GO:0005794, GO:0043234, GO:0048471, GO:0005179, GO:0032403, GO:0042803, GO:0043168, GO:0051087, GO:0052689, GO:0006590, GO:0007165, GO:0009268, GO:0015705, GO:0030878, GO:0031641, GO:0032496, GO:0042446, GO:0045056
CTDP1	GO:0000922, GO:0005737, GO:0005813, GO:0015629, GO:0016591, GO:0030496, GO:0051233, GO:0005515, GO:0008420, GO:0006368, GO:0010458, GO:0050434, GO:0051301, GO:0061052, GO:0070940

RPS6KA6	GO:0005654, GO:0005730, GO:0005739, GO:0005829, GO:0000287, GO:0004674, GO:0005524, GO:0006468, GO:0006978, GO:0007417, GO:0045992, GO:0070373, GO:2000381
ALS2	GO:0001726, GO:0005769, GO:0005813, GO:0005829, GO:0014069, GO:0016020, GO:0030027, GO:0030424, GO:0030426, GO:0031982, GO:0043025, GO:0043197, GO:0043234, GO:0005087, GO:0017112, GO:0017137, GO:0030676, GO:0042803, GO:0043539, GO:0001662, GO:0001701, GO:0001881, GO:0006979, GO:0007032, GO:0007409, GO:0007528, GO:0007626, GO:0008104, GO:0008219, GO:0016050, GO:0016197, GO:0016601, GO:0035022, GO:0035023, GO:0035249, GO:0043547, GO:0051036, GO:0071902
ATAD2B	GO:0005634, GO:0003682, GO:0005524, GO:0016887, GO:0070577, GO:0031936, GO:0045944
TBC1D20	GO:0005789, GO:0030173, GO:0031965, GO:0033116, GO:0005096, GO:0017137, GO:0001675, GO:0007030, GO:0019068, GO:0034389, GO:0043547, GO:0044829, GO:0046726, GO:0048208, GO:0070309, GO:0072520, GO:0090110, GO:1902017, GO:1902953
KALRN	GO:0005829, GO:0015629, GO:0070062, GO:0004674, GO:0005089, GO:0005096, GO:0005515, GO:0005524, GO:0046872, GO:0006468, GO:0007399, GO:0016192, GO:0035023, GO:0035556, GO:0043065, GO:0043547, GO:0048013
GNPDA2	GO:0005634, GO:0005829, GO:0004342, GO:0005515, GO:0016787, GO:0006006, GO:0006044
ASB1	GO:0005622, GO:0016567, GO:0030539, GO:0035556, GO:0042036
LANCL1	GO:0005737, GO:0005887, GO:0070062, GO:0003824, GO:0004930, GO:0008270, GO:0017124, GO:0043295, GO:0050750, GO:0007186
STAT5A	GO:0005654, GO:0005829, GO:0003677, GO:0003700, GO:0004713, GO:0004871, GO:0005515, GO:0000255, GO:0001938, GO:0006101, GO:0006103, GO:0006105, GO:0006107, GO:0006351, GO:0006357, GO:0006549, GO:0006573, GO:0006600, GO:0006631, GO:0007595, GO:0018108, GO:0019530, GO:0038161, GO:0040014, GO:0043536, GO:0046449, GO:0060397
MILR1	GO:0005887, GO:0042629, GO:0033004, GO:0043303
FZD8	GO:0005794, GO:0016021, GO:1990851, GO:0004930, GO:0005102, GO:0017147, GO:0030165, GO:0031625, GO:0042813, GO:0000122, GO:0001525, GO:0007186, GO:0030182, GO:0033077, GO:0035567, GO:0043507, GO:0045944, GO:0060070
STAT1	GO:0000790, GO:0005654, GO:0005730, GO:0005829, GO:0030424, GO:0030425, GO:0048471, GO:0000978, GO:0000979, GO:0000983, GO:0004871, GO:0005164, GO:0019899, GO:0042803, GO:0000122, GO:0001937, GO:0002053, GO:0003340, GO:0006351, GO:0006915, GO:0006919, GO:0007259, GO:0007584, GO:0008015, GO:0016525, GO:0032869, GO:0033209, GO:0035458, GO:0043124, GO:0043542, GO:0045944, GO:0046725, GO:0048661, GO:0051591, GO:0051607, GO:0060333, GO:0060334, GO:0060337, GO:0060338, GO:0061326, GO:0071407, GO:0072136, GO:0072162, GO:0072308
MAP3K9	GO:0005622, GO:0016021, GO:0004706, GO:0004708, GO:0005524, GO:0042803, GO:0006351, GO:0006355, GO:0006915, GO:0007256, GO:0007257, GO:0043065, GO:0046777
PPP1R2	GO:0000164, GO:0004865, GO:0005515, GO:0005977, GO:0009966, GO:0043086, GO:0043666
ZC4H2	GO:0005634, GO:0005737, GO:0016021, GO:0030054, GO:0045211, GO:0005515, GO:0046872, GO:0007528, GO:0021522
KCNC1	GO:0008076, GO:0009986, GO:0030425, GO:0030673, GO:0032590, GO:0032809, GO:0005251, GO:0019894, GO:0044325, GO:0009636, GO:0009642, GO:0010996, GO:0014075, GO:0021549, GO:0021759, GO:0035690, GO:0035864, GO:0051260, GO:0051262, GO:0071774, GO:0071805, GO:1901381, GO:1990089
C11orf1	GO:0005654
FAM167A	GO:0005515
CEP41	GO:0005814, GO:0005829, GO:0005886, GO:0005911, GO:0009986, GO:0036064, GO:0072372, GO:0005515, GO:0000086, GO:0015031, GO:0016337,

	GO:0018095, GO:0042384
ADSL	GO:0005739, GO:0005829, GO:0004018, GO:0070626, GO:0006189, GO:0044208, GO:0051262
GLS	GO:0005759, GO:0005829, GO:0004359, GO:0005515, GO:0001967, GO:0002087, GO:0006537, GO:0006543, GO:0007268, GO:0008652, GO:0014047, GO:0051289
ZNF561	GO:0005634, GO:0000977, GO:0003700, GO:0046872, GO:0006351, GO:0006355
EEPD1	GO:0003677, GO:0004519, GO:0004527, GO:0006281, GO:0090305
YY1	GO:0016363, GO:0031011, GO:0031519, GO:0000400, GO:0001078, GO:0001158, GO:0003713, GO:0003714, GO:0003723, GO:0008270, GO:0046332, GO:0000122, GO:0000724, GO:0006351, GO:0006403, GO:0007283, GO:0009952, GO:0010225, GO:0030154, GO:0034644, GO:0048593, GO:0051276
SLC5A3	GO:0005887, GO:0005367, GO:0006020, GO:0006814, GO:0007422, GO:0015798, GO:0043576, GO:0055085
MAN2B2	GO:0043202, GO:0070062, GO:0008270, GO:0008496, GO:0030246, GO:0006013, GO:0006517, GO:0009313
THSD4	GO:0001527, GO:0070062, GO:0004222, GO:0006508, GO:0048251
MGRN1	GO:0005634, GO:0005769, GO:0005829, GO:0005886, GO:0070062, GO:0005515, GO:0008270, GO:0016874, GO:0061630, GO:0000209, GO:0006513, GO:0008333, GO:0043951, GO:0045744
ABCG4	GO:0005886, GO:0016021, GO:0005524, GO:0017127, GO:0034041, GO:0042803, GO:0046982, GO:0033344, GO:0055085
DNAJC25	GO:0016021
MPP2	GO:0005887, GO:0004385, GO:0030165, GO:0007165, GO:0046037, GO:0046710
DZANK1	GO:0046872, GO:0098779
MICAL1	GO:0005737, GO:0005882, GO:0003779, GO:0008270, GO:0016709, GO:0017124, GO:0017137, GO:0071949, GO:0001933, GO:0007165, GO:0019417, GO:0030042, GO:0043154
CD209	GO:0005737, GO:0005886, GO:0009986, GO:0016021, GO:0070062, GO:0001618, GO:0005515, GO:0005537, GO:0042605, GO:0046790, GO:0046872, GO:0002223, GO:0002250, GO:0006897, GO:0007157, GO:0007159, GO:0009988, GO:0019048, GO:0019062, GO:0019079, GO:0035556, GO:0042129, GO:0045087, GO:0046718, GO:0046968, GO:0075733
PREX2	GO:0005622, GO:0005886, GO:0005096, GO:0030676, GO:0007186, GO:0008344, GO:0014065, GO:0035023, GO:0043547, GO:0048813
CLEC12B	GO:0005886, GO:0016021, GO:0030246
ZNF233	GO:0005634, GO:0003677, GO:0046872, GO:0006351, GO:0006355
RPAIN	GO:0005730, GO:0005737, GO:0016605, GO:0032403, GO:0046872, GO:0006261, GO:0006281, GO:0006310, GO:0006606, GO:0009411
PCDH12	GO:0005887, GO:0005911, GO:0070062, GO:0005509, GO:0005977, GO:0007156, GO:0008038, GO:0016339, GO:0060711
ALDH16A1	GO:0016020, GO:0070062, GO:0019145, GO:0047105, GO:0055114
ANKHD1	GO:0005654, GO:0005737, GO:0005515, GO:0044822, GO:0045087
PTPN23	GO:0005654, GO:0005769, GO:0016023, GO:0036064, GO:0070062, GO:0004725, GO:0019901, GO:0010633, GO:0015031, GO:0035335, GO:0043162, GO:0060271, GO:1903387, GO:1903393, GO:2000643
SV2B	GO:0001669, GO:0005886, GO:0016021, GO:0030054, GO:0030672, GO:0005515, GO:0022857, GO:0006836, GO:0055085

SLC35E3	GO:0016021, GO:0005338, GO:1901264, GO:1901679
CPEB1	GO:0000932, GO:0005654, GO:0005813, GO:0014069, GO:0030054, GO:0030425, GO:0030426, GO:0043025, GO:0045211, GO:0048471, GO:1990124, GO:0000166, GO:0000900, GO:0008135, GO:0035925, GO:0043022, GO:0046872, GO:0006397, GO:0006412, GO:0008285, GO:0010976, GO:0030335, GO:0032869, GO:0045727, GO:0051028, GO:0051770, GO:0071222, GO:0071230, GO:0071456, GO:2000766
PDE4DIP	GO:0005634, GO:0005794, GO:0005813, GO:0030016, GO:0019899, GO:0043623
HTR2C	GO:0005829, GO:0005887, GO:0001587, GO:0008144, GO:0051378, GO:0071886, GO:0001662, GO:0006182, GO:0007208, GO:0007268, GO:0007626, GO:0007631, GO:0010513, GO:0031644, GO:0032098, GO:0042493, GO:0043397, GO:0045600, GO:0051209, GO:0051482, GO:0070374
ARHGAP26	GO:0005829, GO:0005856, GO:0005925, GO:0005096, GO:0005515, GO:0005543, GO:0007165, GO:0007399, GO:0030036, GO:0043547, GO:0051056
IGHMBP2	GO:0005634, GO:0005737, GO:0016020, GO:0030424, GO:0030426, GO:0030529, GO:0032797, GO:0000049, GO:0003697, GO:0005524, GO:0008134, GO:0008270, GO:0032575, GO:0043022, GO:0043141, GO:0006260, GO:0006281, GO:0006310, GO:0006351, GO:0006357, GO:0006412, GO:0032508, GO:0051260
DLG2	GO:0008076, GO:0008328, GO:0014069, GO:0016323, GO:0030054, GO:0044224, GO:0045211, GO:0004385, GO:0019900, GO:0035255, GO:0007268, GO:0007399, GO:0010923, GO:0019233, GO:0043113, GO:0045197, GO:0046037, GO:0046710, GO:0097120
COL11A2	GO:0005592, GO:0005788, GO:0030020, GO:0030674, GO:0046872, GO:0001894, GO:0002062, GO:0007605, GO:0030199, GO:0030574, GO:0048705, GO:0060023
OGFOD3	GO:0016021, GO:0070062, GO:0005506, GO:0016705, GO:0031418, GO:0051213, GO:0055114
FCRLB	GO:0005783, GO:0050777
ADGB	GO:0005737, GO:0004198, GO:0019825, GO:0020037, GO:0006508
ADCY2	GO:0005737, GO:0005887, GO:0030425, GO:0045121, GO:0000287, GO:0004016, GO:0005524, GO:0008179, GO:0030145, GO:0046982, GO:0003091, GO:0006171, GO:0007189, GO:0007190, GO:0007193, GO:0019933, GO:0034199, GO:0071377, GO:1904322
DAB2IP	GO:0005829, GO:0030139, GO:0030425, GO:0031235, GO:0032809, GO:0044300, GO:0044301, GO:0070062, GO:1990032, GO:1990597, GO:0005096, GO:0005123, GO:0017124, GO:0031434, GO:0031435, GO:0032266, GO:0032403, GO:0035591, GO:0035662, GO:0036312, GO:0042803, GO:0043184, GO:0051721, GO:0070273, GO:0071889, GO:0000122, GO:0000185, GO:0001525, GO:0006954, GO:0006987, GO:0007049, GO:0007252, GO:0007257, GO:0008625, GO:0010596, GO:0010719, GO:0014067, GO:0016525, GO:0021814, GO:0021819, GO:0030948, GO:0032088, GO:0034144, GO:0034260, GO:0035148, GO:0035414, GO:0035924, GO:0036324, GO:0038026, GO:0040008, GO:0042059, GO:0043124, GO:0043254, GO:0043407, GO:0043497, GO:0043547, GO:0043553, GO:0044257, GO:0045087, GO:0045944, GO:0046580, GO:0048147, GO:0048812, GO:0050680, GO:0070059, GO:0070317, GO:0070373, GO:0071158, GO:0071222, GO:0071347, GO:0071356, GO:0071364, GO:0072577, GO:0090090, GO:0090129, GO:1900006, GO:1900744, GO:1900747, GO:1901800, GO:1903896, GO:2001224, GO:2001235
MYO18A	GO:0000139, GO:0005654, GO:0005793, GO:0005802, GO:0016459, GO:0042641, GO:0003677, GO:0003774, GO:0005524, GO:0008094, GO:0043531, GO:0044822, GO:0051015, GO:0006259, GO:0016477, GO:0031032, GO:0043066, GO:0048194, GO:0050714, GO:0090164
FBXL20	GO:0005737, GO:0005515, GO:0001662
MAPT	GO:0005829, GO:0005874, GO:0005875, GO:0005886, GO:0005930, GO:0014069, GO:0030424, GO:0030425, GO:0030426, GO:0034399, GO:0036464, GO:0044297, GO:0045298, GO:0097418, GO:0005200, GO:0008017, GO:0017124, GO:0019901, GO:0032403, GO:0034185, GO:0051721, GO:0071813, GO:0000226, GO:0001764, GO:0007420, GO:0007565, GO:0007584, GO:0007628, GO:0008088, GO:0008631, GO:0010033, GO:0010506, GO:0031116,

	GO:0032387, GO:0045773, GO:0047497, GO:0048675, GO:0060632
PAG1	GO:0005622, GO:0005887, GO:0045121, GO:0005068, GO:0005070, GO:0042169, GO:0002250, GO:0007173, GO:0009967, GO:0035556, GO:0050852, GO:0050868
PLEKHH2	GO:0005886, GO:0030027, GO:0030864, GO:0003779, GO:0042802, GO:0030835
ADCY1	GO:0005634, GO:0005737, GO:0005887, GO:0045121, GO:0070062, GO:0005516, GO:0005524, GO:0008294, GO:0046872, GO:0003091, GO:0006171, GO:0007189, GO:0007190, GO:0007193, GO:0007409, GO:0007616, GO:0007623, GO:0010226, GO:0019933, GO:0034199, GO:0042493, GO:0042752, GO:0071277, GO:0071377, GO:1904322
CAMSAP3	GO:0005737, GO:0005813, GO:0005874, GO:0005915, GO:0005516, GO:0030507, GO:0051011, GO:0010923, GO:0031175, GO:0034453, GO:0045218, GO:0070507, GO:0090136
HIST1H4I	GO:0000784, GO:0000786, GO:0005654, GO:0016020, GO:0070062, GO:0003677, GO:0019904, GO:0042393, GO:0044822, GO:0046982, GO:0000088, GO:0000183, GO:0006303, GO:0006335, GO:0006352, GO:0016233, GO:0031047, GO:0034080, GO:0044267, GO:0045653, GO:0045815, GO:0051290, GO:1904837
GGA2	GO:0005802, GO:0010008, GO:0030131, GO:0030136, GO:0030306, GO:0006886, GO:0016192, GO:0044267
ANKRD40	GO:0005515
OR51D1	GO:0005886, GO:0016021, GO:0004930, GO:0004984, GO:0007186, GO:0050911
GARNL3	GO:0005096, GO:0043547, GO:0051056
ZNF2	GO:0005634, GO:0003677, GO:0003700, GO:0005515, GO:0008270, GO:0006351, GO:0006355
R3HCC1	GO:0035145, GO:0000166, GO:0003676
RINT1	GO:0005789, GO:0005829, GO:0070939, GO:0005515, GO:0006890, GO:0007049, GO:0015031, GO:0060628, GO:1902504
SLC6A4	GO:0005829, GO:0005887, GO:0010008, GO:0043005, GO:0045121, GO:0098793, GO:0005335, GO:0017022, GO:0017075, GO:0017137, GO:0019811, GO:0042803, GO:0046872, GO:0050998, GO:0051015, GO:0001666, GO:0007268, GO:0007584, GO:0007613, GO:0007623, GO:0009636, GO:0010628, GO:0021794, GO:0021941, GO:0032227, GO:0032355, GO:0035176, GO:0042310, GO:0042493, GO:0042713, GO:0045665, GO:0045787, GO:0046621, GO:0048854, GO:0051260, GO:0051610, GO:0055085, GO:0071300, GO:0071321
ANGPTL6	GO:0030141, GO:0070062, GO:0001525, GO:0030154
STARD5	GO:0005829, GO:0032052, GO:0006700, GO:0006869
OR13C9	GO:0005886, GO:0016021, GO:0004930, GO:0004984, GO:0007186, GO:0050911
SV2C	GO:0005886, GO:0016021, GO:0030054, GO:0030672, GO:0022857, GO:0006836, GO:0055085
NAGLU	GO:0043202, GO:0070062, GO:0004561, GO:0006027, GO:0007040, GO:0021680, GO:0042474, GO:0045475, GO:0046548, GO:0060119
GPR15	GO:0005887, GO:0004930, GO:0007186, GO:0072678
PRELP	GO:0005578, GO:0005615, GO:0005796, GO:0043202, GO:0070062, GO:0005201, GO:0008201, GO:0001501, GO:0007409, GO:0007569, GO:0018146, GO:0042340
PLEKHG4	GO:0005089, GO:0005515, GO:0035023, GO:0090630
SCUBE1	GO:0005615, GO:0009897, GO:0019897, GO:0005509, GO:0042802, GO:0046982, GO:0006954, GO:0007512, GO:0007596, GO:0009791, GO:0045446,

	GO:0051260
TNS1	GO:0005737, GO:0005856, GO:0005925, GO:0009986, GO:0003779, GO:0044822, GO:0007044, GO:0010761
DGCR8	GO:0005654, GO:0005730, GO:0005737, GO:0015630, GO:0070877, GO:0003725, GO:0004525, GO:0020037, GO:0042803, GO:0046872, GO:0070878, GO:0031053, GO:0072091, GO:0090502
WHSC1	GO:0005654, GO:0005694, GO:0005737, GO:0003682, GO:0005515, GO:0008270, GO:0042799, GO:0043565, GO:0000122, GO:0003149, GO:0003289, GO:0003290, GO:0006303, GO:0006351, GO:0010452, GO:0034770, GO:0048298, GO:0060348, GO:0070201, GO:2001032
DGKQ	GO:0005829, GO:0005856, GO:0005886, GO:0016607, GO:0004143, GO:0005524, GO:0019900, GO:0033613, GO:0043274, GO:0046872, GO:0006357, GO:0007205, GO:0016310, GO:0019933, GO:0030168, GO:0033198, GO:0070493, GO:0070528
HRNR	GO:0001533, GO:0005634, GO:0030313, GO:0036457, GO:0048471, GO:0070062, GO:0005509, GO:0002244, GO:0031424, GO:0043163, GO:0061436
PDCD6	GO:0005768, GO:0005789, GO:0031410, GO:0031965, GO:0070062, GO:0070971, GO:0004198, GO:0005509, GO:0042803, GO:0043495, GO:0048306, GO:0060090, GO:0001525, GO:0001938, GO:0006886, GO:0006915, GO:0010595, GO:0030948, GO:0032007, GO:0034605, GO:0036324, GO:0045766, GO:0051592, GO:0051898, GO:0097190
SOX15	GO:0005737, GO:0044798, GO:0000981, GO:0003677, GO:0003682, GO:0046982, GO:0000122, GO:0006325, GO:0008584, GO:0014718, GO:0043403, GO:0045843, GO:0045944, GO:0048627, GO:0070318, GO:2000288
NDUFAB1	GO:0005747, GO:0005759, GO:0000036, GO:0005504, GO:0005509, GO:0008137, GO:0006120, GO:0006633, GO:0009249, GO:0032981, GO:0046487
AHI1	GO:0005814, GO:0005829, GO:0005911, GO:0005912, GO:0031513, GO:0036038, GO:0036064, GO:0042802, GO:0001738, GO:0001947, GO:0002092, GO:0007169, GO:0010842, GO:0016192, GO:0030862, GO:0030902, GO:0034613, GO:0035844, GO:0035845, GO:0039008, GO:0039023, GO:0042384, GO:0043066, GO:0045944, GO:0050795, GO:0065001, GO:0070121, GO:0070986, GO:0071599
SLC38A1	GO:0005887, GO:0030424, GO:0070062, GO:0005283, GO:0005290, GO:0005515, GO:0015182, GO:0015186, GO:0001504, GO:0006814, GO:0006867, GO:0006868, GO:0015807, GO:0015817, GO:0089709
MYSM1	GO:0005634, GO:0003677, GO:0003713, GO:0004843, GO:0008237, GO:0032403, GO:0042393, GO:0046872, GO:0006338, GO:0006351, GO:0030334, GO:0035522, GO:0045944, GO:0051797
C8orf4	GO:0005730, GO:0005737, GO:0016607, GO:0005112, GO:0002264, GO:0006915, GO:0034605, GO:0042346, GO:0043620, GO:0045746, GO:1902806, GO:1903706
SASH1	GO:0043234, GO:0008022, GO:0031435, GO:0032947, GO:0000209, GO:0010595, GO:0031666, GO:0043507, GO:0045766, GO:1900044, GO:1900745, GO:1901224, GO:1902498
FREM2	GO:0005604, GO:0005886, GO:0016021, GO:0070062, GO:0046872, GO:0002009, GO:0007154, GO:0007155, GO:0007507, GO:0048839
ZC3H14	GO:0005730, GO:0005737, GO:0016607, GO:0005515, GO:0008143, GO:0044822, GO:0046872, GO:0043488, GO:1900364
BAZ2A	GO:0005677, GO:0005730, GO:0005737, GO:0033553, GO:0001164, GO:0003723, GO:0008270, GO:0016922, GO:0070577, GO:0000183, GO:0006306, GO:0006338, GO:0006351, GO:0016575
SLC4A2	GO:0005887, GO:0005925, GO:0016323, GO:0016324, GO:0005452, GO:0015301, GO:0019899, GO:0007283, GO:0015701, GO:0048565, GO:0051453, GO:1902476
SLC31A2	GO:0005770, GO:0005887, GO:0055037, GO:0005375, GO:0006878, GO:0035434, GO:1902311
TMSB15A	GO:0005737, GO:0005856, GO:0003785, GO:0007015

BCL7B	GO:0003779, GO:0006915, GO:0016055, GO:0030154
TRPC7	GO:0005635, GO:0005801, GO:0005887, GO:0048471, GO:0005515, GO:0015279, GO:0006828, GO:0007338, GO:0030168, GO:0051480, GO:0070588
WRNIP1	GO:0000784, GO:0016020, GO:0048471, GO:0003677, GO:0005524, GO:0016887, GO:0042802, GO:0046872, GO:0000731, GO:0006260, GO:0030174
AMMECR1L	GO:0016021
CSRNP1	GO:0005634, GO:0001228, GO:0005515, GO:0043565, GO:0006366, GO:0006915, GO:0009791, GO:0045944, GO:0048008, GO:0048705, GO:0060021, GO:0060325
NXF1	GO:0000346, GO:0005643, GO:0005829, GO:0016607, GO:0042405, GO:0000166, GO:0003727, GO:0003729, GO:0005487, GO:0005515, GO:0016032, GO:0016973
MED24	GO:0000151, GO:0016592, GO:0001104, GO:0004402, GO:0004872, GO:0030374, GO:0042809, GO:0046966, GO:0061630, GO:0006357, GO:0006367, GO:0016567, GO:0016573, GO:0019827, GO:0030521, GO:0045893, GO:0051291
PRSS21	GO:0005737, GO:0005886, GO:0031225, GO:0004252, GO:0005515, GO:0006508, GO:0007283
MKL2	GO:0005634, GO:0001105, GO:0005515, GO:0006351, GO:0007517, GO:0045844, GO:0045944, GO:0051145
TTLL3	GO:0005737, GO:0005874, GO:0005930, GO:0005524, GO:0070736, GO:0018094, GO:0035082
SH3TC2	GO:0005886, GO:0031410, GO:0055037, GO:0032287, GO:0033157, GO:1901184
RREB1	GO:0005730, GO:0005737, GO:0016607, GO:0070062, GO:0000979, GO:0003700, GO:0046872, GO:0000122, GO:0006366, GO:0007265, GO:0007275, GO:0010634, GO:0033601, GO:0045893, GO:1900026, GO:1903691, GO:2000394
NUP85	GO:0000777, GO:0005819, GO:0005829, GO:0031080, GO:0031965, GO:0005515, GO:0017056, GO:0000090, GO:0000236, GO:0006406, GO:0006409, GO:0006606, GO:0007062, GO:0007077, GO:0010827, GO:0016925, GO:0019083, GO:0030032, GO:0031047, GO:0045893, GO:0048246, GO:0075733, GO:1900034
LNPEP	GO:0005576, GO:0005765, GO:0005829, GO:0005887, GO:0030659, GO:0031905, GO:0048471, GO:0005515, GO:0008270, GO:0042277, GO:0070006, GO:0000209, GO:0002480, GO:0006508, GO:0007267, GO:0007565, GO:0008217, GO:0030163, GO:0043171, GO:0060395, GO:0061024
MED22	GO:0005737, GO:0016592, GO:0001104, GO:0005515, GO:0006351, GO:0006357
APPBP2	GO:0005634, GO:0005874, GO:0005875, GO:0030659, GO:0003777, GO:0008017, GO:0006886
SIK2	GO:0005634, GO:0005737, GO:0000287, GO:0004674, GO:0005515, GO:0005524, GO:0032870, GO:0035556, GO:0046626, GO:0046777
XPO5	GO:0005654, GO:0005829, GO:0042565, GO:0000049, GO:0003729, GO:0008536, GO:0008565, GO:0070883, GO:0090631, GO:0006611, GO:0035281, GO:1900370
NCKAP1L	GO:0005829, GO:0005887, GO:0031209, GO:0070062, GO:0005096, GO:0030295, GO:0032403, GO:0001782, GO:0006461, GO:0030011, GO:0030593, GO:0030838, GO:0030866, GO:0030890, GO:0032147, GO:0032700, GO:0032715, GO:0033630, GO:0035509, GO:0038096, GO:0042102, GO:0042493, GO:0043029, GO:0043066, GO:0043372, GO:0043378, GO:0043547, GO:0045579, GO:0045588, GO:0045648, GO:0048010, GO:0048821, GO:0050853, GO:0060100, GO:0070358, GO:0090023
GANC	GO:0004558, GO:0030246, GO:0032450, GO:0000023
DGCR2	GO:0016021, GO:0030246, GO:0007155, GO:0009887, GO:0050890
CFAP54	GO:0005737, GO:0005930, GO:0007283, GO:0030154, GO:0042384, GO:0060294

SLC7A6	GO:0005887, GO:0016323, GO:0015179, GO:0015297, GO:0006461, GO:0006520, GO:0015807, GO:0050900, GO:1902475
THADA	GO:0005515
INF2	GO:0048471, GO:0003779, GO:0017048, GO:0030036, GO:0090140
CDC25A	GO:0005654, GO:0005829, GO:0004725, GO:0019901, GO:0000079, GO:0000082, GO:0000084, GO:0000086, GO:0006260, GO:0007067, GO:0008283, GO:0034644, GO:0035335, GO:0051301
GDPD5	GO:0016021, GO:0030424, GO:0030426, GO:0043025, GO:0097038, GO:0005515, GO:0008889, GO:0047389, GO:0006629, GO:0021522, GO:0021895, GO:0031175, GO:0045666, GO:0045746, GO:0045787, GO:0048505
STAT5B	GO:0005654, GO:0005829, GO:0000979, GO:0001077, GO:0003682, GO:0004713, GO:0004871, GO:0019903, GO:0035259, GO:0046983, GO:0000255, GO:0001553, GO:0001666, GO:0001779, GO:0001889, GO:0006101, GO:0006103, GO:0006105, GO:0006107, GO:0006366, GO:0006549, GO:0006573, GO:0006600, GO:0006631, GO:0006953, GO:0007565, GO:0007595, GO:0018108, GO:0019218, GO:0019530, GO:0019915, GO:0030856, GO:0032355, GO:0032496, GO:0032819, GO:0032825, GO:0033077, GO:0038161, GO:0040018, GO:0042104, GO:0042448, GO:0043029, GO:0043066, GO:0045086, GO:0045471, GO:0045579, GO:0045588, GO:0045647, GO:0045931, GO:0045944, GO:0045954, GO:0046449, GO:0046543, GO:0046544, GO:0048541, GO:0048661, GO:0050729, GO:0051272, GO:0060397, GO:0070669, GO:0070670, GO:0070672, GO:0071364, GO:0097531
TRIM59	GO:0005789, GO:0005813, GO:0005929, GO:0016021, GO:0030992, GO:0005515, GO:0008270, GO:0061630, GO:0016567, GO:0042384, GO:0043124, GO:0045087, GO:0046597
VWA1	GO:0005604, GO:0005614, GO:0005615, GO:0070062, GO:0042802, GO:0030198, GO:0048266
ITGB4	GO:0005634, GO:0008305, GO:0009986, GO:0030056, GO:0031252, GO:0070062, GO:0001664, GO:0004872, GO:0031994, GO:0038132, GO:0006914, GO:0007160, GO:0007229, GO:0009611, GO:0030198, GO:0031581, GO:0035878, GO:0043588, GO:0048333, GO:0048565, GO:0048870, GO:0072001, GO:0097186
XYLT1	GO:0000139, GO:0005576, GO:0005789, GO:0016021, GO:0008375, GO:0030158, GO:0015012, GO:0030206
MS4A7	GO:0016021
BRF1	GO:0000126, GO:0005654, GO:0003743, GO:0008270, GO:0017025, GO:0006355, GO:0006384, GO:0006413, GO:0009303, GO:0009304
KIAA2013	GO:0016021
RNF40	GO:0005654, GO:0005829, GO:0019898, GO:0033503, GO:0043679, GO:0003730, GO:0004842, GO:0008270, GO:0016874, GO:0017075, GO:0031624, GO:0031625, GO:0032403, GO:0042803, GO:0006511, GO:0007346, GO:0010390, GO:0033523, GO:0043434, GO:1900364, GO:1901800, GO:1902916, GO:2001168
UQCC1	GO:0005743, GO:0016023, GO:0005515, GO:0034551, GO:0070131
SLC11A1	GO:0005764, GO:0005887, GO:0009279, GO:0030670, GO:0031902, GO:0070821, GO:0005384, GO:0042803, GO:0051139, GO:0000060, GO:0001818, GO:0002309, GO:0002369, GO:0002606, GO:0002827, GO:0006826, GO:0006876, GO:0006879, GO:0006909, GO:0006954, GO:0007035, GO:0015707, GO:0032147, GO:0032496, GO:0032623, GO:0032632, GO:0032729, GO:0034341, GO:0042060, GO:0042116, GO:0042832, GO:0043091, GO:0045342, GO:0045454, GO:0045730, GO:0045944, GO:0048002, GO:0048255, GO:0050766, GO:0050829, GO:0060586, GO:0070574, GO:0070839, GO:0071421
H2BFWT	GO:0000788, GO:0031965, GO:0003677, GO:0046982, GO:0006334
ITGA3	GO:0005925, GO:0009897, GO:0016323, GO:0030426, GO:0031527, GO:0034667, GO:0048471, GO:0060076, GO:0070062, GO:0071438, GO:0097060, GO:0001948, GO:0001968, GO:0002020, GO:0005178, GO:0005518, GO:0019904, GO:0043236, GO:0046872, GO:0046982, GO:0001764, GO:0007160,

	GO:0007229, GO:0007507, GO:0007613, GO:0010628, GO:0010634, GO:0010811, GO:0010976, GO:0017015, GO:0030111, GO:0030198, GO:0030324, GO:0030510, GO:0031345, GO:0034698, GO:0035024, GO:0035640, GO:0042493, GO:0043588, GO:0048333, GO:0050900, GO:0060135, GO:0072006, GO:0090004, GO:0097062, GO:0097205
CDH23	GO:0005829, GO:0005886, GO:0016021, GO:0032420, GO:0005509, GO:0005515, GO:0006816, GO:0007156, GO:0007601, GO:0007605, GO:0007626, GO:0016339, GO:0045494, GO:0050896, GO:0050957, GO:0051480, GO:0060122
HSPA12A	GO:0070062, GO:0005524
CTDSPL2	GO:0005654, GO:0004721, GO:0006470, GO:0030514, GO:0046827
XYLT2	GO:0000139, GO:0005789, GO:0016021, GO:0008375, GO:0030158, GO:0015012, GO:0030206, GO:0030210
TBXAS1	GO:0005789, GO:0016021, GO:0004497, GO:0004796, GO:0005506, GO:0016705, GO:0020037, GO:0036134, GO:0019371, GO:0055114
NOX5	GO:0005789, GO:0016021, GO:0005509, GO:0005515, GO:0015252, GO:0016175, GO:0020037, GO:0050660, GO:0050661, GO:0000302, GO:0000910, GO:0001525, GO:0001935, GO:0006915, GO:0010155, GO:0015992, GO:0042554, GO:0043012, GO:0050663, GO:0055114, GO:2000379
KSR1	GO:0005789, GO:0005829, GO:0043234, GO:0004672, GO:0005078, GO:0005524, GO:0008022, GO:0031434, GO:0046872, GO:0000165, GO:0007265, GO:0019933, GO:0043410
ZNF500	GO:0005654, GO:0005737, GO:0003700, GO:0043565, GO:0046872, GO:0006351, GO:0006355
KDELR1	GO:0000139, GO:0005789, GO:0005801, GO:0016021, GO:0030133, GO:0030663, GO:0033116, GO:0005046, GO:0006621, GO:0006886, GO:0006888, GO:0006890
TAS2R16	GO:0005783, GO:0005802, GO:0009897, GO:0016021, GO:0004930, GO:0005515, GO:0033038, GO:0001580, GO:0007186
GPR37	GO:0000151, GO:0005789, GO:0005887, GO:0043235, GO:0008528, GO:0030544, GO:0031625, GO:0036505, GO:0042277, GO:0007193, GO:0031987, GO:0042416, GO:0043410, GO:0045964, GO:1903206
NISCH	GO:0005769, GO:0005829, GO:0005886, GO:0055037, GO:0005178, GO:0035091, GO:0042802, GO:0006006, GO:0006915, GO:0008217, GO:0016601, GO:0030036, GO:0030336, GO:0032228, GO:0048243
KCNH8	GO:0005622, GO:0005887, GO:0000155, GO:0005249, GO:0000160, GO:0023014, GO:0034765, GO:0042391, GO:0071805
SWSAP1	GO:0005634, GO:0097196, GO:0003697, GO:0005515, GO:0016887, GO:0000724, GO:0050821
LAMTOR1	GO:0005765, GO:0005794, GO:0005886, GO:0070062, GO:0071986, GO:0005085, GO:0032947, GO:0001919, GO:0007032, GO:0007040, GO:0007050, GO:0010872, GO:0010874, GO:0016049, GO:0016236, GO:0032008, GO:0032418, GO:0032439, GO:0034613, GO:0042632, GO:0043410, GO:0043547, GO:0060620, GO:0071230
KRT76	GO:0005634, GO:0045095, GO:0070062, GO:0005198, GO:0007010, GO:0008544, GO:0048733
AGFG2	GO:0016020, GO:0005096, GO:0046872, GO:0043547
CCT8L2	GO:0005737, GO:0005253, GO:0005524, GO:0015269, GO:0071805, GO:0098656
FCRL2	GO:0005886, GO:0016021, GO:0005070, GO:0007267, GO:0009967
B4GALT1	GO:0000138, GO:0005615, GO:0009897, GO:0016021, GO:0016323, GO:0030057, GO:0030112, GO:0030175, GO:0031526, GO:0032580, GO:0070062, GO:0003831, GO:0003945, GO:0004461, GO:0019901, GO:0030145, GO:0042803, GO:0043014, GO:0048487, GO:0002064, GO:0002526, GO:0005989, GO:0006012, GO:0006487, GO:0007155, GO:0007339, GO:0007341, GO:0008285, GO:0018146, GO:0030198, GO:0030879, GO:0045136, GO:0048754,

	GO:0050900, GO:0051270, GO:0060046, GO:0060054, GO:0060055, GO:0060058
NIPBL	GO:0000785, GO:0005654, GO:0032116, GO:0070062, GO:0003682, GO:0008022, GO:0036033, GO:0042826, GO:0047485, GO:0070087, GO:0000122, GO:0000910, GO:0001656, GO:0003151, GO:0006974, GO:0007420, GO:0007605, GO:0019827, GO:0031065, GO:0034088, GO:0034613, GO:0035115, GO:0035261, GO:0040018, GO:0042471, GO:0042634, GO:0045444, GO:0045778, GO:0045944, GO:0045995, GO:0048557, GO:0048589, GO:0048592, GO:0048703, GO:0050890, GO:0060325, GO:0061010, GO:0061038, GO:0071481
SERF2	GO:0005634, GO:0005829
